# UBE2A and UBE2B are recruited by an atypical E3 ligase module in UBR4

**DOI:** 10.1038/s41594-023-01192-4

**Published:** 2024-01-05

**Authors:** Lucy Barnsby-Greer, Peter D. Mabbitt, Marc-Andre Dery, Daniel R. Squair, Nicola T. Wood, Frederic Lamoliatte, Sven M. Lange, Satpal Virdee

**Affiliations:** 1 MRC Protein Phosphorylation and Ubiquitylation Unit, University of Dundee, Scotland, UK; 2 Scion, Rotorua, New Zealand

**Keywords:** Ligases, X-ray crystallography, Ubiquitin ligases, Ubiquitylation, Ubiquitylation

## Abstract

UBR4 is a 574 kDa E3 ligase (E3) of the N-degron pathway with roles in neurodevelopment, age-associated muscular atrophy and cancer. The catalytic module that carries out ubiquitin (Ub) transfer remains unknown. Here we identify and characterize a distinct E3 module within human UBR4 consisting of a ‘hemiRING’ zinc finger, a helical-rich UBR zinc-finger interacting (UZI) subdomain, and an N-terminal region that can serve as an affinity factor for the E2 conjugating enzyme (E2). The structure of an E2–E3 complex provides atomic-level insight into the specificity determinants of the hemiRING toward the cognate E2s UBE2A/UBE2B. Via an allosteric mechanism, the UZI subdomain modestly activates the Ub-loaded E2 (E2∼Ub). We propose attenuated activation is complemented by the intrinsically high lysine reactivity of UBE2A, and their cooperation imparts a reactivity profile important for substrate specificity and optimal degradation kinetics. These findings reveal the mechanistic underpinnings of a neuronal N-degron E3, its specific recruitment of UBE2A, and highlight the underappreciated architectural diversity of cross-brace domains with Ub E3 activity.

## Abstract (web summary)

The ubiquitin E3 ligase UBR4 is a key component of the ubiquitin N-degron pathway, but the domain that catalyzes ubiquitin transfer remains unknown. Here the authors identify its unorthodox E3 module and characterize its structure and ubiquitin transfer mechanism.

## Main

Covalent attachment of the small protein ubiquitin (Ub), typically to lysine residues in protein substrates, regulates a host of cellular processes and is catalyzed by E3 ligases (E3s). Approximately 700 E3s have been identified, but the functions and catalytic mechanisms have only been established for a few representative members. Fundamental arms of the Ub system are the N-degron and C-degron pathways, which control proteasomal or autophagic substrate degradation based on the identity of their N- or C-terminal amino acid^1^. The regulated degradation of substrates by the N-/C-degron pathways affects multiple cellular processes, including the elimination of misfolded or mislocalized proteins, maintenance of protein complex stoichiometry, DNA repair, apoptosis, metabolite sensing and neurodevelopment^1^. N-degrons are recognized by an ∼70 residue zinc-finger domain known as the UBR box^2,3^.

In mammals there are seven UBR box-containing proteins, termed UBR1 to UBR7, that function as E3s and are highly diverse in structure and mechanism. The E3 module responsible for ubiquitination activity in UBR1 to UBR3 is the RING domain^1,2,4^. This is a small (∼10 kDa) fold that uses a cross-brace configuration to coordinate two Zn^2+^ ions^5^. A related domain is the U-box, which achieves a similar structure through hydrogen-bonding^6^. Using an allosteric mechanism, RING/U-box E3s catalyze Ub transfer to substrates from an upstream thioester-linked E2 conjugating enzyme (E2∼Ub). This involves stabilization of a reactive closed E2∼Ub conformation^7–12^. For optimum stabilization, and in turn high substrate transfer activity, allosteric E3s lock E2∼Ub into the closed conformation via electrostatic interactions mediated by a so-called linchpin residue. A hydrophobic interaction with the Ile36 residue of Ub augments stabilization. However, RING-containing E3s can be devoid of allosteric activity as RING-in-between-RING (RBR) and RING-Cys-Relay (RCR) E3 subtypes use an ancillary domain, harboring an essential active site cysteine, that covalently receives Ub from E2∼Ub before substrate ubiquitination^13,14^. UBR5 belongs to the Homologous to E6AP Carboxy-Terminus (HECT) E3 subtype, and like RBR/RCR E3s, has an active site cysteine in its HECT domain^15^. Further mechanistic divergence within the UBR family is demonstrated by UBR6 (more commonly known as FBXO11), which is a substrate receptor of a multi-subunit Cullin RING E3 complex^16^, whereas UBR7 contains a plant homeodomain with E3 activity^16,17^.

UBR4 is an essential protein ubiquitously expressed but highly enriched in the central nervous system. It has several prominent functions in the mammalian brain, including neurogenesis, neuronal migration, neuronal survival and signaling^18–22^. UBR4 has also been implicated in anoikis, viral transformation and the endosome–lysosome system^23–26^. Disease associations include neurological disorders and myofiber atrophy^27–29^, and UBR4 loss increases cancer cell susceptibility to apoptosis^23,30^. The latter is probably due to the proteotoxicity from imbalanced protein complex stoichiometry arising from aneuploidy—a hallmark of many cancers. Mechanistically, little is known about UBR4, and none of its domains have been structurally characterized. Furthermore, UBR4 does not contain a predictable E3 module, so the source of its E3 activity remains a mystery. This highlights a notable gap in our molecular understanding of this protein and the N-degron pathway in general. In this Article, we locate the source of E3 activity and characterize the module using biochemical methods and X-ray crystallography.

## Results

### The UBR4 E3 module is catalytically functional with UBE2A/UBE2B

**Fig. 1:**
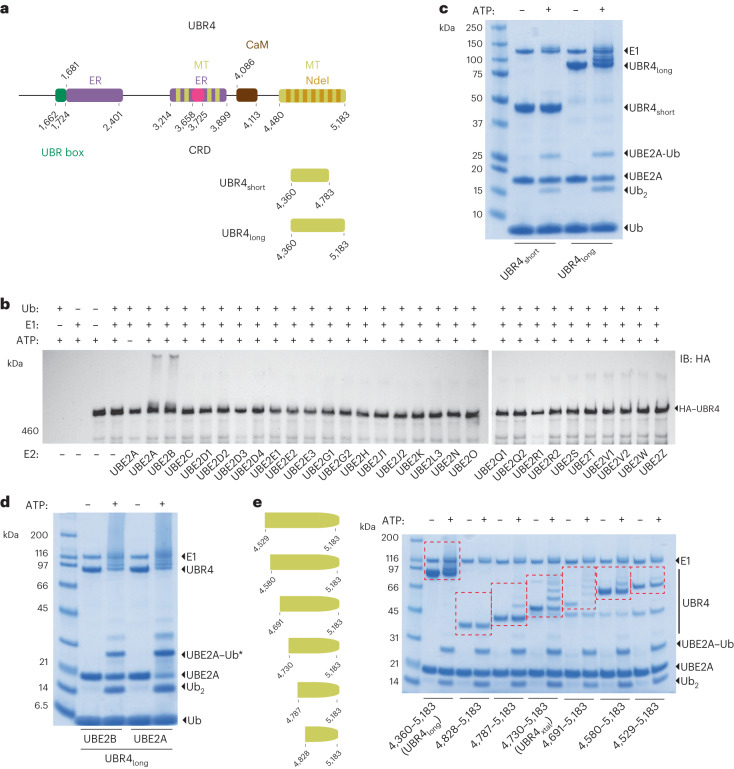
Domain architecture of UBR4 and assessment of UBR4 E3 activity. **a**, Domain architecture of UBR4. UBR box, UBR box domain; ER, endoplasmic reticulum-associated region; MT, microtubule-binding region; CRD, cysteine-rich domain; CaM, calmodulin-binding domain; Ndel1, Ndel1-binding region^19,31^. Regions in alternating-colored bars correspond to those with more than one experimentally observed interaction. Two C-terminal constructs were studied to search for the E3 module, UBR4_short_ and UBR4_long_. **b**, An E2 panel was screened to identify those cooperating with UBR4. A stable HEK293 cell line expressing full-length HA–UBR4 was immunoprecipitated with anti-HA sepharose resin. The washed resin was combined with E1 (500 nM), Ub (5 µM), ATP (10 mM) and the specified E2 (1.5–10 µM). Reactions were incubated at 37 °C for 1 h, stopped by the addition of reducing lithium dodecyl sulfate (LDS) loading buffer, and visualized by anti-HA immunoblot (IB). Autoubiquitination reactions were resolved by reducing SDS–PAGE and visualized by Coomassie staining. **c**, Activity assessment of UBR4_short_ and UBR4_long_ with UBE2A. Autoubiquitination reactions were resolved by reducing SDS–PAGE and visualized by Coomassie staining. **d**, UBR4_long_ demonstrated comparable autoubiquitination activity with UBE2A and UBE2B. SDS–PAGE was carried out using nonreducing conditions. **e**, Progressive N-terminal truncations of UBR4_long_ were tested for autoubiquitination activity. The UBR4 construct and its autoubiquitination products are highlighted with red hashed boxes. SDS–PAGE was carried out under reducing conditions. E2–Ub is isopeptide linked, E2–Ub* is a mixture of isopeptide and thioester linkages, and E2∼Ub is thioester linked. Experiments in **b**–**e** were performed two to three times with similar results. Source data

**Extended Data Fig. 1:**
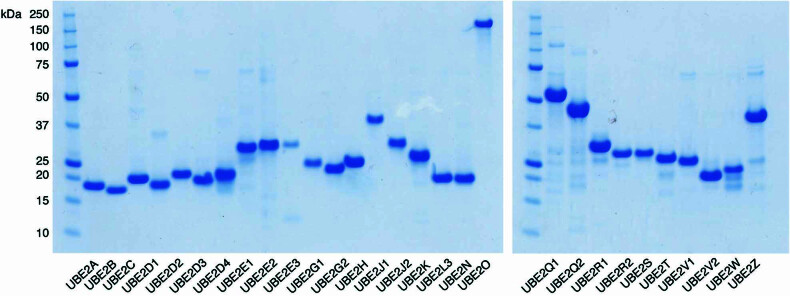
Recombinant panel of E2 conjugating enzymes used in UBR4 activity assay. E2s were expressed in *E. coli* and purified by affinity chromatography using established methods. UBE2A, UBE2B, UBE2C, UBE2D1, UBE2D4, UBE2L3, UBE2R2 and UBE2S were untagged whereas the remaining E2s bared N-terminal 6xhistidine tags. Analysis was carried out once.

UBR4 is one of the largest known single-subunit proteins, consisting of over 5,000 residues (Fig. 1a)^31^. To establish the mechanistic subtype and location of the E3 module, we initially tested which E2s support UBR4 E3 activity. Although UBR4 has been shown to bind the E2 UBE2A, this interaction has yet to be functionally tested^32^. The E2 activity profile of UBR4 could also inform on whether it uses an allosteric or catalytic cysteine-dependent mechanism^13^. UBR4 is a large multidomain protein, so to ensure a comprehensive assessment of E3 activity, we immunoprecipitated full-length hemagglutinin (HA)-tagged UBR4 stably expressed in HEK293 cells. Its autoubiquitination activity was then measured with a recombinant panel of 29 Ub E2s (Extended Data Fig. 1). HA–UBR4 underwent robust autoubiquitination activity, but only when partnered with UBE2A, or its paralogue, UBE2B (Fig. 1b). Furthermore, autoubiquitination was absent with UBE2L3, an E2 that cannot support allosteric E3 activity^13^.

To locate the E3 module, which often exists at or near the C-terminus, we investigated two C-terminal recombinant fragments amenable to expression in *Escherichia coli*: a 93 kDa construct consisting of the C-terminal 823 residues (UBR4_long_; residues 4,360–5,183), and a 47 kDa C-terminal truncated version (UBR4_short_; residues 4,360–4,783), both of which are within the second microtubule-binding region of UBR4 (Fig. 1a). UBR4_long_ underwent robust autoubiquitination when partnered with UBE2A, but lack of activity with UBR4_short_ revealed the functional importance of the C-terminal 400 residues (Fig. 1c). UBR4_long_ was similarly active with UBE2B (Fig. 1d). Progressive N-terminal truncation of UBR4_long_ identified UBR4_4730–5183_ as the smallest active construct (Fig. 1e). A caveat with autoubiquitination assays is they do not discern whether loss of autoubiquitination is from catalytic impairment or loss of autoubiquitination sites. Nevertheless, we focused on UBR4_4730–5183_ for further characterization.

### Low E3 activity complements high UBE2A lysine reactivity

**Extended Data Fig. 2:**
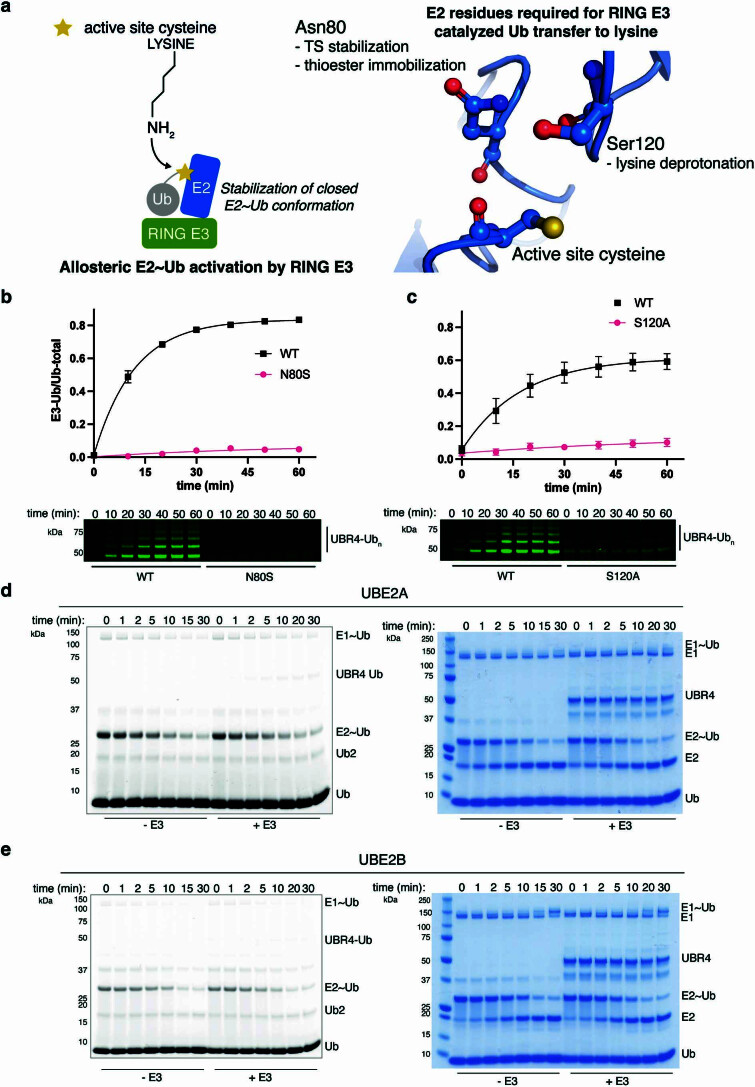
Essentiality of UBE2A residues Asn80 and Ser120 imply that UBR4 uses an allosteric mechanism. **a)** Schematic illustrating the importance of UBE2A residues Asn80 and Ser120 in adapter-like E3 catalyzed Ub transfer. Adapter-like E3s stabilize a closed E2∼Ub conformation to facilitate Ub transfer to lysine. In this context, residue Asn80 is essential in thioester activation and/or transition state stabilization. Residue Ser120 positions and/or deprotonates the lysine acceptor within substrates. **b)** Quantitative UBR4 autoubiquitination assay comparing UBE2A WT with UBE2A Asn80Ser under single turnover E2∼Ub discharge conditions. The mean from independent experiments is plotted and bars correspond to standard error (*n* = 3). **c)** Autoubiquitination assay comparing UBE2A WT with UBE2A Ser120Ala. The mean from independent experiments is plotted and bars correspond to standard error (*n* = 3). **d)** Single turnover discharge of Cy3b-labelled Ub from UBE2A (5 μM) to lysine in the presence and absence of UBR4_xtal_ (5 μM). Gels were visualized by in-gel fluorescence (left) and by Coomassie staining (right). **e)** As above but for UBE2B. Both experiments with UBR4 at 5 μM were carried out once. Source data

To discover whether UBR4_4730–5183_ uses an allosteric mechanism, we tested activity with two UBE2A mutants. Residue Asn80 in UBE2A is part of a highly conserved His–Pro–Asn motif and has an essential role in thioester activation and/or transition state stabilization, but is typically dispensable for cysteine-dependent E3s^8,13,33–37^. On the other hand, residue Ser120 in UBE2A (aspartate 117 in UBE2D1 to UBE2D4) facilitates E2 transfer to lysine and is therefore likely to be a distinct requirement for allosteric E3s (Extended Data Fig. 2a)^38,39^. The necessity for these residues was assessed by quantitative gel-based autoubiquitination assays using fluorescently labeled Ub. To ensure potential perturbation to Ub activation and E1–E2 activity were decoupled, assays were carried out under single turnover E2∼Ub discharge conditions^14^. We found that neither the Asn80Ser nor the Ser120Ala mutant could support UBR4 autoubiquitination activity (Extended Data Fig. 2b,c). Taken together, these data are consistent with the E3 module residing within UBR4 residues 4,730–5,183 operating via an allosteric mechanism.

**Fig. 2:**
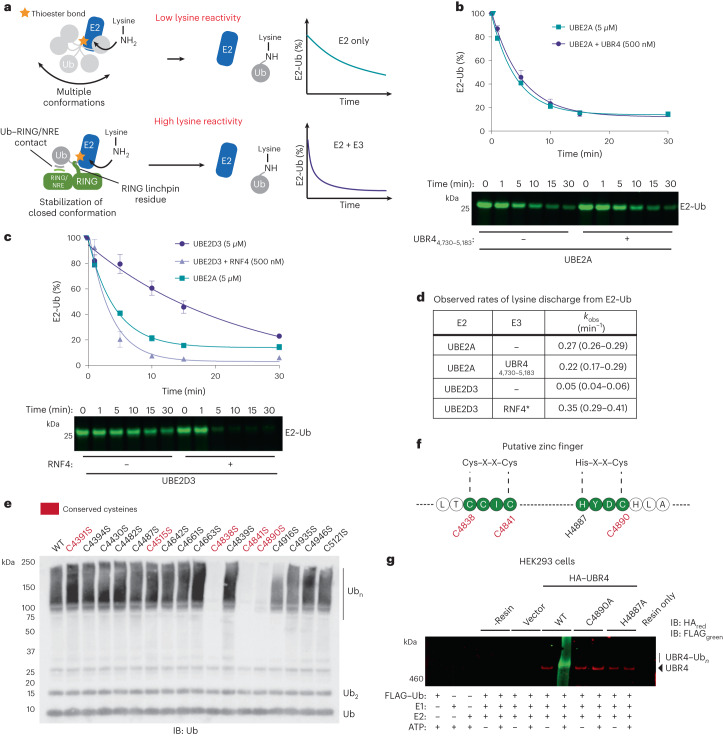
The E3 module in UBR4 demonstrates attenuated E2∼Ub activation and is zinc ion binding. **a**, Prototypical RING E3s stabilize a closed E2∼Ub conformation demonstrating enhanced lysine reactivity. Two key interactions are typically associated with strong enhancement of lysine reactivity. **b**, Single turnover discharge of Ub from UBE2A (5 μM) to lysine (10 mM) in the presence and absence of UBR4_4730–5183_ (500 nM) (activation was neither observed at a higher (5 μM) UBR4 concentration; Extended Data Fig. 2d). **c**, Single turnover discharge of Ub from UBE2D3 to lysine (10 mM) in the absence or presence of an RNF4 variant (500 nM), engineered to be constitutively active^40^. For reference, the UBE2A curve for E3-independent lysine discharge from **b** is overlaid. The means from independent experiments are plotted and bars correspond to the standard error (*n* = 3). **d**, Observed rates, determined from the experiments presented in **b** and **c**, using the half-life equation (*k*_obs_ = ln2/*t*_1/2_). Error bars correspond to the 95% confidence intervals obtained from fitting the data to a single exponential function. **e**, The 17 cysteines within UBR4_long_ were systematically mutated to alanine and autoubiquitination activity was assessed by anti-Ub immunoblot (IB). **f**, Conserved cysteine residues required for robust autoubiquitination activity form a sequence motif consistent with a zinc-ion-binding C2HC zinc finger. **g**, WT and the corresponding HA-tagged UBR4 mutants were transiently overexpressed in HEK293 cells and immunoprecipitated against HA–sepharose resin. Following incubation with ubiquitination components, near-infrared imaging of the membranes was performed. For the UBR4 and FLAG channels anti-rabbit IRDye 680RD (red) and anti-mouse IRDye 800CW (green) were used, respectively. Experiments **e** and **g** were performed twice with similar results. Source data

Thioester activation is achieved by the formation of a closed E2∼Ub conformation, which is stabilized by allosteric E3s^8–10^ (Fig. 2a). To test for this characteristic we used free lysine as a model substrate^13^. However, UBR4_4730–5183_ did not discernibly stimulate Ub discharge from UBE2A or UBE2B, suggesting it stabilizes the closed E2∼Ub conformation to a lesser extent than prototypical RING E3s (Fig. 2b and Extended Data Fig. 2d,e)^8–10^. We next asked if UBE2A possesses intrinsically high lysine aminolysis activity, which would reconcile the lack of robust stabilization. We determined the observed rate of UBE2A discharge to lysine and found it was at least sixfold higher than the prototypical E2 UBE2D3 (Fig. 2c,d). Strikingly, the observed rate of intrinsic UBE2A discharge was comparable to Ub discharge from UBE2D3 when partnered with a constitutively active variant of the prototypical RING E3 RNF4 (Fig. 2c,d)^40^. Thus, attenuated thioester activation might be characteristic of E3s that are cognate for UBE2A/UBE2B, because the intrinsically high aminolysis activity of these E2s can compensate. This high activity might also explain the ability of UBE2A/UBE2B to mediate highly efficient E3-independent proximity-induced protein degradation^41^.

### UBR4 E3 activity is dependent on a zinc finger

**Extended Data Fig. 3:**
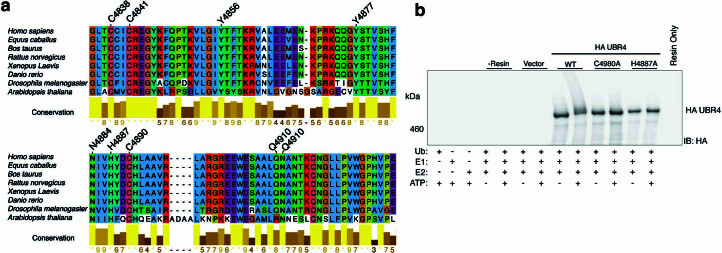
HemiRING sequence alignment and autoubiquitination assay for full-length UBR4 zing finger mutants. **a)** The human UBR4 hemiRING was aligned against selected orthologues. All residues involved in coordination of the single zinc ion are conserved and are labelled in grey. Except for Gln4910, which is an arginine residue in *Arabidopsis*, all residues that form the hydrogen bonding network that replaces the canonical distal zinc coordination site are also conserved and labelled in blue. However, the Gln4910 side chain is solvent exposed suggestive of side chain tolerance at this position. Alignment and figure generation was carried out with Jalview 2.11.2.5 using the Clustal algorithm. **b)** Wild type and the corresponding HA-tagged UBR4 mutants were transiently overexpressed in HEK293 cells and immunoprecipitated against HA-sepharose resin. Washed resin was combined with E1 (500 nM), FLAG-ubiquitin (5 µM), ATP (10 mM) and UBE2A E2 (5 µM). Reactions were incubated at 37 °C for one hour, stopped by the addition of reducing LDS loading buffer and visualized by anti-HA immunoblot. Experiment was performed twice with similar results. Source data

Zinc-finger domains are often found in E3s, and their structural integrity depends on cysteine and histidine ligands^5,14,42^. To test if a cryptic zinc finger existed within UBR4, we mutated the 17 cysteines, 5 of which being conserved, in UBR4_long_ to alanine and assessed the effect on autoubiquitination (Fig. 2e). We found that mutation of three conserved cysteines (Cys4838A, Cys4841A and Cys4890A) abolished activity (Extended Data Fig. 3a). The positions of Cys4838 and Cys4841 correspond to a Cys–X–X–Cys motif, characteristic of a zinc finger^5^. However, four residues typically coordinate the zinc ion, but the conserved histidine residue H4887 at the −3 position relative to Cys4890 (His–X–X–Cys), could complete the ligand network (Fig. 2f and Extended Data Fig. 3a). We reasoned this cryptic C2HC zinc finger might function as an E2 docking site. Further consistent with this, full-length UBR4 Cys4890Ala and His4877Ala mutants lacked autoubiquitination activity (Fig. 2g and Extended Data Fig. 3b).

### The hemiRING–UZI is a distinct E3 module within UBR4

**Fig. 3:**
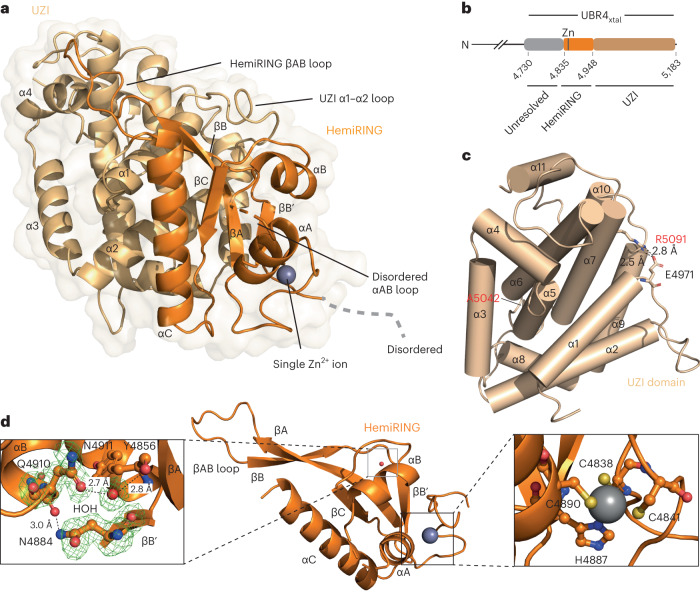
Crystal structure of core zinc-finger UBR4 E3 module. **a**, Surface and cartoon representation of the hemiRING-UZI domain structure refined to 1.8 Å. The hemiRING subdomain is colored in orange whereas the UZI domain is colored in wheat. N-terminal residues 4,730–4,832 and 4,897–4,901, which connect helices αA and αB, were unresolved in the crystal structure. **b**, Domain architecture of hemiRING-UZI subdomain. Color as for **a**, but a region in the crystallization construct (UBR4_xtal_) that also remains unmodeled is highlighted in gray. **c**, The UZI domain comprises a bundle of 11 α-helices. Residues mutated in patients with episodic ataxia are depicted in ball and stick. **d**, Close-up of the hemiRING structure. Left: inset depicts the water-mediated hydrogen bonding network that substitutes for the coordination of a second zinc ion. Green mesh corresponds to a *F*_obs_ − *F*_calc_ difference map for Asn4884, Gln4910 and water molecule 199, calculated with Phenix and contoured at 3.0*σ*. Right: inset depicts the C2HC coordination network for the single zinc ion.

**Extended Data Fig. 4:**
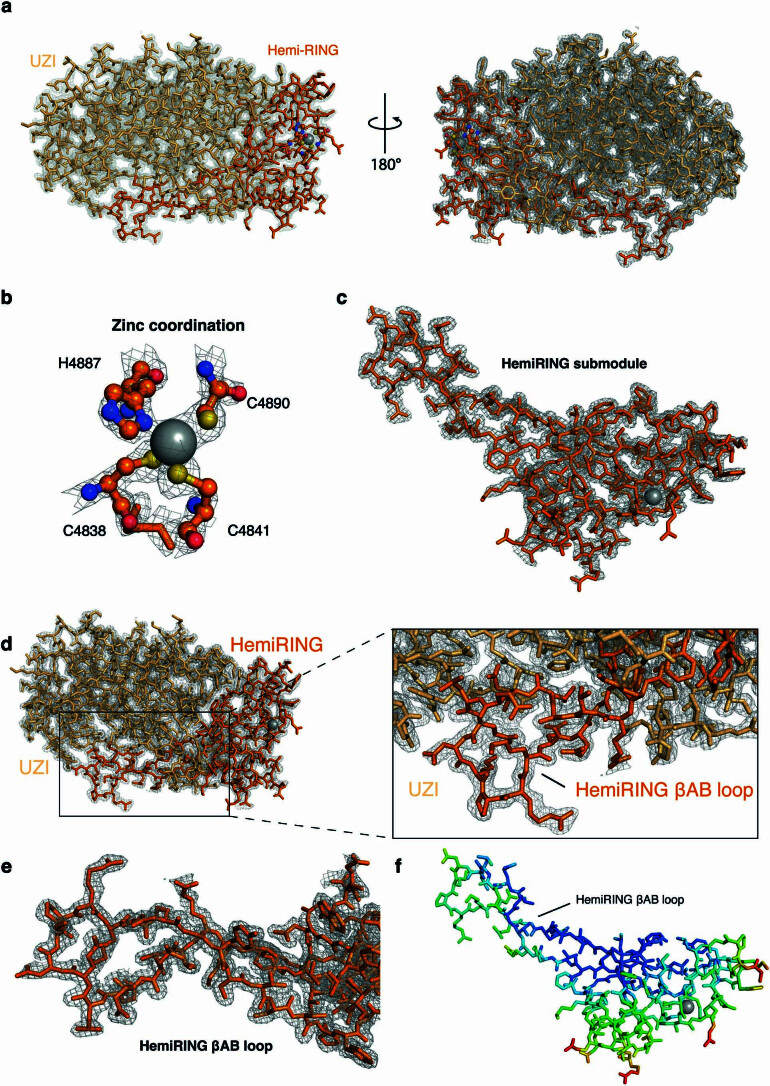
Representative views of the crystallographic model of the UBR4 E3 module. **a)** A single molecule, corresponding to residues 4831-5183, was found in the asymmetric unit. The model is represented in stick where the hemiRING submodule is coloured orange and the UBR Zinc finger-Interacting (UZI) subdomain is coloured wheat. **b)** Close up of the coordination network towards the single Zn^2+^ ion in the hemiRING submodule. Mutation of these residues ablates E3 ligase activity. **c)** Isolated view of the hemiRING submodule. Inserted into the hemiRING is a pronounced extension made up of the βAB loop. **d)** Interaction between the hemiRING βAB loop and the UZI subdomain, the latter appearing to be integral to the stability of the βAB loop. **e)** Close up isolated view of the hemiRING βAB loop. For panels a-e the mesh corresponds to a 2|*F*_*obs*_|−|*F*_*calc*_| electron density map contoured at 1.0 σ. **f)** HemiRING sub-module colored by B-factor (blue is lowest and red is highest).

**Extended Data Fig. 5:**
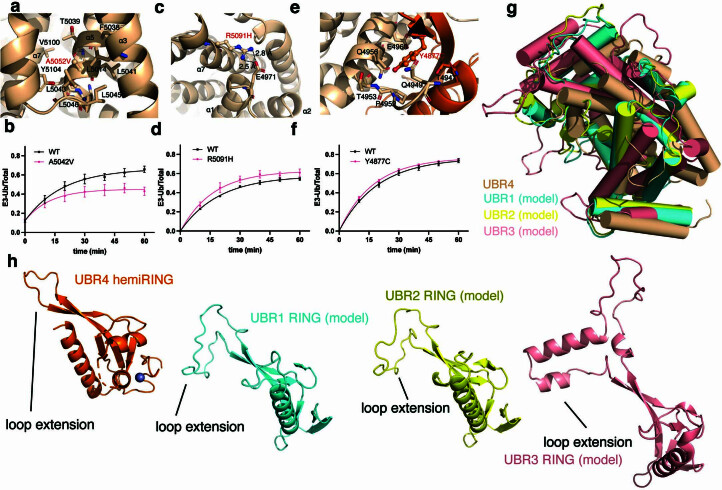
Episodic ataxia (EA) patient mutations within the hemiRING-UZI module, structural superposition of UBR1-4 UZI subdomains and AlphaFold models of RING domains from UBR1, UBR2 and UBR3. **a)** Structural context residue Ala5042. An Ala5042Val mutation is associated with EA patients. **b)** Introduction of the Ala5042Val mutation impaired UBR4 autoubiquitination. **c)** Structural context of Arg5091 which is mutated to histidine in EA patients. **d)** The Arg5091His mutant had slightly reduced autoubiquitination activity. **e)** Structural context of Tyr4877Cys which is mutated to cysteine in EA patients. **f)** The Tyr4877Cys mutation demonstrated a modest defect in UBR4 autoubiquitination. For **b**, **d** and **f** the mean from independent experiments is plotted and bars correspond to standard error (*n* = 3). **g)** Structural superposition of AlphaFold models of UZI subdomains predicted to exist within *Homo sapiens* UBR1-3 with our experimentally determined structure of the UZI subdomain from UBR4. **h)** AlphaFold models of the canonical RING domains from UBR1-3 indicates that like the UBR4 hemiRING, they contain pronounced insertions within the RING domain that also interact with their respective UZI domain that might also result in stabilization of the former. Source data

To gain further insights into the E3 module, we solved the structure of UBR4_4730–5183_. Crystals of UBR4_4730–5183_ (referred to hereon as UBR4_xtal_) were obtained after sparse matrix screening and condition optimization. Diffraction data were collected, and a 1.8 Å structure was solved using the anomalous signal from a single zinc ion present in the protein (Fig. 3a,b, Table 1 and Extended Data Fig. 4). Residues 4,730–4,830 of UBR4_xtal_ were not resolved, so a model could only be built for residues 4,831–5,183 (Fig. 3b). Of note, a designed construct approximating this region (UBR4_4828–5183_) was inactive in our autoubiquitination assay (Fig. 1e). Our structure is composed of two apparent subdomains that constitute a larger fold. The N-terminal subdomain comprises residues 4,835–4,948 and is followed by the second subdomain containing 11 α-helices that run to the native UBR4 C-terminus (Fig. 3c). No established folds with homology to this region were identified with the DALI comparison server, with the most similar found in rhamnosidase B (DALI *Z*-score 6.5)^43^. As such, we consider this subdomain a novel fold and refer to it as the UBR zinc-finger interacting (UZI) region. Interestingly, two mutations found in patients with episodic ataxia, Ala5042Val and Arg5091His, reside within the UZI subdomain (Extended Data Fig. 5a,c)^44,45^. Ala5042 is located within helix α5 and packs against helices α3 and α6, whereas Arg5091 is in helix α7 and forms a strong salt bridge with α1–2 loop residue Glu4971 (Fig. 3c). We found that the Ala5042Val mutant exhibited impaired autoubiquitination activity, whereas only modest impairment was observed with Arg5091His (Extended Data Fig. 5b,d).

**Table 1:** Data collection and refinement statistics for UBR4_xtal_ and the UBR4_xtal_–UBE2A complex

	UBR4_xtal_	UBR4_xtal_–UBE2A complex
**Data collection**		
Space group	I 2 2 2	P 43 21 2
Cell dimensions		
*a*, *b*, *c* (Å)	67.131, 84.644, 148.13	89.1569, 89.1569, 263.306
α, β, γ (°)	90, 90, 90	90, 90, 90
Resolution (Å)	74.07 - 1.8 (1.864 - 1.8)	65.83 - 3.2 (3.315 - 3.2)
*R*_merge_	0.02395 (0.2907)	0.2103 (1.251)
CC_1/2_	0.999 (0.872)	0.997 (0.934)
*I*/σ*I*	23.84 (2.56)	8.04 (1.35)
Completeness (%)	91.33 (91.67)	99.62 (99.50)
Redundancy	2.0 (2.0)	12.5 (13.1)
**Refinement**		
Resolution (Å)	74.07 - 1.8	65.83 - 3.2
No. reflections	71,900 (6,996)	229,999 (23,721)
*R*_work_/*R*_free_	0.1981/0.2220	0.2282/0.2623
No. atoms		
Protein	2,723	5,186
Ligand/ion	9	1
Water	408	0
*B* factors		
Protein	26.84	82.05
Ligand/ion	38.66	63.83
Water	36.44	N/A
RMSDs		
Bond lengths (Å)	0.003	0.002
Bond angles (°)	0.56	0.45

Values in parentheses correspond to the highest-resolution shell.

Data were phased by single anomalous diffraction or molecular replacement, respectively. Both datasets were collected from single cystals. *I*, observed intensity; σ, standard deviation.

**Fig. 4:**
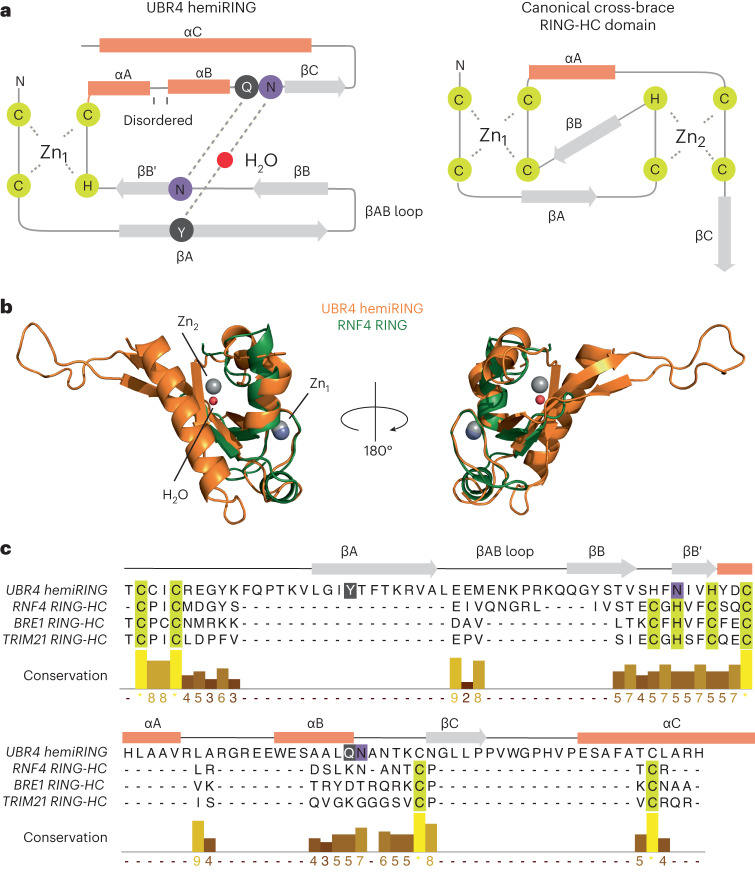
Comparison of the hemiRING with canonical RING domains. **a**, Schematic representations of the UBR4 hemiRING and a canonical RING-HC domain. Orange rectangles represent α-helices, and gray arrows represent β-strands. Residues observed to hydrogen bond through their backbone are in gray circles, whereas those that interact through their side chain are in purple circles. **b**, Structural superposition of the UBR4 hemiRING (orange) onto the RNF4 RING domain (green; PDB 4PPE)^46^. The hemiRING zinc ion is a dark-gray sphere, and the central water molecule is a red sphere. The two zinc ions in RNF4 are light-gray spheres. **c**, Sequence alignment of the *Homo sapiens* (*Hs*) UBR4 hemiRING submodule with cross-brace RING domains from *Hs* RNF4, BRE1 and TRIM21. Cysteine and histidine residues that coordinate a zinc ion are highlighted in yellow and residues proximal to the structural water molecule that hydrogen bond via their backbone or side chain are highlighted in gray or purple, respectively.

Our experimental structure confirms the structural role of conserved residues Cys4838, Cys4841, Cys4890 and His4887 as zinc coordinating ligands (Fig. 3d and Extended Data Fig. 4b). Although not evident from primary sequence, the zinc finger has partial structural homology with canonical RING domains (DALI *Z*-score range 2.0–5.8; Fig. 4a,b)^46^. The protein fold immediately proximal to zinc ion 1 (referred to hereon as the proximal Zn^2+^ site), which engages the E2 enzyme in canonical RING domains, is conserved, together with the tailing helix and core β-sheet (Fig. 4a,b)^47^. However, residues essential for coordinating the second zinc ion (referred to hereon as the distal Zn^2+^ site) are conspicuously absent. Substituting for the second zinc ion are four residues that form hydrogen bonds, and are probably required for stabilizing the cross-brace architecture of the zinc finger subdomain (Figs. 3d and 4a). Tyr4856 forms a central water-mediated hydrogen bond, via its backbone carbonyl, to the side chain of Asn4911, and these residues are located within strand βA and the end of helix αB, respectively. Following the His–X–X–Cys motif, that completes the coordination of the proximal Zn^2+^ site, the side chain of Asn4884, located within strand βB’, hydrogen bonds with the backbone carbonyl of Gln4910 (Figs. 3d and 4a). Except for Gln4910, whose side chain is solvent-exposed and interacts via its backbone carbonyl, these residues are conserved across UBR4 orthologs (Extended Data Fig. 3a). The hybrid U-box/RING nature of the UBR4 zinc finger is reminiscent of the SP-RING domain found in SUMO E3s of the Siz/PIAS family^48^. However, the SP-RING maintains the distal Zn^2+^ site, rather than the proximal site, and lacks the extended βAB sheet. Thus, to our knowledge, the UBR4 zinc finger has unprecedented RING-related architecture, and we refer to it as the hemiRING.

**Extended Data Fig. 6:**
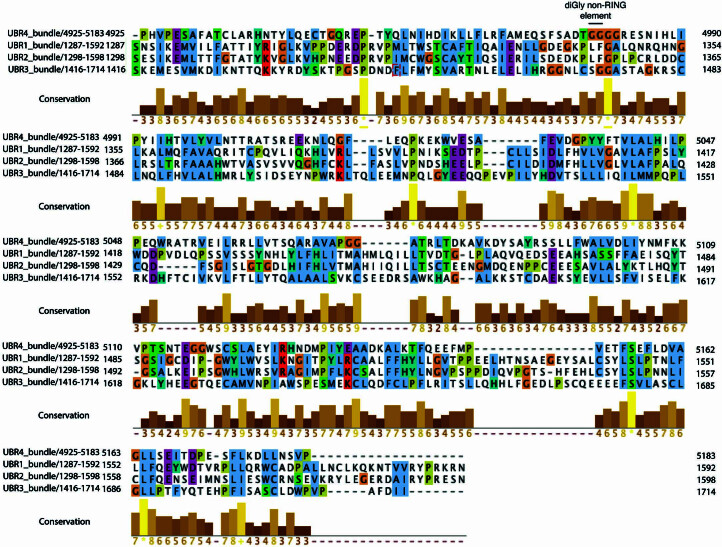
Sequence alignment of UBR1-4 UZI subdomains. Sequences were aligned with Clustal using Jalview 2.11.2.5.

An extended β-sheet composed of the hemiRING βA and βB strands packs against a C-terminal hemiRING helix, and α2 of the UZI subdomain. The βA and βB strands are connected via a lengthy loop, which makes further contacts with helices α1 and α4 of the UZI subdomain (Fig. 4a–c and Extended Data Fig. 4). Located within the following βB′ strand is Tyr4877, which makes hydrophobic contacts with the UZI (Extended Data Fig. 5e) and is the site of another episodic ataxia mutation (Tyr4877Cys). However, despite Tyr4877 being highly conserved, introducing the Tyr4877Cys mutation had no discernible effect on autoubiquitination (Extended Data Figs. 3a and 5f). Interestingly, the RING domain in yeast UBR1 has an analogous βAB loop that is even longer and similarly sandwiched by a helical-rich fold known as the cap helical domain^4^. We extended the fold comparison of the UZI subdomain beyond experimentally determined structures using the DALI AF-DB comparison tool^49^. Strikingly, the top matches were the human UBR E3s, which contain a RING domain (UBR1, UBR2 and UBR3; *Z*-score 6.6–8.0; root mean square deviation (RMSD) 3.8–4.2 Å). The 11 helices comprising the UZI domain are also conserved in these E3s (Extended Data Figs. 5g and 6). Their RING domains are similarly predicted to have large insertions (Extended Data Fig. 5h). Hence, UBR1 to UBR3 might possess a similar ‘RING-UZI’ module. Attempts to obtain soluble expression of the UBR4 hemiRING lacking the UZI subdomain were unsuccessful, consistent with these folds being structurally interdependent.

### The hemiRING is an E2-binding module

**Fig. 5:**
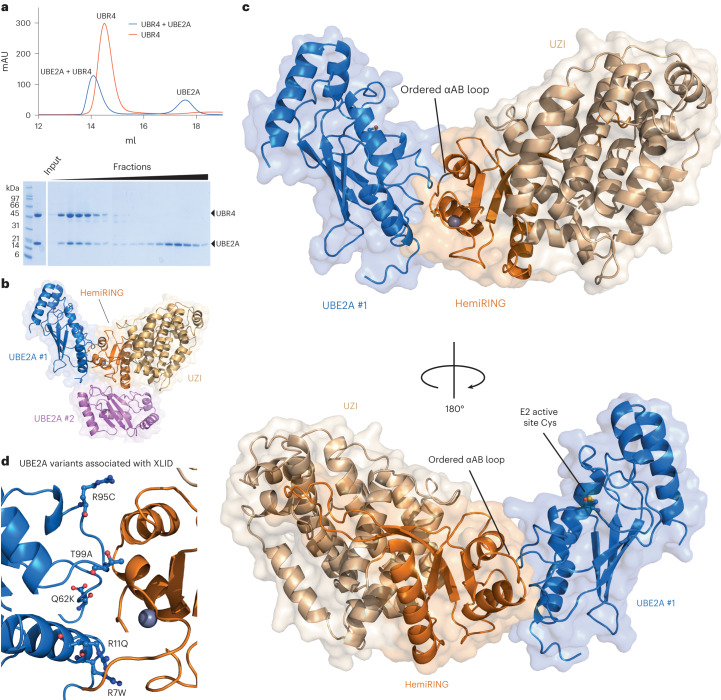
Crystal structure of UBR4 hemiRING E3 in complex with E2 conjugating enzyme UBE2A. **a**, UBR4_xtal_ and UBE2A form a stable complex when analyzed by size-exclusion chromatography using a Superdex 200 10/300 GL (Cytiva Life Sciences). Experiment was performed once. **b**, Asymmetric unit of the 3.2 Å crystallographic model for UBR4 hemiRING and UZI subdomains in complex with UBE2A. The structure is in cartoon and transparent surface representation. Two UBE2A molecules are present in the asymmetric unit. Molecule UBE2A #1 is blue and binds proximal to the zinc-binding region of the UBR4 hemiRING. The second UBE2A molecule, UBE2A #2, is in violet. **c**, Enlarged views of the complex between UBR4 and UBE2A #1. A Cys88Lys UBE2A mutant was used for crystallization, and this residue has been mutated to a cysteine in silico. The top and bottom projections represent a 180° rotation about the vertical. **d**, XLID patient mutations cluster at the UBE2A–hemiRING interface. Residues are depicted in ball in stick and annotated with the pathogenic variant.

**Extended Data Fig. 7:**
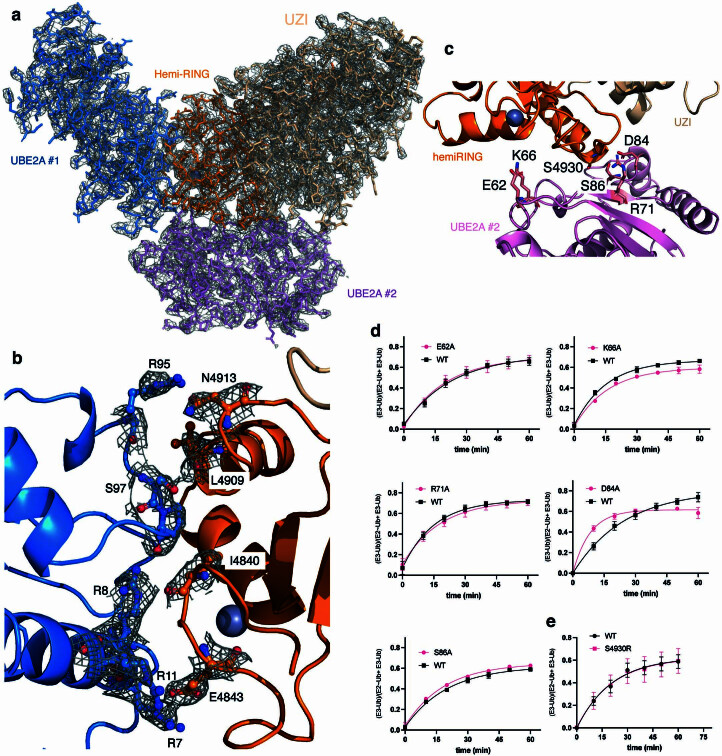
Representative views of the crystallographic model of the UBE2A-UBR4 complex. **a)** The complete asymmetric unit in stick format is overlaid with the 2|*F*_*o*_|*-*|*F*_*c*_| map**. b)** The UBE2A-hemiRING interface. Key residues are depicted in ball and stick and their corresponding 2|*F*_*o*_|*-*|*F*_*c*_| map is overlaid. Maps contoured at 1.0 σ**. c)** The asymmetric unit presented with two UBE2A molecules. UBE2A residues Glu62, Lys66, Arg71, Asp84 and Ser86 were identified as being of potential importance for maintaining the interaction with UBE2A #2 (pink). UBR4 Ser4330 packed closely against UBE2A and it was assumed that mutation to arginine would abolish the UBR4-UBE2A #2 interaction. **d)** The functional relevance of the observed UBR4-UBE2A #2 interaction on UBR4 autoubiquitination was assessed by testing UBE2A alanine mutants of the identified residues. **e)** To exclude the possibility that the UBE2A mutations had a general effect on UBE2A function we tested the steric mutation in UBR4 (Ser4930Arg). For panels **d** and **e** the mean from independent experiments is plotted and bars correspond to standard error (*n* = 3). Source data

UBE2A variants cause UBE2A deficiency syndrome, an X-linked intellectual deficiency condition known as Nascimento type (MIM 300860) that is characterized by speech impairment, dysmorphic facial features and genital abnormalities^50–52^. Neither the atomic basis for the recognition of UBE2A by a cognate E3, nor the pathogenic origin of these variants are known. When analyzed by size-exclusion chromatography, a UBR4_xtal_ and UBE2A mixture eluted as a complex, indicative of a stable interaction (Fig. 5a). To determine the structure of this complex, we obtained crystals and collected diffraction data to 3.2 Å. A structure was solved by molecular replacement using the apo structure obtained from UBR4_xtal_, and a UBE2A crystal structure (Fig. 5b,c, Table 1 and Extended Data Fig. 7)^53^. As for the apo structure, the N-terminal ∼100 residues in the UBR4_xtal_ construct were unresolved. The UBR4 molecule was equivalent to the apo structure (RMSD of 0.98 Å), except for the hemiRING loop connecting αA and αB, gaining structural order, suggestive of E2 binding having a stabilizing effect (Fig. 5c).

**Extended Data Fig. 8:**
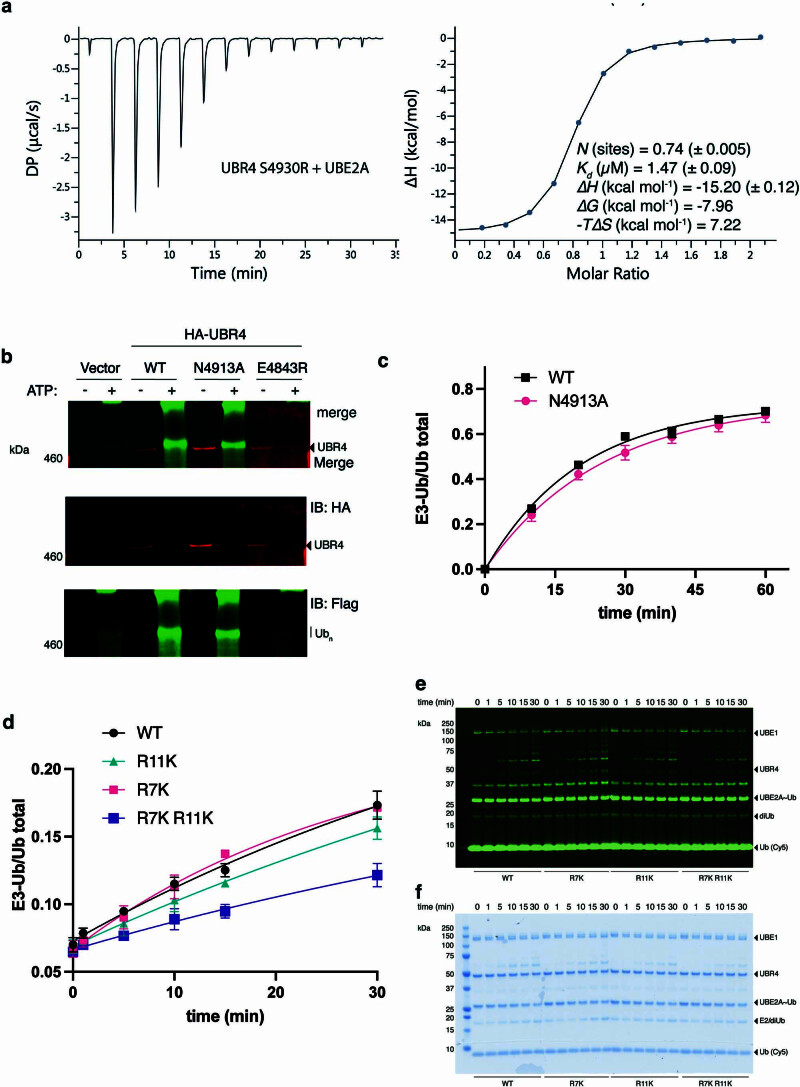
Additional validation experiments for the crystal structure of the UBR4-UBE2A complex. **a)** Thermodynamic parameters for UBE2A binding to UBR4_xtal_ Ser4930Arg were comparable to wild type UBR4_xtal_ (Fig. 6a), demonstrating that the affinity of the second E2 interaction is negligible. Presented errors are from fitting. This experiment was carried out once. **b)** Wild type and the corresponding HA-tagged UBR4 mutants were transiently overexpressed in HEK293 cells and immunoprecipitated against HA-sepharose resin. The experiment was carried out as for Fig. 2g and Extended Data Fig. 3b, but near-infra red imaging of the membranes was achieved with a Chemidoc imager (Biorad), using instrument settings for IRDye 680RD and IRDye 800CW. **c)** Introducing a Glu4843Arg mutation in the UBR4 hemiRING abolished detectable autoubiquitination of full-length UBR4. Asn4913, which also resides at the linchpin position found in RING E3 prototypes, was mutated to alanine in UBR4_xtal_ and autoubiquitination activity was assessed. The assay was carried out under single turnover E2∼Ub discharge conditions. The mean from independent experiments is plotted and bars correspond to standard error (*n* = 3). **d)** Lysine residues present in UBE2D1-3 were introduced in place of UBE2A Arg7 and Arg11. Only a modest reduction in activity is observed upon the mutations suggesting that these residues are not the only specificity determinants for hemiRING binding. The mean from independent experiments is plotted and bars correspond to standard error (*n* = 3). **e)** Raw representative data measuring in-gel fluorescence from Cy3-labeled Ub. **f)** Coomassie stain of the same gel to confirm comparable loading. Source data

Two UBE2A molecules are present in the asymmetric unit, where the first E2 molecule (UBE2A #1) binds the hemiRING proximal to the zinc coordinating fold, as observed with RING domains (Fig. 5c)^47^. Several X-linked intellectual disability (XLID) patient mutations within UBE2A cluster at this interface (Fig. 5d)^52,54,55^. We could not establish any mechanistic role for E2 molecule 2 (UBE2A #2) because mutation of E2, or E3, residues at the interface had no apparent effect on UBR4 autoubiquitination activity or affinity (Fig. 5b and Extended Data Figs. 7c–e and 8a). Thus, the UBE2A #2 interaction is probably a consequence of crystallographic packing.

### E3 activity depends on the E2–hemiRING interaction

**Fig. 6:**
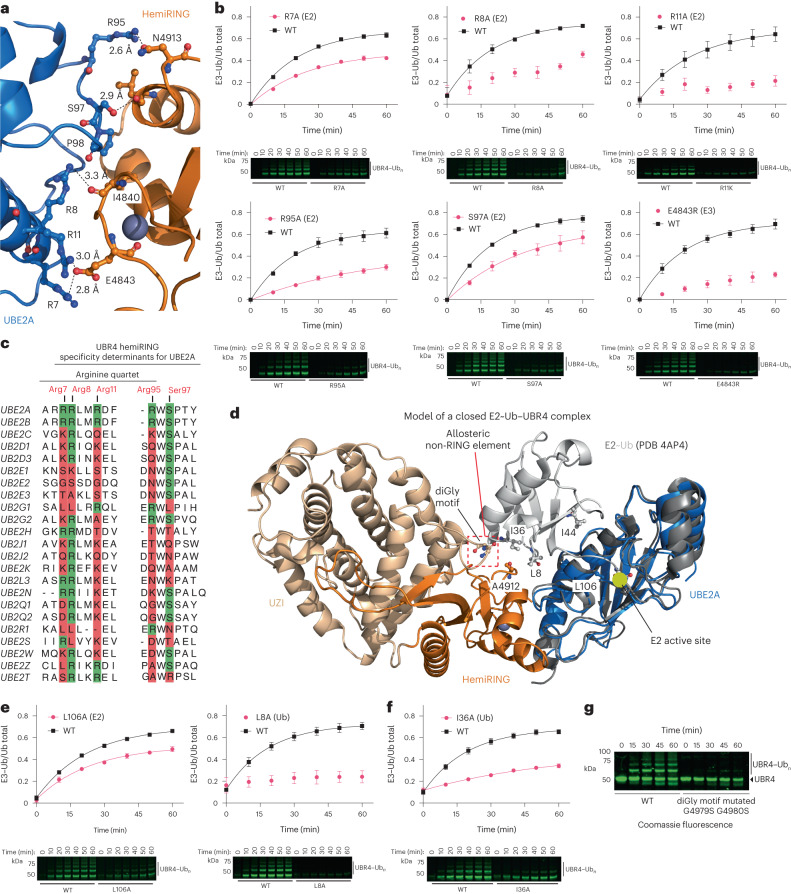
The UBE2A–UBR4 hemiRING interface and the requirement for a closed E2∼Ub conformation. **a**, Close-up of the interface between UBE2A and the hemiRING. Four UBE2A arginine residues poorly conserved across mammalian E2s form key interactions with the hemiRING. UBE2A is in blue cartoon and key residues are in ball and stick. UBR4 hemiRING residues are in orange and key residues are in ball and stick. **b**, Mutational analysis of crystallographic interfacial UBE2A–hemiRING residues by in gel fluorescent autoubiquitination assay. Assays were carried out under single turnover E2∼Ub discharge conditions. **c**, Sequence alignment performed with Jalview 2.11.2.5 using the Clustal algorithm for 22 mammalian E2 conjugating enzymes. Only UBE2A and UBE2B contain interface residues we experimentally found essential for optimal activity (Arg7, Arg8, Arg 11 and Ser97). **d**, Model of a ternary closed E2∼Ub–UBR4 complex obtained by superposition of UBE2D1∼Ub (PDB ID: 4AP4) onto the UBE2A E2 molecule (blue) in our experimental UBE2A–UBR4 structure. Ub residues essential for E3s that stabilize a canonical closed conformation are in gray ball and stick notation and are labeled. **e**, Mutation of Leu106 and Leu8 disrupts E3-dependent and E3-independent lysine discharge because they make contacts at the E2–Ub interface within the closed conformation. However, Leu106 in UBE2A is not solvent-exposed and its mutation to alanine only partially impaired autoubiquitination. Assays were carried out under single turnover E2∼Ub discharge conditions. **f**, Ile36 is not at the E2–Ub interface, and its mutation impairs activity with prototypical RING E3s that stabilize a closed E2∼Ub conformation because they form hydrophobic contacts with this residue. Thus, its requirement should be a diagnostic for allosteric E3 activity. For **b**, **e** and **f** the means from independent experiments are plotted and bars correspond to the standard error (*n* = 3). **g**, Multiple turnover autoubiquitination assay comparing WT UBR4_xtal_ with the UBR4_xtal_ Gly4979Ser Gly4980Ser double mutant. Coomassie fluorescence was measured using the 715/30 filter in a ChemiDoc system (Bio-Rad). The assay was carried out twice with similar results. Source data

Although ancillary E3 elements that engage the backside of RAD6B (yeast ortholog of UBE2B) have been structurally delineated, the basis for selective recognition of UBE2A/UBE2B by a core E3 module is unclear^56^. The most notable interactions arise from UBE2A residues Arg7, Arg8, Arg11, Arg95 and Ser97. Arg7, Arg8 and Arg11 reside in the UBE2A N-terminal α-helix, whereas Ser97 and Arg95 are in loop 2. The guanidino groups of Arg7 and Arg11 hydrogen bond with the side chain of UBR4 Glu4843 (Fig. 6a). Mutation of either of these residues to alanine, or mutating Glu4843 to arginine, severely impaired UBR4 autoubiquitination (Fig. 6b). Furthermore, autoubiquitination of full-length UBR4 containing the Glu4843Arg mutation was below the level of detection (Extended Data Fig. 8b). These defects are particularly insightful as Arg7Trp and Arg11Gln mutations are found in patients with XLID and, based on our findings, would impair UBE2A engagement by the UBR4 hemiRING and, in turn, compromise substrate ubiquitination^54,55^. On the premise that UBR4 has neuronal functions^19,21,22^, it is tempting to speculate that this observation underlies XLID pathogenicity.

UBE2A Ser97 side chain also forms an important anchor point by hydrogen bonding to the backbone carbonyl of UBR4 Ile4840, the side chain of which also makes hydrophobic contacts with UBE2A Pro98 (Fig. 6a,b). The only other interfacial contact of note is between UBE2A Arg95 and UBR4 Asn4913, where an Arg95Ala mutation substantially impaired autoubiquitination (Fig. 6a,b). However, the reciprocal mutation of Asn4913 had no effect (Extended Data Fig. 8b,c), suggesting Arg95 forms an unobserved interaction, such as a water-mediated hydrogen bond with the UBR4 backbone. Arg95 is also mutated in patients with XLID (Arg95Cys), which is likely to compromise E3 activity^52^.

With archetypal UBE2D1 to UBE2D4 isoforms, glutamine (Gln92) is found in place of Arg95, whose backbone carbonyl interacts with the electrostatic linchpin residue in canonical RING domains^8–10^. Structural and sequence analysis maps the equivalent site of this linchpin residue to Asn4913 in the hemiRING. Although asparagine is a functional linchpin in the cullin-associated RING protein RBX1 (ref. ^57^), this residue is unlikely to be functional in UBR4 as previous measurements showed its mutation did not affect full-length UBR4 or UBR4_xtal_ activity (Extended Data Fig. 8b,c).

To explore the minimum specificity determinants at the E2–UBR4 interface, we carried out a sequence alignment of 22 E2s (Fig. 6c). A characteristic of UBE2A/UBE2B is a quartet of arginine residues (Arg7, Arg8, Arg11 and Arg95) and Ser97. Individual conservation of these residues varies from low to moderate, but the α-helix 1 trio of arginine residues (Arg7, Arg8 and Arg11) is unique to UBE2A/UBE2B, with the nonfunctional UBE2D1 to UBE2D4 isoforms differing only by the presence of isoelectronic lysine residues in place of Arg7 and Arg11. Therefore, to establish if the arginine trio is the minimum specificity determinant, we introduced the lysine residues found in UBE2D1 to UBE2D4 and tested UBR4 autoubiquitination activity. Although UBE2A Arg11Lys had lower activity, which was further reduced with the double mutant, the basal activity implied that additional elements (for example, Arg95, which is glutamine in UBE2D3) are required for the exquisite specificity between UBR4 and UBE2A/UBE2B (Extended Data Fig. 8d–f).

### UBR4 activity involves a closed E2∼Ub conformation

**Extended Data Fig. 9:**
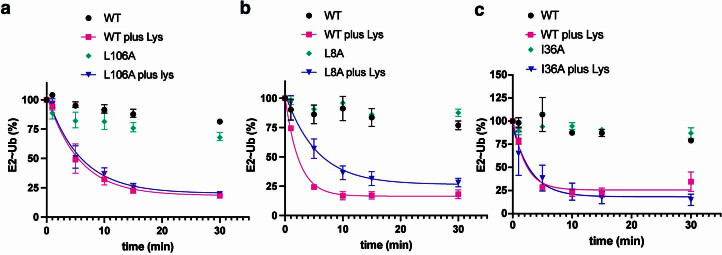
Effect of UBE2A and Ub residues on E3-independent discharge to free lysine deemed important for the formation of a closed E2∼Ub conformation. **a)** UBE2A WT discharges to free lysine (10 mM) with an efficiency comparable to Leu106Ala, indicating this mutation does not impart a general E3-independent defect on UBE2A. **b)** UBE2A Leu8Ala has impaired lysine discharge activity indicative of a general defect in E2 activity. **c)** UBE2A Ile36Ala has no discernable effect on E3-independent lysine discharge, consistent with it being distal to the E2 active site and the E2-Ub interface that exists within a closed E2∼Ub conformation. For panels **a**, **b** and **c** the means from independent experiments are plotted and bars correspond to standard error (*n* = 3). Source data

UBE2A can sample the closed conformation in the absence of an E3 (ref. ^53^), and by modeling a ternary E2∼Ub–UBR4 complex, we ascertained that this was sterically compatible with our E2–hemiRING–UZI structure (Fig. 6d). To test the importance of a closed UBE2A∼Ub conformation, albeit without assessment of whether UBR4 stabilizes it, we mutated residues that impede its formation and tested activity. With the UBE2D1 to UBE2D4 isoforms, Leu104 in the E2 crossover helix makes important hydrophobic contacts with the Ile44 patch of Ub, and its mutation abolishes allosteric E3 activity^8–10,58^. However, the equivalent residue in UBE2A is Leu106, which is positioned toward the core of the E2 fold, resulting in the equivalent exposed surface being hydrophilic^53^. Consistently, a Leu106Ala mutation only modestly impaired autoubiquitination activity (Fig. 6e). No detectable defect was observed in E3-independent lysine discharge, suggesting that the anomalous Leu106 site on UBE2A is a distinct requirement for optimal UBR4-mediated autoubiquitination (Extended Data Fig. 9a). Another residue important for activity with RING prototypes studied so far is Ub Leu8, which packs against hydrophobic regions on the E2 and the E3 (refs. ^8,10^). A Leu8Ala mutant abolished autoubiquitination activity. However, E3-independent discharge to lysine was also impaired, indicating a general defect in E2 activity (Fig. 6e and Extended Data Fig. 9b).

In light of the inability of UBR4 to stimulate Ub discharge to free lysine, and it being devoid of a linchpin residue, we next tested if it stabilizes the closed UBE2A∼Ub conformation in an attenuated manner. Tempered stabilization is used by some E3s to tune acceptor amino acid or substrate specificity, but is achieved with a suboptimal, rather than absent, linchpin^38,59^. With prototypical RING E3s, the linchpin interaction is complemented by hydrophobic contact with Ub Ile36, either via a second RING protomer (in the case of dimeric RING E3s)^8,10^ or with a non-RING element^60,61^. Interestingly, Ub Ile36 in our model of a ternary complex involving a closed UBE2A∼Ub conformation, is proximal to hydrophobic residues Gly4979 and Gly4980 that reside within a loop in the UZI subdomain. This raised the possibility that the diGly motif is a non-RING element (Fig. 6d). In support of this, a Ub Ile36Ala mutant was defective in autoubiquitination, whereas E3-independent discharge to free lysine was unaffected (Fig. 6f and Extended Data Fig. 9c). Moreover, autoubiquitination was strongly impaired when UBR4 residues Gly4979 and Gly4980 were mutated to hydrophilic serine residues (Fig. 6g). Collectively, we conclude that UBR4 allosterically imparts stabilization of the closed E2∼Ub conformation via hydrophobic contacts between the UZI subdomain and Ub Ile36. The lack of a linchpin results in an attenuated level of stabilization, which is probably balanced by the intrinsically high lysine reactivity of UBE2A/UBE2B.

### An additional UBR4 region contributes to E2 binding

**Extended Data Fig. 10:**
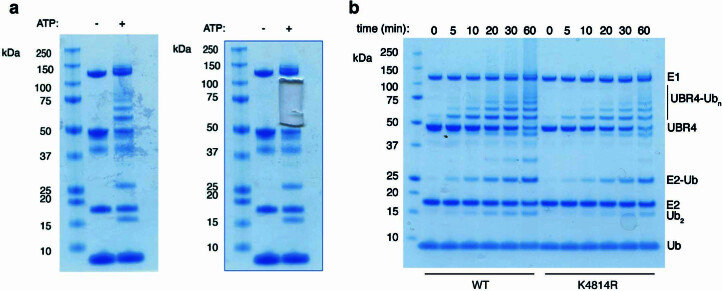
Gel sample selected for analysis by mass spectrometry and multiple turnover autoubiquitination reaction for UBR4_xtal_ K4814R. **a)** An autoubiquitination reaction was carried out with UBR4_xtal_ and the predominant autoubiquitination adducts were excised, dehydrated and resuspended using standard procedures. The left hand gel image is before excision, whereas the right hand image illustrates the precise region excised for analysis by mass spectrometry. The only identified autoubiquitination site was Lys4814. Experiment performed once. **b)** Autoubiquitination is only modestly impaired when Lys4814 is mutated, indicating additional sites. SDS-PAGE was carried out under reducing conditions. Experiment performed once.

We next investigated why a construct that lacked the structurally unresolved N-terminal region of UBR4_xtal_ (UBR4_4828–5130_; Fig. 1e) did not undergo autoubiquitination (Fig. 3b). Initially, we tested whether loss of autoubiquitination sites was the cause. To map sites, we excised the predominant autoubiquitination products from a Coomassie-stained sodium dodecyl sulfate–polyacrylamide gel electrophoresis (SDS–PAGE) gel and analyzed them by data-dependent mass spectrometry (Extended Data Fig. 10a and Source data). Sequence coverage was incomplete, and only a single autoubiquitination site at Lys4814 was mapped (localization probability of 95%), which is located within the unresolved N-terminal region. Multiple isopeptide Ub linkages were also identified, including Lys33, Lys48, Lys63, Lys11 and Lys6 (location probabilities of, 100%, 100%, 96%, 85% and 83%, respectively). However, autoubiquitination of a Lys4814Arg mutant was only modestly impaired, indicating that additional autoubiquitination sites existed (Extended Data Fig. 10b).

**Fig. 7:**
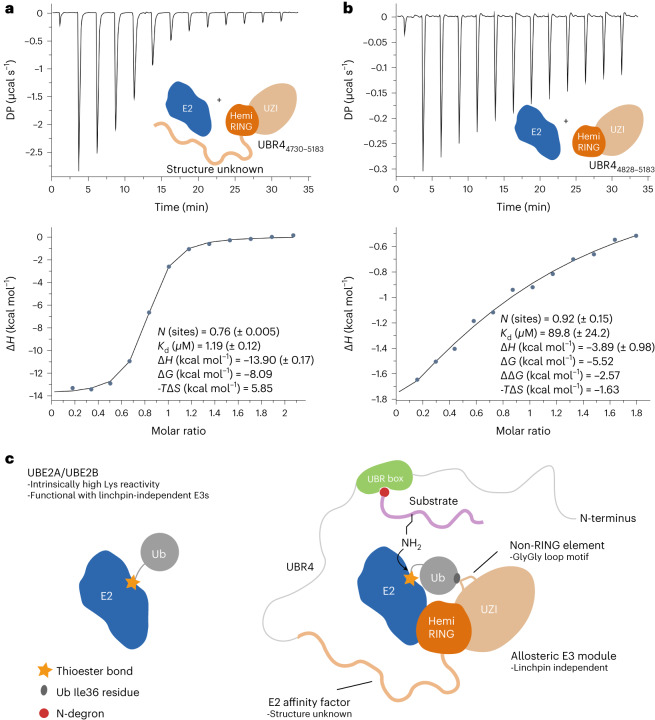
Affinity measurements between UBR4_xtal_ and UBR4_4828–5183_ with UBE2A and a schematic for the UBR4 mechanism. **a**, ITC isotherm for UBE2A binding to construct UBR4_xtal_, which contains the structurally unresolved N-terminal residues (amino acids 4,730–4,832) that are required for autoubiquitination. **b**, ITC isotherm for UBE2A binding to construct UBR4_4828–5183_, which lacks the N-terminal residues. ΔΔ*G* = Δ*G*_UBR4,xtal_ − Δ*G*_UBR4,4828–5183_ indicating the N-terminal region contributes −2.57 kcal mol^−1^ to the free energy of binding. Errors for thermodynamic parameters were obtained from fitting. Duplicate experiments with similar results are presented in Supplementary Information. For **a** and **b**, the top graph represents the raw heats of injection. The bottom panel represents the integrated heats of injection, which were fitted to a single site binding model. **c**, Mechanism and interplay between UBR4 and its cognate E2s, UBE2A/UBE2B. The hemiRING plays an important role in maintaining specificity for UBE2A/UBE2B. UBE2A/UBE2B have relatively high intrinsic lysine reactivity, obviating the need for the robust thioester activation demonstrated by RING E3 prototypes. UBR4 does not have a functional linchpin residue, but the UZI subdomain cooperates with the hemiRING by providing a diglycine motif that serves as a non-RING element (NRE), which promotes the closed conformation by forming hydrophobic contacts with Ub Ile36. Both hemiRING binding and NRE engagement are required for E3 activity. The UBR box binds protein substrates with destabilizing N-degrons and probably positions them in the proximity of the bound and activated E2∼Ub conjugate to enable their ubiquitination.

As we could not formally ascribe the removal of autoubiquitination sites to the inactivity of the construct lacking the N-terminal region, we explored whether it bound noncanonically to UBE2A, as demonstrated by ancillary elements in certain RING E3s^56,62–64^. Initially, we measured the interaction between UBE2A and UBR4_xtal_ by isothermal titration calorimetry (ITC) and established that the free energy of binding (Δ*G*) is −8.09 kcal mol^−1^ (*K*_d_ of 1.19 (±0.12) μM), with an appreciable entropic penalty (−*T*Δ*S* = 5.85 kcal mol^−1^) (Fig. 7a). Strikingly, we found that the construct lacking N-terminal region, UBR4_4828–5130_, had a 75-fold lower affinity (*K*_d_ of 89.8 (±24.2) μM) for UBE2A, revealing that its presence contributes a favorable −2.57 kcal mol^−1^ to the free energy of binding (Δ*G*) (Fig. 7a,b). Consistent with the N-terminal region making contacts with UBE2A, a less favorable enthalpy change was measured for UBR4_4828–5130_ (Δ*H* = −3.89 versus −13.90 kcal mol^−1^). A small entropic gain was inferred from the ITC measurements on UBR4_4828–5130_, whereas an appreciable entropic penalty was inferred for UBR4_xtal_ (−*T*Δ*S* = −1.63 versus 5.85 kcal mol^−1^), suggestive of the N-terminal region losing conformational freedom upon UBE2A engagement (Fig. 7a,b). Although the structure and function of this region remain unknown, its ability to serve as an E2 affinity factor is suggestive of it having a role in E3 activity. This requires further investigation, but such a role would make the UBR4 module at least ∼400 residues, which is of unprecedented scale for a single-subunit allosteric E3.

## Discussion

Whilst UBR4 has been shown to destabilize N-degron substrates, the E3 module responsible has remained elusive. Herein we identify an allosteric E3 module within the giant N-degron E3 UBR4. While not discernible by primary sequence analysis, UBR4 contains an unusual zinc finger with partial structural similarity to the canonical cross-brace RING domain. However, only a single zinc ion is present, and substituting for the second zinc ion is a hydrogen bonding network, including a central water-mediated interaction. The fold is reminiscent of SP-RING domains found in SUMO E3s of the Siz/PIAS family but the absence of the zinc ion, distal to the E2-binding region, has not been observed before. The loop region that coordinates the first zinc ion and a proximal α-helix present in canonical RING domains mediate interactions with E2 conjugating enzymes. Our crystal structure of a complex with a cognate E2 (UBE2A) reveals that this property is shared with the hemiRING. However, we identify important interfacial UBE2A and hemiRING residues that provide insight into cognate E3 pairing with UBE2A/UBE2B. A quartet of arginine residues, characteristic of UBE2A/UBE2B, is probably central to its specific recognition by UBR4. As mutation of these and other interface residues are found in patients with XLID, our structure provides insight into disease etiology.

An unusual feature of the hemiRING is a pronounced loop. This loop, and perhaps the hemiRING itself, requires stabilization by an extreme C-terminal region composed of 11 α-helices, which we call the UZI domain. Using an allosteric mechanism, RING E3 prototypes robustly enhance E2∼Ub transfer activity by forming linchpin-mediated electrostatic interactions in combination with hydrophobic contacts. Since UBR4 lacks the linchpin residue, this results in attenuated enhancement of Ub transfer. This linchpin-independent mechanism involves allosteric contacts between UBR4 residues Gly4979 and Gly4980 within the UZI subdomain and Ub residue Ile36, as disruption of this interaction impairs autoubiquitination. We propose the intrinsically high state of UBE2A activity, presumably shared by UBE2B, explains why robust E3-mediated activation is unnecessary and cooperatively produces a level of UBR4 Ub transfer that ensures substrate specificity, and polyubiquitin chain processivity providing optimum degradation kinetics. The intrinsically high lysine reactivity of UBE2A/UBE2B may also explain their ability to induce efficient E3-independent targeted protein degradation. The basis for this reactivity is unclear but might be explained by the E3-independent stability of their closed conformations, or distinct features of their active sites^53^. As the UBR box is a known substrate binding domain that recognizes N-degrons, it probably positions recruited substrates near the UBE2A-bound active site (Fig. 7c and Supplementary Fig. 1). Cryo-electron microscopy analysis of the full-length protein should reveal how this is achieved and highlight other potential substrate-binding domains and regulatory elements. Interestingly, two of the six UBR4 isoforms lack the hemiRING–UZI module, suggestive of UBR4 having E3-independent functions. Furthermore, an isoform exists that contains a 21-residue insertion in the α7–α8 loop region of the UZI subdomain, but the functional relevance of this is unclear.

We established that the minimal construct that could undergo autoubiquitination consisted of residues 4,730–5,183, but in our crystal structure only residues 4,833–5,183 were resolved. Interestingly, the N-terminal region appreciably contributes to the free energy of binding to UBE2A as its deletion results in a 75-fold decrease in affinity. Its role in the optimal recruitment of E2 suggests the UBR4 E3 module is unusually large for a single-subunit allosteric E3. Interestingly, helical elements from other E3s have been shown to engage the backside of UBE2A and potentiate or attenuate substrate ubiquitination. Although such activity was not observed when using autoubiquitination as readout, in the context of physiological substrates the N-terminal region might similarly regulate E3 activity. This, and if the N-terminal helical region further contributes to E3 selectivity for UBE2A/UBE2B, remains to be tested.

The role of UBR4 E3 activity in promoting various diseases such as cancer and muscular atrophy would imply that modulation of UBR4 hemiRING E3 activity might have therapeutic value (for example, by disruption of the UBE2A–hemiRING interface). As such, the mechanistic and structural insights obtained herein could be leveraged to develop therapeutic modulators of UBR4-mediated substrate ubiquitination. Furthermore, although UBE2A/UBE2B have been shown to mediate efficient targeted protein degradation, they consist of a single Ubc domain, which has proven challenging to develop high-affinity and selective ligands^65,66^. The distinct structural features of the hemiRING–UZI module might provide a more tractable route to developing ligands that indirectly recruit UBE2A allowing its high Ub transfer activity to be exploited for therapeutic degrader applications.

Our findings uncover the high-resolution structure of a novel class of Ub E3 module and reveal the molecular insights into the selective recognition of UBE2A—an E2 that has not been structurally characterized in a complex with a cognate E3. Our work demonstrates that allosteric E3 modules remain to be identified that cannot be perceived by primary sequence analysis. Considering the discovery of unanticipated cysteine-dependent E3s^13,14^, which only represent a small subset of the E3 superfamily (<10%), it would seem probable that the scale and structural diversity of allosteric E3s is also underappreciated.

## Methods

### Expression and purification of UBR4 constructs

For cloning strategy and primers, refer to Supplementary Information. Glutathione *S*-transferase (GST)-tagged UBR4 (wild type (WT) and mutants) harboring a PreScission cleavage site were transformed into *E. coli* BL21(DE3) cells and grown overnight in a Luria-Bertani (LB) media starter culture supplemented with 200 µM zinc chloride and 100 μg ml^−1^ ampicillin at 37 °C with shaking. The starter culture was diluted 1:1,000 into fresh LB supplemented with 200 µM zinc chloride and 100 μg ml^−1^ ampicillin and incubated at 37 °C until an OD_600_ of 0.8 was reached. Protein expression was induced with 0.3 mM isopropyl β-d-1-thiogalactopyranoside, and cultures were incubated at 16 °C overnight.

Pellets were resuspended with buffer containing 20 mM HEPES–NaOH pH 7.4, 150 mM NaCl, 0.7 mM tris(2-carboxyethyl)phosphine (TCEP) with 0.5 mg ml^−1^ lysozyme, 50 µg ml^−1^ DNase. Samples were sonicated on ice and clarified via centrifugation at 30,000*g* for 45 min. Clarified lysates were incubated with glutathione sepharose 4B resin and washed via centrifugation with buffer containing 20 mM HEPES–NaOH pH 7.4, 150 mM NaCl and 0.7 mM TCEP. For elution, samples were incubated with 10 mM reduced glutathione for 10 min. For on resin tag cleavage, samples were incubated with C3 PreScission protease overnight at 4 °C. For crystallization, the eluted proteins were further purified by size-exclusion chromatography (HiLoad 16/600 Superdex 75 pg column or HiLoad 16/600 Superdex 200 pg column) using an ÄKTA Purifier FPLC system (20 mM HEPES–NaOH pH 7.4, 150 mM NaCl and 0.7 mM TCEP) at 1 ml min^−1^ and collected in 1-ml fractions. For biochemical and biophysical assays, size-exclusion chromatography was carried out with a phosphate buffer (50 mM Na_2_HPO_4_–HCl pH 7.4, 150 mM NaCl and 1 mM TCEP). Fractions of interest were then visualized via SDS–PAGE gel to assess purity and desired fractions were pooled and concentrated via spin concentrator, aliquoted and snap-frozen before storage at −80 °C.

### Expression and purification of E2 conjugating enzymes

With the exception of UBE2O, E2s were expressed in *E. coli* BL21(DE3) purified using glutathione Sepharose 4B or Ni-NTA resin, followed by size-exclusion chromatography. N-terminal tags were cleaved with PreScission protease for E2s expressed from pGEX, pET156P and pET15b vectors whereas thrombin was used for E2s expressed from the pET28a vector (pET156P His–UBE2B, pET156P His–UBE2C, pET28a His–UBE2D1, pET28a His–UBE2D4, pET156P His–UBE2L3, pET28 His–UBE2S, pGEX6P-3 UBE2A and pGEX6P-1 UBE2R2). The N-terminal His tag on the remaining E2s was left in place and expressed from the following plasmids: pET28 His–UBE2D2, pET156P His–UBE2D3, pET156P His–UBE2E1, pET28a His–UBE2E2, pET28a His–UBE2E3, pET28a His–UBE2G1, pET28a His–UBE2G2, pET156P His–UBE2H, pET28a His–UBE2J1, pET28a His–UBE2J2, pET156P His–UBE2K, pET15b His–UBE2N, pET28a His–UBE2Q1, pET15b His–UBE2Q2, pET28a His–UBE2R1, pET15b6P His–UBE2T, pET28a His–UBE2V1, pET15b His–UBE2V2, pET28a His–UBE2W and pET15b His–UBE2Z. UBE2O was expressed in Sf9 insect cells (ThermoFisher). Protein was purified using Ni-NTA affinity followed by size-exclusion chromatography, and the His tag was left in place.

### Autoubiquitination assays

Assays were made up from a 10× buffer (400 mM Na_2_HPO_4_, 1.5 M NaCl and 10 mM TCEP) containing final concentrations of 40 mM Na_2_H_2_PO_4_ pH 8, 150 mM NaCl, 5 mM MgCl_2_, 1 mM TCEP, ATP (5 mM), cleaved UBR4 construct (3 µM), E2 (5 µM), E1 (0.5 µM) and Ub (50 µM). Reactions were incubated at 37 °C for 30 min and quenched by dilution with 4× lithium dodecyl sulfate (LDS) buffer containing 680 mM 2-mercaptoethanol, resolved via SDS–PAGE gel and then visualized by Coomassie staining or western blotting.

For full-length UBR4 autoubiquitination assays, HEK293 cells stably overexpressing HA–UBR4 were lysed in buffer (50 mM Tris–HCl pH 7.5, 1 mM egtazic acid, 1 mM ethylenediaminetetraacetic acid, 10 mM glycerophosphate, 50 mM sodium fluoride, 5 mM sodium pyrophosphate, 1 mM sodium vanadate, 0.27 M sucrose, 1% NP-40, 0.2 mM phenylmethylsulfonyl fluoride, 1 mM benzamidine and 1 mM TCEP) supplemented with complete ethylenediaminetetraacetic acid-free protease inhibitor cocktail (Roche 11873580001). The lysate was then centrifuged for 10 min at 16,200*g*, and the supernatant was collected. Full-length HA–UBR4 was immunoprecipitated using anti-HA sepharose resin for 1 h at 23 °C, followed by a wash in phosphate buffer (50 mM sodium phosphate pH 7.5, 150 mM NaCl and 1 mM TCEP). For the E2 panel, the resin was combined with a reaction mix containing UBE1 (500 nM), Ub (5 µM), ATP (10 mM) and the specified E2 (10 µM for UBE2A, UBE2B, UBE2C, UBE2D1, UBE2D2, UBE2D3, UBE2D4, UBE2E1, UBE2E2, UBE2G2, UBE2H, UBE2K, UBE2L3, UBE2N, UBE2Q1, UBE2Q2, UBE2R1, UBE2T, UBE2V1, UBE2V2, UBE2W and UBE2Z; 5 µM for UBE2E3, UBE2G1, UBE2J1, UBE2J2, UBE2R2 and UBE2S; 1.5 µM for UBE2O) in 50 mM sodium phosphate pH 7.5, 150 mM NaCl, 5 mM MgCl_2_ and 0.75 mM TCEP. Concentrations of 12.5 µM Ub and 1 mM TCEP were used for experiments with full-length UBR4 mutants. Reactions were incubated at 37 °C for 1 h and quenched by the addition of LDS loading buffer containing β-mercaptoethanol (Invitrogen NP0007). Samples were heated for 10 min at 70 °C and loaded on 4–12% Bis-Tris (Invitrogen NP0323) or 3–8% Tris-acetate (Invitrogen EA03785) polyacrylamide gels for Coomassie stain (Instant Blue Abcam AB119211) or western blotting, respectively.

### Crystallization of UBR4_xtal_ and UBR4_xtal_–UBE2A complex

Initially, commercially available crystallization conditions were screened in 96-well format. Proteins for crystal screening were expressed and purified as above for cleaved protein. Plates were set up with Mosquito Crystal and Dragonfly liquid handling robots (SPT Labtech). Plates were then sealed and incubated at either 4 °C or room temperature and monitored. Conditions yielding crystals (10 mM Na_2_HPO_4_, pH 6.5, 13% PEG20,000, 22 °C and 4 °C) were replicated on a larger scale in 24-well plates, which were incubated at room temperature. One milliliter total volume of each condition was placed in each well and covered with a glass cover slip carrying a 2 µl hanging drop (1 µl protein, 1 µl buffer condition) with protein concentration (5 mg ml^−1^ final per drop). For the UBR4_xtal_–UBE2A complex, the 6xHis tag was cleaved from a UBE2A Cys88Lys mutant (DU 65350) with tobacco etch virus (TEV) protease and was mixed with an equimolar amount of cleaved GST PreScission UBR4_xtal_ (DU 65064), and crystals were obtained in hanging drops 0.1 M Bis-Tris, pH 6.4, 15 % PEG10,000 and 0.2 M ammonium acetate.

Crystals were collected and cryo-protected with the well condition supplemented with 25% ethylene glycol followed by plunge vitrification in liquid nitrogen. Crystals were screened via remote collection at Diamond Light Source beam lines I24 (UBR4xtal) or I04-1 (UBE2A-UBR4xtal). For UBR4_xtal_ crystals, an X-ray fluorescence scan at the zinc K-absorption edge was performed. Based on the scan the peak wavelength was chosen as *λ* = 9,671.0 eV (1.2820 Å) at 100 K and the inflection point wavelength as *λ* = 9,663.0 eV (1.2831 Å). Data were collected at the zinc edge to allow measurement of the anomalous signal for phasing. The data were indexed, integrated and scaled using DIALS (ver. 2.0.2)^67^ and phased using CRANK2 (ver. 2.0.1). The high-resolution cut-off was selected on the basis of the CC_1/2_ > 0.5, where CC_1/2_ is the Pearson correlation coefficient between reflection intensities from randomly selected halves of the datasets. Refinement was carried out with Phenix (ver. 1.17.1)^68^, between rounds of refinement models were manually improved using Coot^69^ and Final Ramachandran statistics were: favored 98.54%, allowed 1.46% and outliers 0.00%. Clash score was 3.54.

The UBE2A–UBR4xtal complex data were collected at 0.9118 Å at 100 K and indexed and integrated using DIALS. Data were scaled and merged using Aimless (0.7.4). Phasing was achieved by molecular replacement with Phaser (ver. 2.8.3) using the UBR4_xtal_ structure and UBE2A (Protein Data Bank (PDB) 6CYO)^53^ as search models. Refinement was carried out with Phenix (ver. 1.17.1)^68^, and between rounds of refinement models were manually improved using Coot (ver. 0.9.5)^69^. Final Ramachandran statistics were: favored 95.70%, allowed 4.30% and outliers 0.00%. Clash score was 4.42.

### Preparation of Cy3b-labeled Ub

Ub with an N-terminal His tag followed by a cysteine residue for Cy3b conjugation and a TEV cleavage site (DU 29939) was expressed in BL21 cells as described above and purified using Ni-affinity chromatography. The His tag was cleaved with TEV protease as described above, and the protein was buffer exchanged into degassed buffer containing 50 mM HEPES–NaOH, pH 7.5, 0.5 mM TCEP before dye conjugation. Protein was concentrated to 2 mg ml^−1^, and 200 µl was mixed with Cy3b–maleimide for a final concentration of 150 nM in 300 µl. Protein was then incubated at 25 °C for 2 h with agitation. The reaction was monitored by liquid chromatography–mass spectrometry (Agilent 1200 HPLC, 6130 Single Quad) and then purified using a P2 Centri-Pure desalting column using the same degassed buffer. Concentration was then determined by spectrophotometry, and the protein was aliquoted, snap-frozen and stored at −80 °C.

### UBR4 autoubiquitination under single turnover E2∼Ub discharge conditions

6xHis cleaved WT or mutant E2s (10 µM) were charged with Ub labeled with Cy3b at a concentration of 12.5 µM, in a buffer (20 mM Na_2_H_2_PO_4_ pH 8, 150 mM NaCl, 5 mM MgCl_2_ and 1 mM TCEP), along with 0.5 µM E1 and 5 mM ATP for 20 min at 37 °C. Samples were cooled on ice for 2 min followed by the addition of pan E1 inhibitor Compound 1 (25 µM) as previously described^14^, and incubated for 10 min at room temperature. This was then mixed with an equal volume of either UBR4 (WT or mutant) 5 µM or buffer. Samples were taken at the indicated time points and quenched with 4× LDS loading dye (ThermoFisher). Gels were imaged via ChemiDoc, and data were analyzed via ImageJ and Prism.

### Lysine discharge assay

WT or mutant E2s (10 μM) were charged with labeled Ub (Cy3/Cy3b/Cy5) (12.5 μM) as above. An equal volume of a sample containing 20 mM Na_2_PO_4_pH ∼7.5, 20 mM l-lysine, or buffer alone for control reactions, was added and a time point was taken at *t* = 0. Further samples were taken at the indicated time points and quenched with 4× LDS loading buffer (ThermoFisher).

### Transient transfection of WT and mutant full-length UBR4

HEK293 cells (ATCC) were grown in 100-mm dishes, and transfected with WT, Cys4890Ala or His4887Ala N-terminal HA-tagged UBR4 coding plasmids (DU 71005 and 71006), or the empty vector, using Lipofectamine 2000 (Invitrogen 11668-019). Briefly, plasmid DNA (10 µg) and Lipofectamine 2000 (25 µg) were diluted in Opti-MEM (Gibco 31985-062), combined, and incubated at room temperature for 20 min before adding onto cells. Cells were maintained in Dulbecco’s modified Eagle medium (Gibco 11960-085) 10% fetal bovine serum (Sigma F7524), 100 U ml^−1^ penicillin–streptomycin (Gibco 15140122), 2 mM l-glutamine (Gibco 25030024) at 37 °C in 5% CO_2_, and a period of 24 h post-transfection was observed before collection to allow protein expression.

### Stable UBR4-expressing cell line

Full-length UBR4 stable cell lines were created by co-transfecting untagged (DU65532) or N-terminal HA-tagged UBR4 (DU65964) coding plasmids (7.5 µg) and pOG44 (2.5 µg) into HEK293 Flp-In T-REx cells (Thermo Fisher Scientific). Selection and maintenance of cells that underwent recombination started 24 h later by including hygromycin (50 µg ml^−1^, Invivogen ant-hg-5) in the culture media. Induction of UBR4 expression was achieved by supplementing media with tetracycline (1 µg ml^−1^, Sigma T7660).

### Western blotting

Electrophoresis was performed at 200 V and transferred on polyvinylidene fluoride membrane using a Tris-glycine buffer (48 mM Tris, 39 mM glycine and 20% methanol) at 95 V for 3 h. Membranes were incubated 1 h in 5% milk before adding the primary antibody (anti-HA 3F10, Roche 27573500, 1:2,500, anti-Ub P4D1, BioLegend, 1:10,000, anti-UBR4/p600 ab86738, Abcam, 1:5,000, anti-FLAG M2, Sigma F1804, 1:5,000, anti-Vinculin ab129002, Abcam, 1:10,000). After rinsing, secondary antibody was added (anti-rat Cell Signaling 7077S, 1:5,000, anti-mouse Cell Signaling, 7076S, 1:5,000, anti-rabbit IRDye 680RD, LI-COR 926-68071, 1:20,000, anti-mouse IRDye 800CW, LI-COR 926-32210, 1:20,000). Following further washing, some membranes were incubated in chemiluminescent substrate (ECL Pierce 32106). Chemiluminescence was captured by radiographic films or an electronic imaging system (ChemiDoc MP Bio-Rad) running Imagelab Touch Software (ver. 2.3.0.07). The near-infrared signal was assessed using an LI-COR Odyssey CLx instrument running Image Studio (ver. 5.2).

### ITC

ITC experiments were carried out using a MicroCal PEAQ-ITC instrument operated with PEAQ-ITC Software (ver. 1.40) (Malvern Panalytical). UBR4 constructs were placed in the cell (74 μM), and UBE2A was placed in the syringe at a tenfold higher concentration. Experiments utilized an initial injection (0.4 µl) followed by 13 injections (3 µl). A reference response of UBE2A titrated into buffer was subtracted using the manufacturer software routine to account for the dilution enthalpy of the titrant. All experiments were carried out at 25 °C, and the data generated were analyzed using manufacturer software and PRISM (GraphPad) for figure generation.

### Ubiquitination site mapping by liquid chromatography–tandem mass spectrometry analysis

Peptides generated by trypsin treatment of the excised gel slice were resuspended in 5% formic acid in water and injected on an UltiMate 3000 RSLCnano System coupled to an Orbitrap Fusion Lumos Tribrid Mass Spectrometer (Thermo Fisher Scientific). Peptides were loaded on an Acclaim Pepmap trap column (Thermo Fisher Scientific #164750) with prior analysis on a PepMap RSLC C18 analytical column (Thermo Fisher Scientific #ES903) and eluted on a 120-min linear gradient from 3% to 35% Buffer B (Buffer A: 0.1% formic acid in water, Buffer B: 0.08% formic acid in 80:20 acetonitrile:water (v:v)). Eluted peptides were then analyzed by the mass spectrometer operating in data-dependent acquisition mode. Peptides were searched against a reduced database containing only the four proteins used in this assay (Ub, His–UBA1, UBE2A and UBR4) using MaxQuant (v2.1.3.1)^70^. All parameters were left as default except for the addition of Deamidation (N, Q) and GlyGly (Protein N-term, K, C, S, T, Y) as variable modifications and with the PSM, Protein and Site FDR increase to 1.00. Tandem mass spectrometry spectra of interesting GlyGly peptides were manually inspected.

### Reporting summary

Further information on research design is available in the Nature Portfolio Reporting Summary linked to this article.

## Online content

Any methods, additional references, Nature Portfolio reporting summaries, source data, extended data, supplementary information, acknowledgements, peer review information; details of author contributions and competing interests; and statements of data and code availability are available at 10.1038/s41594-023-01192-4.

### Supplementary information

Supplementary Information Supplementary Notes 1 and 2, Tables 1–11, Figs. 1–21 and data.

Reporting Summary

Supplementary Data 1 Cloning strategy and primers used.

### Source data

Source Data Fig. 1 Full blot for Fig. 1b.

Source Data Fig. 2 Full gels and blot for Fig. 2b,c,g.

Source Data Fig. 6 Full gels for Fig. 6b,e–g.

Source Data Extended Data Fig. 2 Full gels for Extended Data Fig. 2b,c.

Source Data Extended Data Fig. 3 Full blot for Extended Data Fig. 3b.

Source Data Extended Data Fig. 5 Source data for Extended Data Fig. 5b,d,f.

Source Data Extended Data Fig. 7 Source data for Extended Data Fig. 7d.

Source Data Extended Data Fig. 8 Source data for Extended Data Fig. 8b–d.

Source Data Extended Data Fig. 9 Source data for Extended Data Fig. 9a–c.

## Data Availability

Protein structure coordinates have been deposited with the Protein Data Bank (https://www.rcsb.org). PDB codes for UBR4_xtal_ and the UBR4xtal–UBE2A complex are 8B5W and 8BTL, respectively. Coordinates for the previously reported RNF4 and RNF4:E2∼Ub structures have been deposited with ID 4AP4 and 4PPE, respectively. Raw mass spectrometry data have been deposited with Pride (https://www.ebi.ac.uk/pride/) with accession number PXD046899. Full gels and all replicate data are available in Supplementary Information. Source data are provided with this paper.
