# Supplementary material for: UBE2A and UBE2B are recruited by an atypical E3 ligase module in UBR4
Source: Nat Struct Mol Biol. 2024 Jan 5;31(2):351–63. doi: 10.1038/s41594-023-01192-4 (PMC10873205; doi:10.1038/s41594-023-01192-4)

nature structural & molecular biology

Article

https://doi.org/10.1038/s41594-023-01192-4

UBE2A and UBE2B are recruited by an atypical E3 ligase module in UBR4

In the format provided by the authors and unedited

Supplementary Figure 1

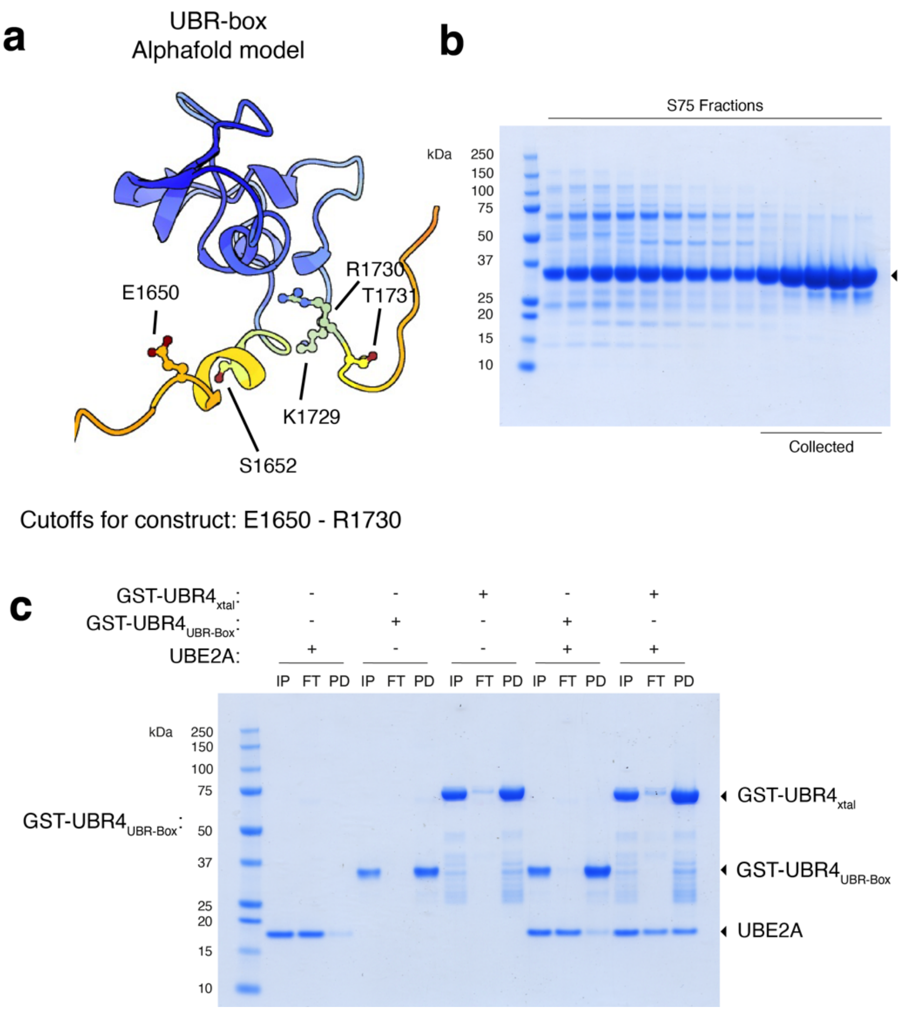

Supplementary Figure 1. The UBR4 U-box domain does not bind UBE2A. a) Alphafold model of the UBR4 UBR-Box domain. A GST-tagged construct consisting of residues 1650-1730 was used ($\text{UBR4}_{\text{UBR-Box}}$). **b)** Purification of GST-$\text{UBR4}_{\text{UBR-Box}}$ by size exclusion chromatography. **c)** GST-$\text{UBR4}_{\text{xtal}}$ and GST-$\text{UBR4}_{\text{UBR-Box}}$ were immobilized on glutathione Sepharose 4B resin and incubated with UBE2A. The resin was then washed, and to test for an interaction with UBE2A, the resin was stripped with LDS sample buffer and analyzed by SDS-PAGE (IP; pre-GST resin), flow through (FT; post bead incubation) and pulldown (PD; boiled resin).

Figure 7a ITC repeat

UBR4$_{xtal}$

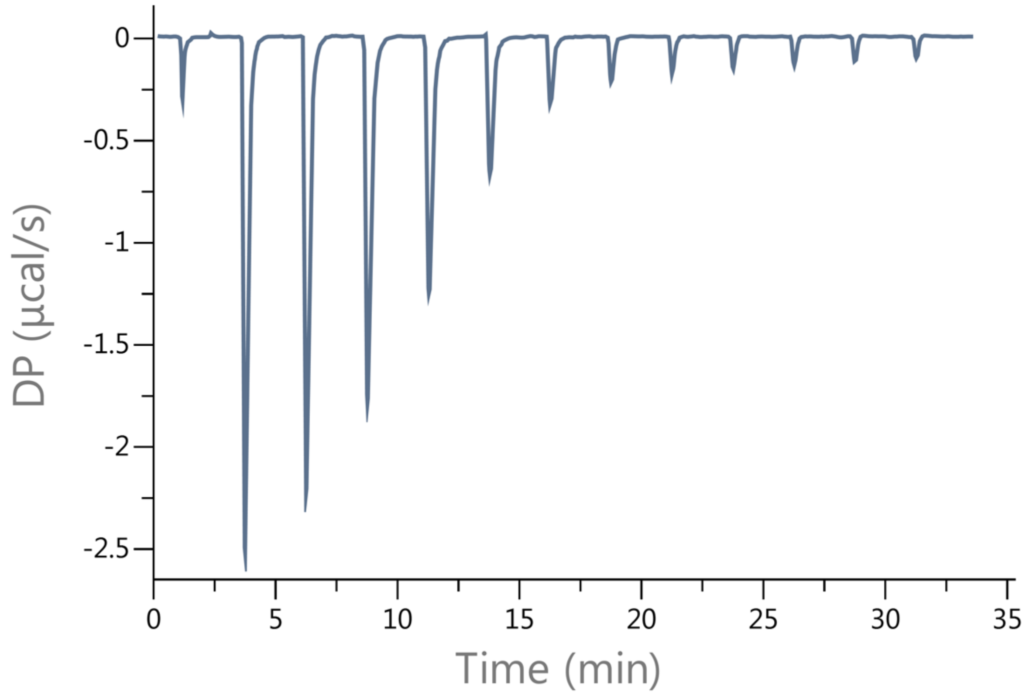

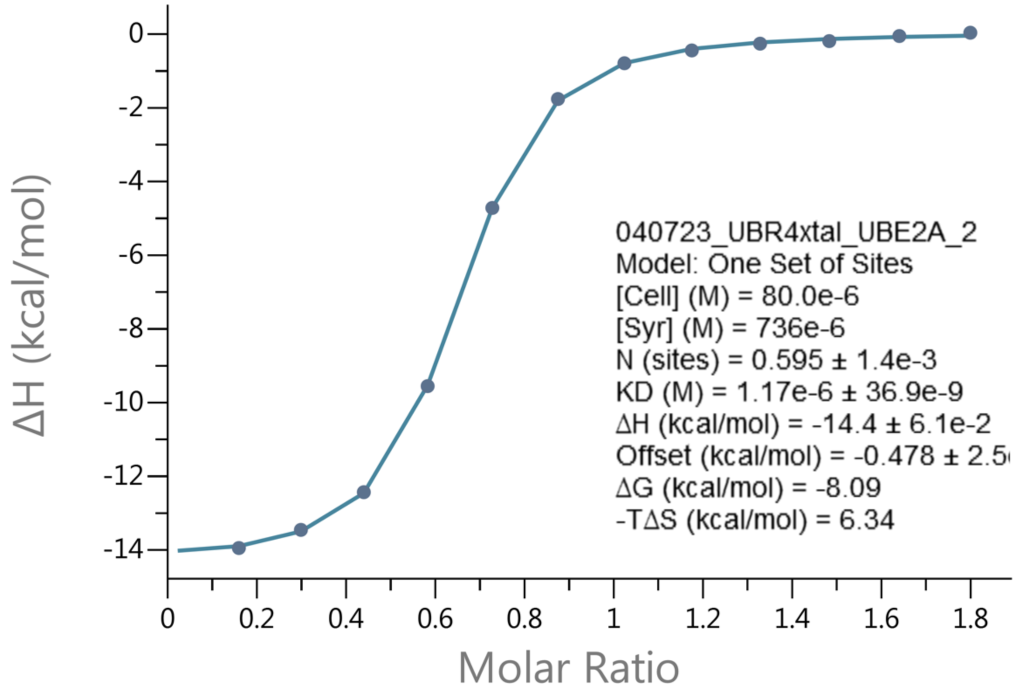

Figure 7b ITC repeat

UBR4$_{4928\text{-}5183}$

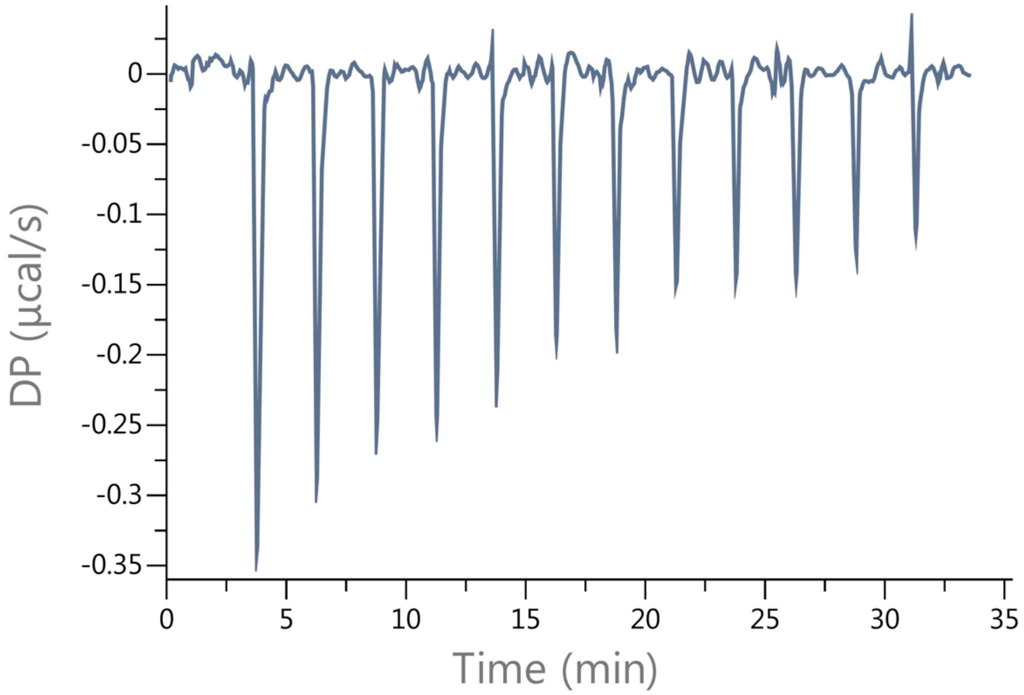

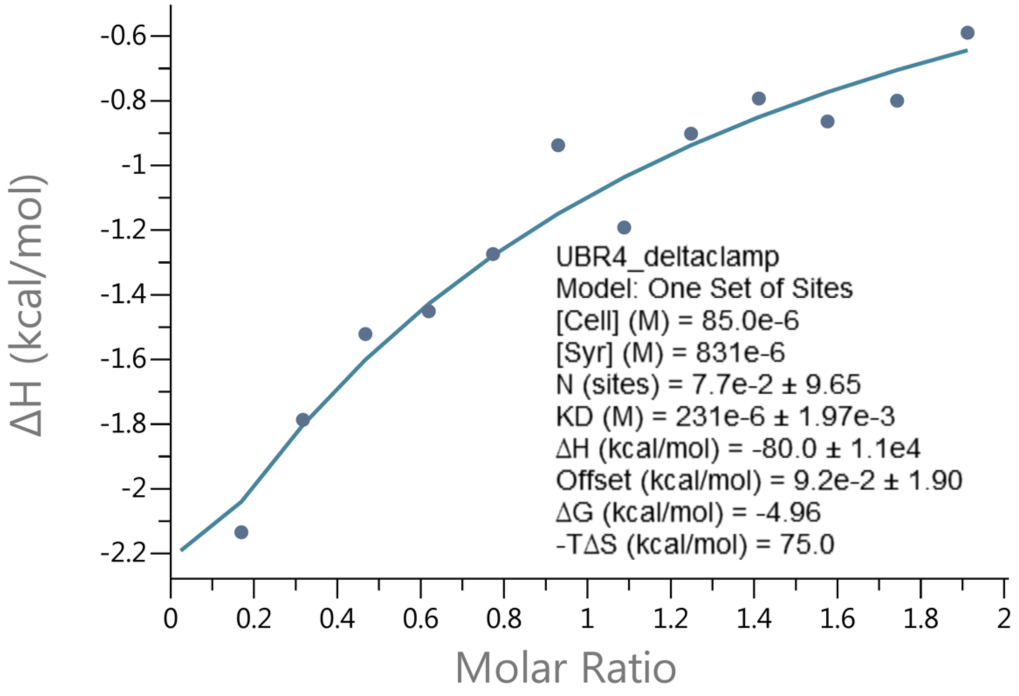

Supplement: Supplementary file 1 — Supplementary Notes 1 and 2, Tables 1–11, Figs. 1–21 and data. [file 41594_2023_1192_MOESM1_ESM.pdf]
