# Supplementary material for: UBE2A and UBE2B are recruited by an atypical E3 ligase module in UBR4
Source: Nat Struct Mol Biol. 2024 Jan 5;31(2):351–63. doi: 10.1038/s41594-023-01192-4 (PMC10873205; doi:10.1038/s41594-023-01192-4)
Supplement: Supplementary file 2 — Reporting Summary [file 41594_2023_1192_MOESM2_ESM.pdf]

nature portfolio

Corresponding author(s): Satpal Virdee

Last updated by author(s): Nov 13, 2023

# Reporting Summary

Nature Portfolio wishes to improve the reproducibility of the work that we publish. This form provides structure for consistency and transparency in reporting. For further information on Nature Portfolio policies, see our Editorial Policies and the Editorial Policy Checklist.

## Statistics

For all statistical analyses, confirm that the following items are present in the figure legend, table legend, main text, or Methods section.

| n/a | Confirmed | |
|---|---|---|
| ☐ | ☒ | The exact sample size (*n*) for each experimental group/condition, given as a discrete number and unit of measurement |
| ☐ | ☒ | A statement on whether measurements were taken from distinct samples or whether the same sample was measured repeatedly |
| ☒ | ☐ | The statistical test(s) used AND whether they are one- or two-sided<br>*Only common tests should be described solely by name; describe more complex techniques in the Methods section.* |
| ☒ | ☐ | A description of all covariates tested |
| ☐ | ☒ | A description of any assumptions or corrections, such as tests of normality and adjustment for multiple comparisons |
| ☐ | ☒ | A full description of the statistical parameters including central tendency (e.g. means) or other basic estimates (e.g. regression coefficient) AND variation (e.g. standard deviation) or associated estimates of uncertainty (e.g. confidence intervals) |
| ☒ | ☐ | For null hypothesis testing, the test statistic (e.g. *F*, *t*, *r*) with confidence intervals, effect sizes, degrees of freedom and *P* value noted<br>*Give P values as exact values whenever suitable.* |
| ☒ | ☐ | For Bayesian analysis, information on the choice of priors and Markov chain Monte Carlo settings |
| ☒ | ☐ | For hierarchical and complex designs, identification of the appropriate level for tests and full reporting of outcomes |
| ☒ | ☐ | Estimates of effect sizes (e.g. Cohen's *d*, Pearson's *r*), indicating how they were calculated |

*Our web collection on statistics for biologists contains articles on many of the points above.*

## Software and code

Policy information about availability of computer code

| | |
|---|---|
| Data collection | Imagelab Touch Software (Biorad) (ver. 2.3.0.07)<br>Image Studio (ver. 5.2) (Li-Cor Biosciences)<br>Oxford UK beam lines I24 (UBR4xtal) or I04-1813 (UBE2A-UBR4xtal)<br>PEAQ-ITC Software (Malvern Panalytical) (ver. 1.40) |
| Data analysis | Graphpad Prism (ver. 9.3.1)<br>ImageJ (ver. 2.3.0/1.53q)<br>DIALS (ver. 2.0.2) - integration and scaling<br>CRANK2 (ver. 2.0.1) - phasing from anomalous signal<br>Phenix (ver. 1.17.1) - refinement<br>Coot (ver. 0.9.5) - manual model building<br>MaxQuant (v2.1.3.1)<br>PEAQ-ITC Software (Malvern Panalytical) (ver. 1.40)<br>Jalview (ver. 2.11.2.5) |

For manuscripts utilizing custom algorithms or software that are central to the research but not yet described in published literature, software must be made available to editors and reviewers. We strongly encourage code deposition in a community repository (e.g. GitHub). See the Nature Portfolio guidelines for submitting code & software for further information.

## Data

Policy information about availability of data

All manuscripts must include a data availability statement. This statement should provide the following information, where applicable:

- Accession codes, unique identifiers, or web links for publicly available datasets
- A description of any restrictions on data availability
- For clinical datasets or third party data, please ensure that the statement adheres to our policy

Structure coordinates for UBR4 and UBR4-UBE2A complex are deposited with the PDB Protein Data Bank with ID 8B5W and 8BTL. Coordinates for the previously reported RNF4 and RNF4:E2~Ub structures have been deposited with ID 4AP4 and 4PPE, respectively. Raw mass spectrometry data have been deposited with Pride https://www.ebi.ac.uk/pride/ with accession number PXD046899. Full gels and all replicate data are available in the supporting information.

## Research involving human participants, their data, or biological material

Policy information about studies with human participants or human data. See also policy information about sex, gender (identity/presentation), and sexual orientation and race, ethnicity and racism.

| | |
|---|---|
| Reporting on sex and gender | N/A |
| Reporting on race, ethnicity, or other socially relevant groupings | N/A |
| Population characteristics | N/A |
| Recruitment | N/A |
| Ethics oversight | N/A |

Note that full information on the approval of the study protocol must also be provided in the manuscript.

# Field-specific reporting

Please select the one below that is the best fit for your research. If you are not sure, read the appropriate sections before making your selection.

☒ Life sciences ☐ Behavioural & social sciences ☐ Ecological, evolutionary & environmental sciences

For a reference copy of the document with all sections, see nature.com/documents/nr-reporting-summary-flat.pdf

# Life sciences study design

All studies must disclose on these points even when the disclosure is negative.

| | |
|---|---|
| Sample size | Activity measurements were carried out at least twice but usually in triplicate. This is typical in the field for assays of this type. |
| Data exclusions | No data were excluded. |
| Replication | All experiments were carried out at least twice, with triplicate being typical. The precise number of repeats is stated in the figure legends. |
| Randomization | No animals were employed |
| Blinding | No animals were employed |

# Reporting for specific materials, systems and methods

We require information from authors about some types of materials, experimental systems and methods used in many studies. Here, indicate whether each material, system or method listed is relevant to your study. If you are not sure if a list item applies to your research, read the appropriate section before selecting a response.

## Materials & experimental systems

| n/a | Involved in the study | |
|---|---|---|
| ☐ | ☒ | Antibodies |
| ☐ | ☒ | Eukaryotic cell lines |
| ☒ | ☐ | Palaeontology and archaeology |
| ☒ | ☐ | Animals and other organisms |
| ☒ | ☐ | Clinical data |
| ☒ | ☐ | Dual use research of concern |
| ☒ | ☐ | Plants |

## Methods

| n/a | Involved in the study | |
|---|---|---|
| ☒ | ☐ | ChIP-seq |
| ☒ | ☐ | Flow cytometry |
| ☒ | ☐ | MRI-based neuroimaging |

# Antibodies

Antibodies used

anti-HA 3F10, Roche 27573500, 1:2500, Manufacturer validated for specificity and sensitivity by serial dilution of HA-tagged GST into eukaryotic cell line.
anti-Ubiquitin P4D1, Biolegend, 1:10,000. Manufacturer validated with recombiant protein and MG-132 treated eukaryotic cells
anti-UBR4/p600 ab86738, Abcam, 1:5000 Manufacturer validated by measuring the appearance of the anticipated molecular weight band.
anti-FLAG M2, Sigma F1804, 1:5000, Manufacturer validated for specificity and sensitivity by serial dilution of FLAG-tagged BAP into eukaryotic cell line.
anti-Vinculin ab129002, Abcam, 1:10,000, Manufacturer validated by measuring the appearance of the anticipated molecular weight band.
anti-rat Cell Signaling 7077S, 1:5000, Secondary Ab, species reactivity confirmed within.
anti-mouse Cell Signaling, 7076S, 1:5000, econdary Ab, species reactivity confirmed within.
anti-rabbit IRDye 680RD, LI-COR 926-68071, 1:20,000, Secondary Ab, species reactivity confirmed within.
anti-mouse IRDye 800CW, LI-COR 926-32210, 1:20,000, Secondary Ab, species reactivity confirmed within.

Validation

Manufacturer datasheets (and see above)

# Eukaryotic cell lines

Policy information about cell lines and Sex and Gender in Research

Cell line source(s)

HEK293 Flp-In T-REx cells (Thermofisher)
HEK293 (ATCC)
sf9 cells (Thermofisher)

Authentication

Cells were not reauthenticated after receipt from suppliers

Mycoplasma contamination

Routeine mycoplasma testing was carried out in accordance with departmental procedures and tests were negative.

Commonly misidentified lines
(See ICLAC register)

None
