# Supplementary figures and images for: UBE2A and UBE2B are recruited by an atypical E3 ligase module in UBR4
Source: Nat Struct Mol Biol. 2024 Jan 5;31(2):351–63. doi: 10.1038/s41594-023-01192-4 (PMC10873205; doi:10.1038/s41594-023-01192-4)

[illegible]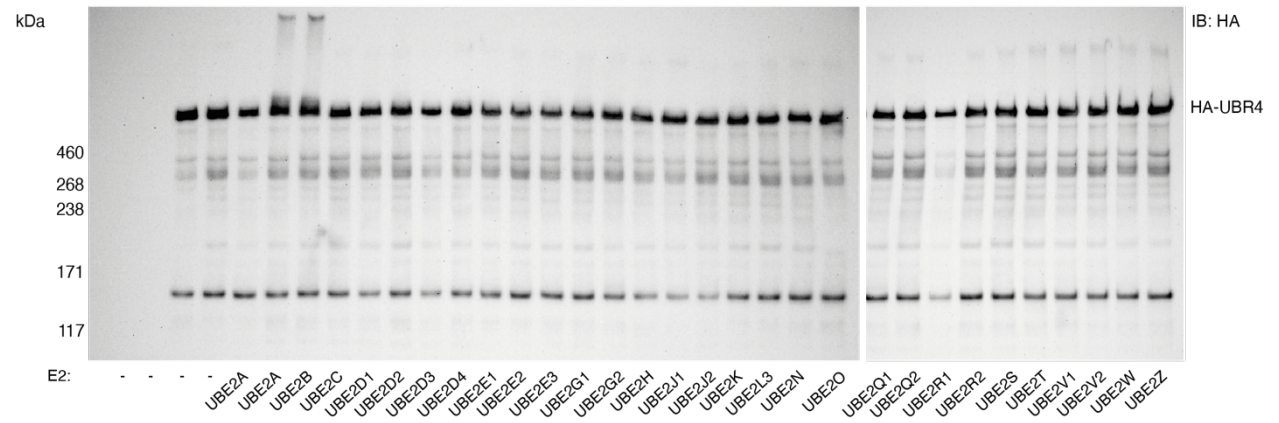

Supplement: Supplementary file 4 — Full blot for Fig. 1b. [file 41594_2023_1192_MOESM4_ESM.pdf]

Source data Figure 2b & c

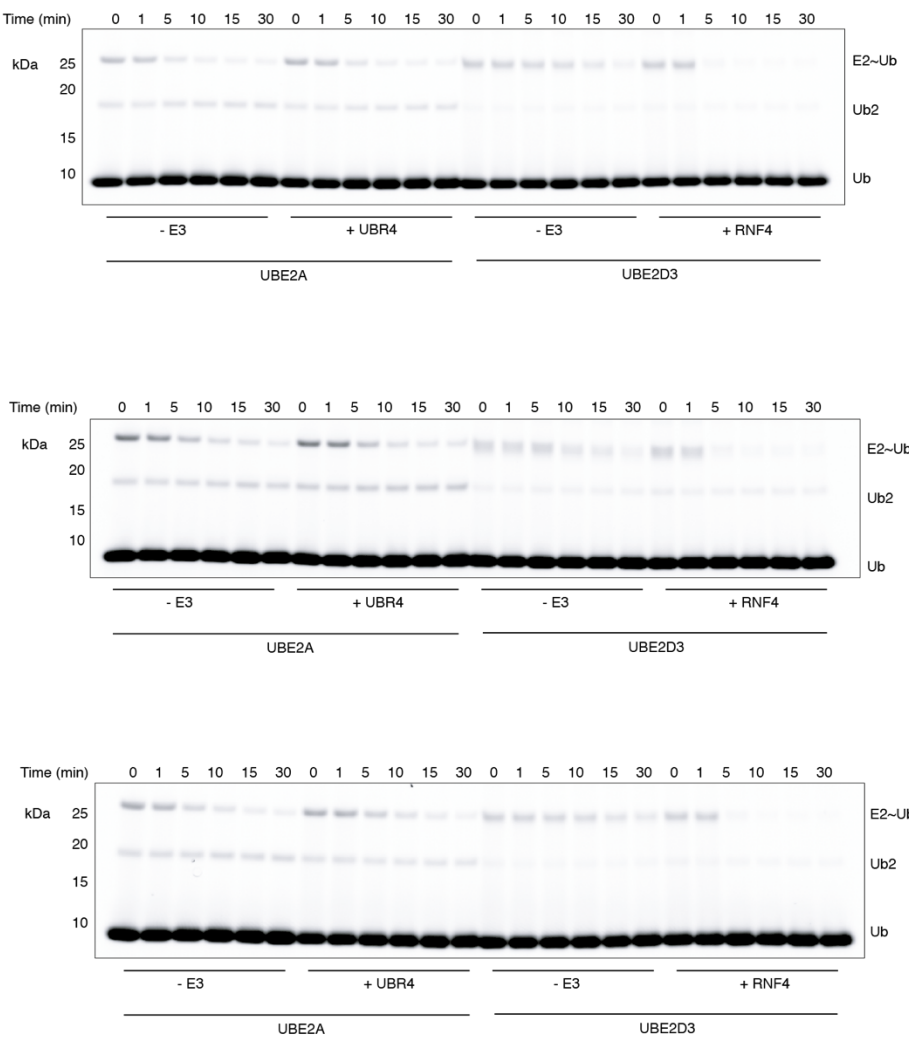

Source data Figure 2g

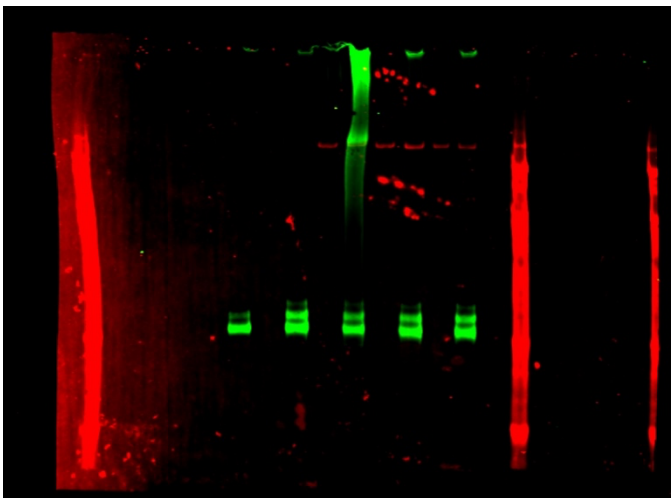

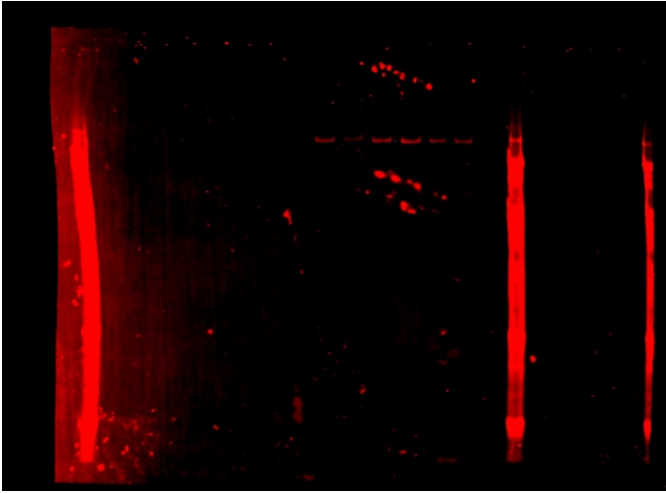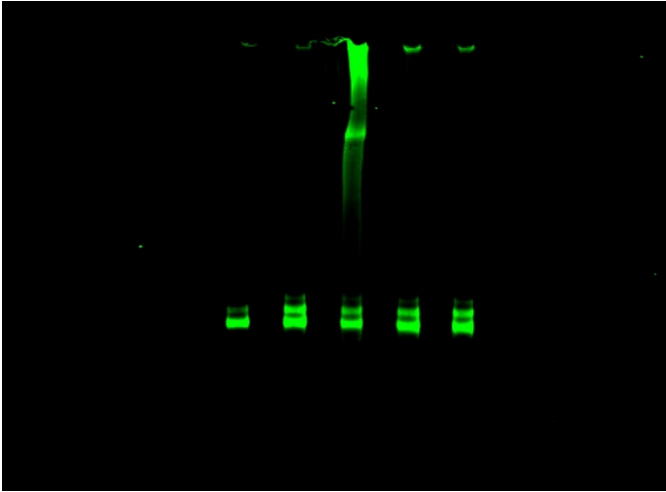

Supplement: Supplementary file 5 — Full gels and blot for Fig. 2b,c,g. [file 41594_2023_1192_MOESM5_ESM.pdf]

Source data Figure 6b

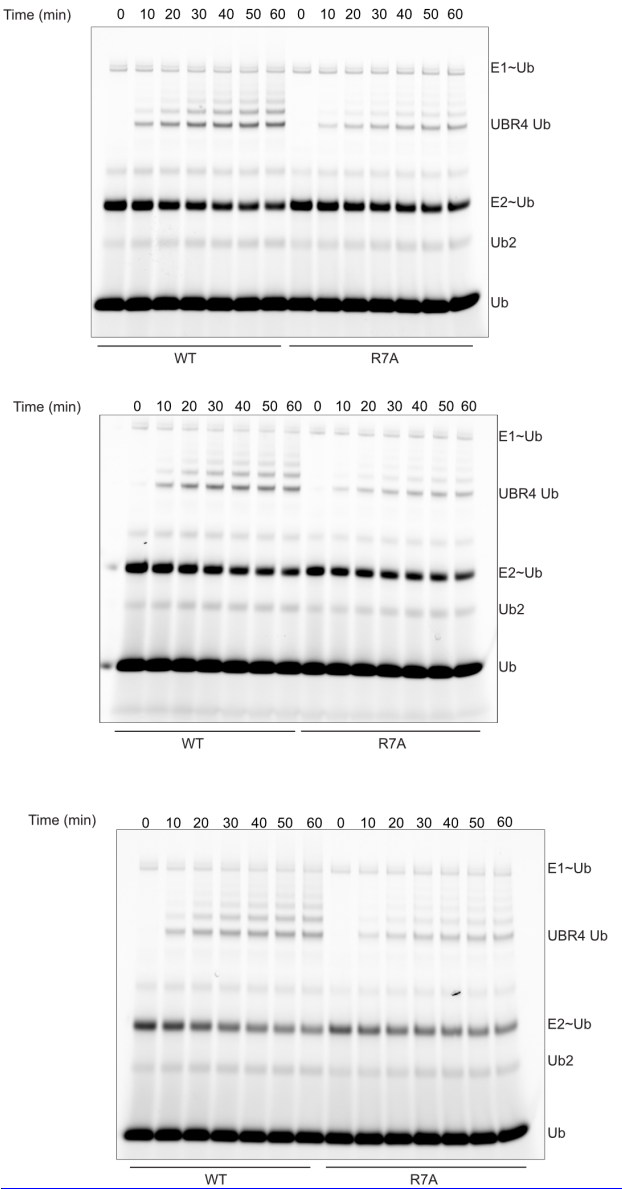

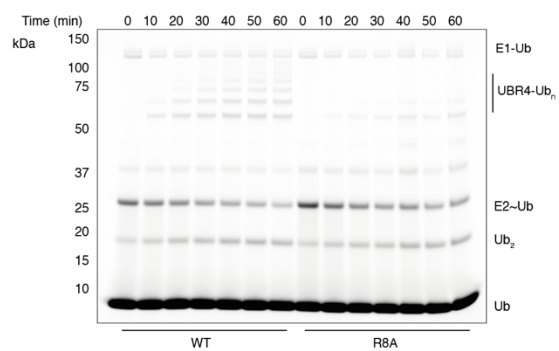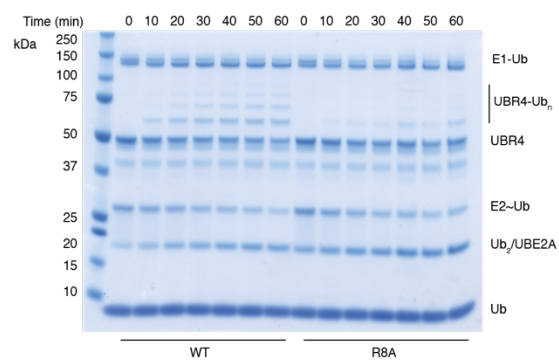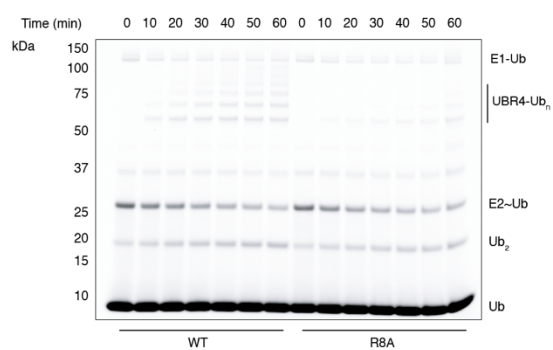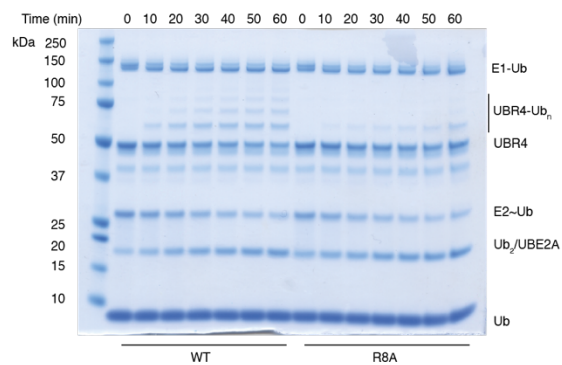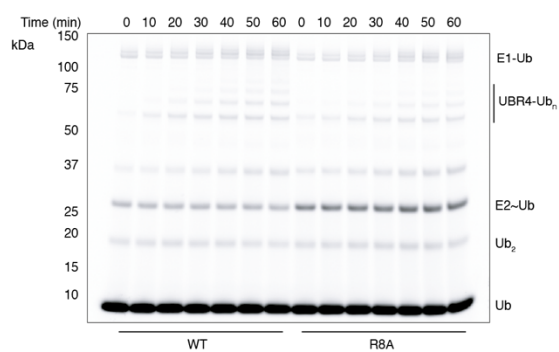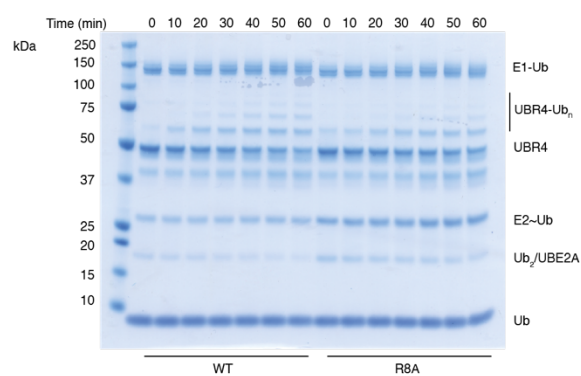

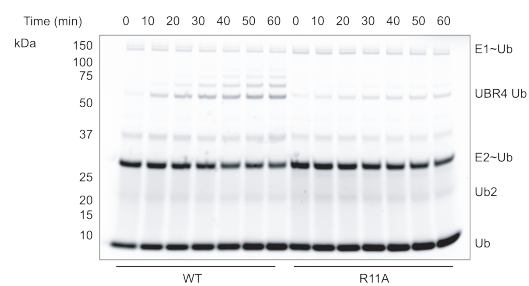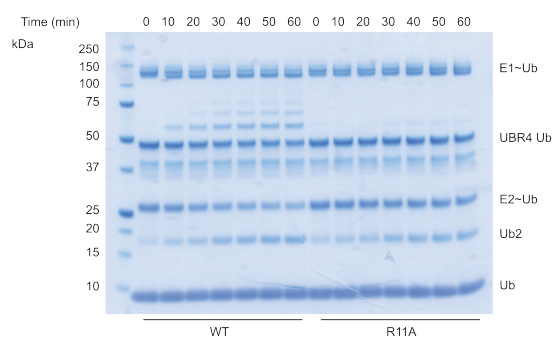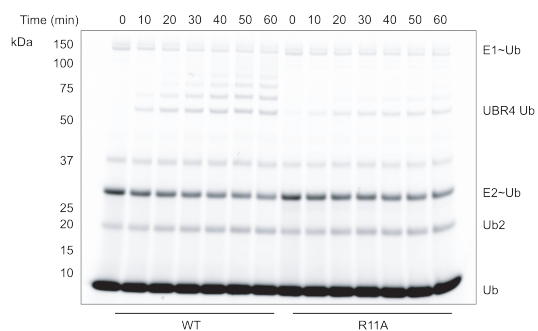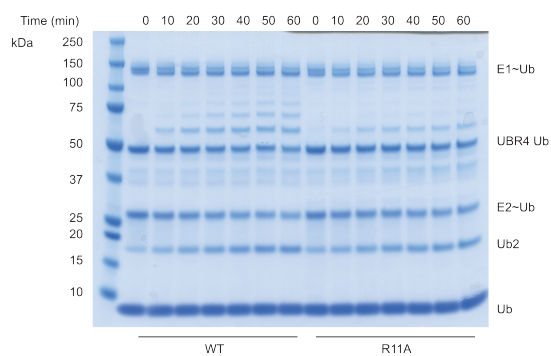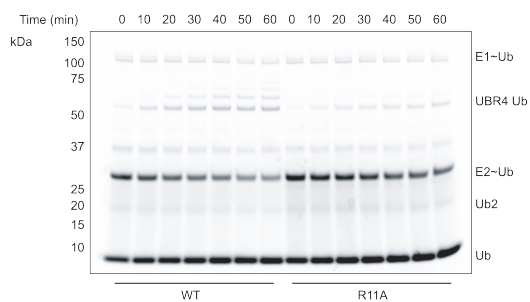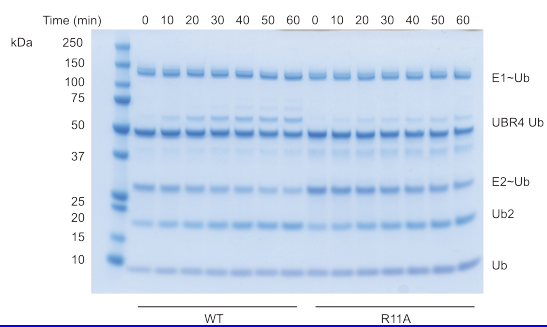

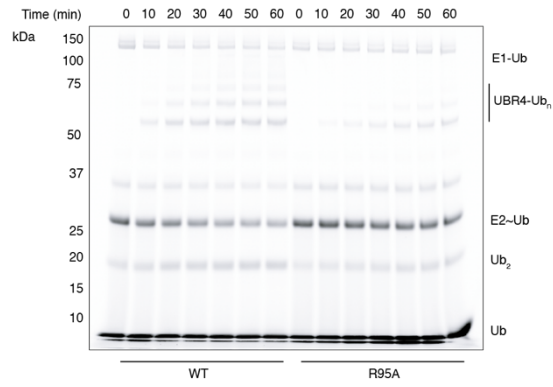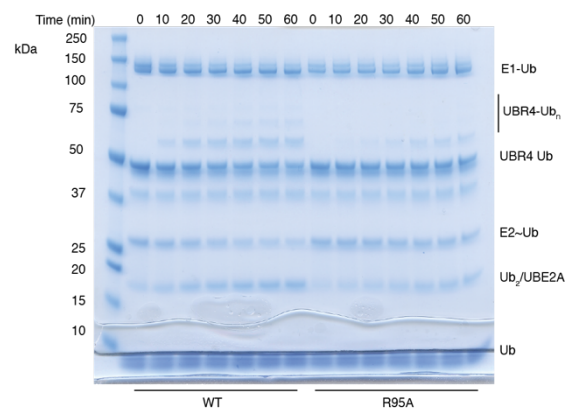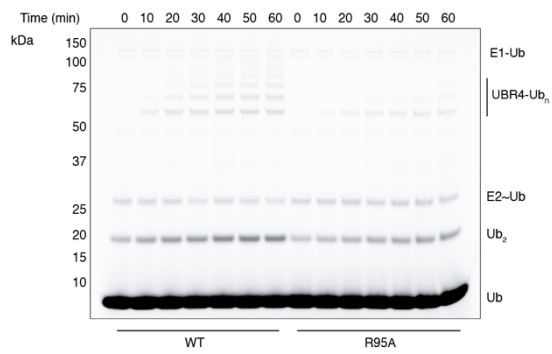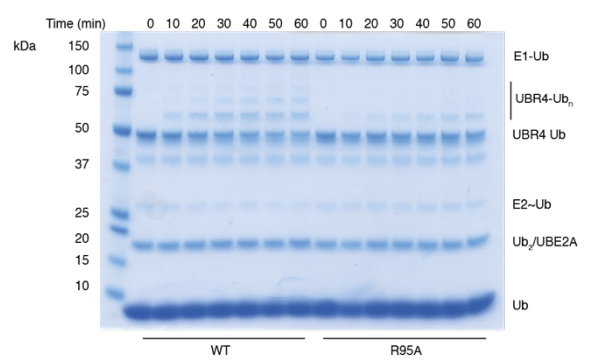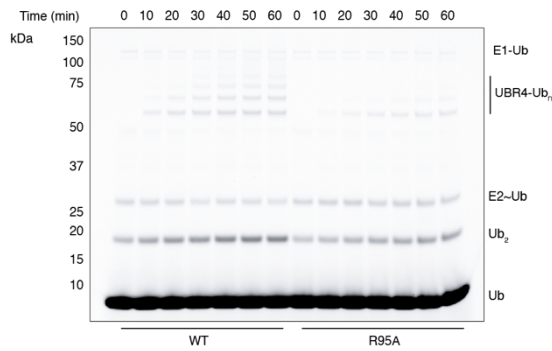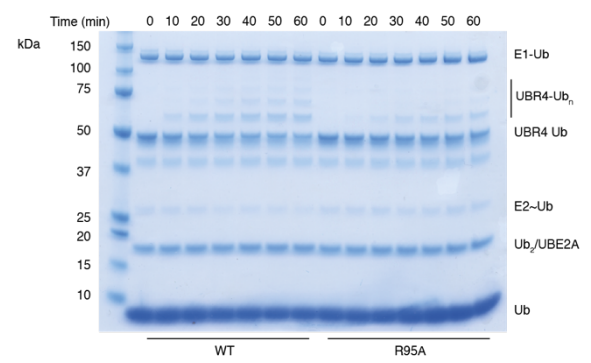

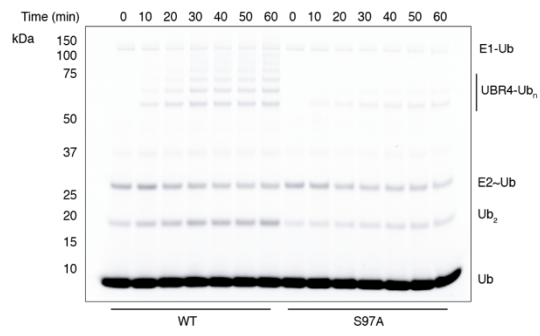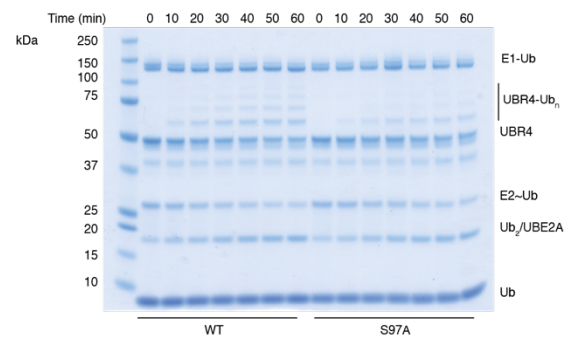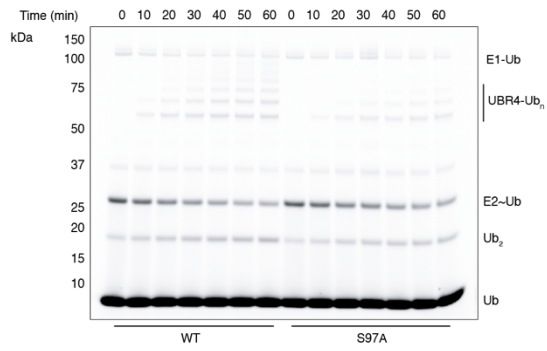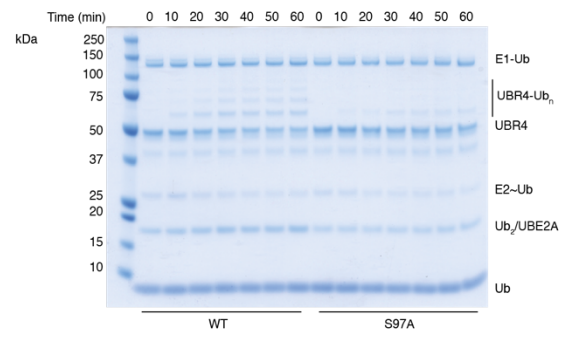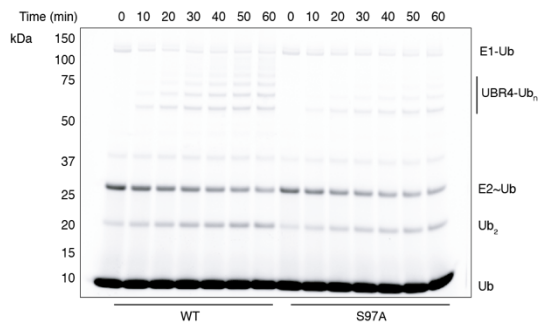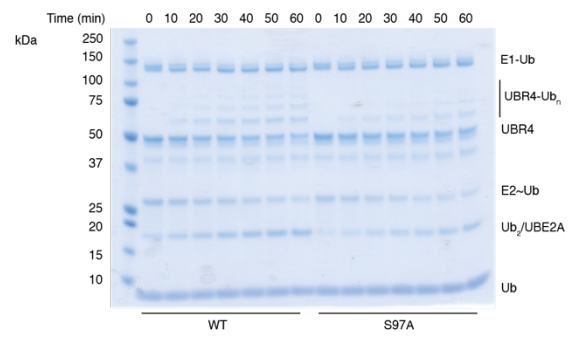

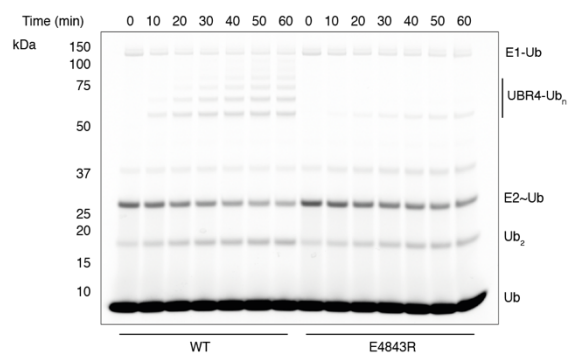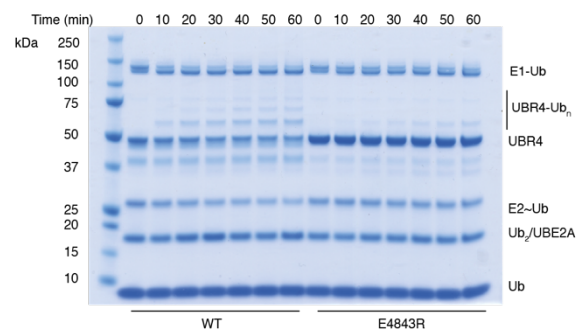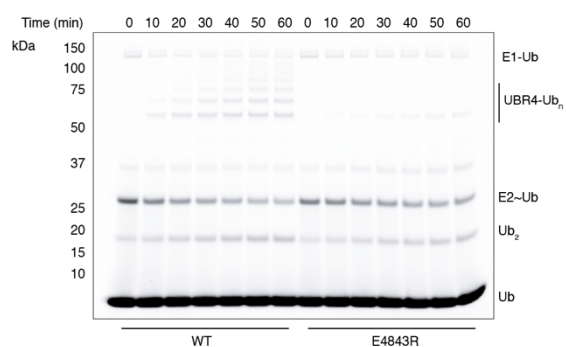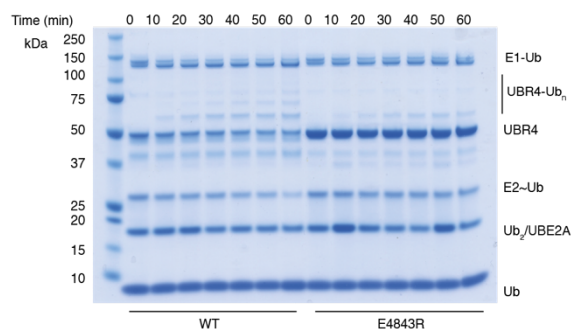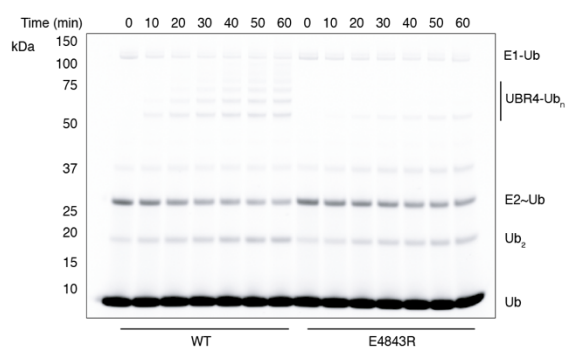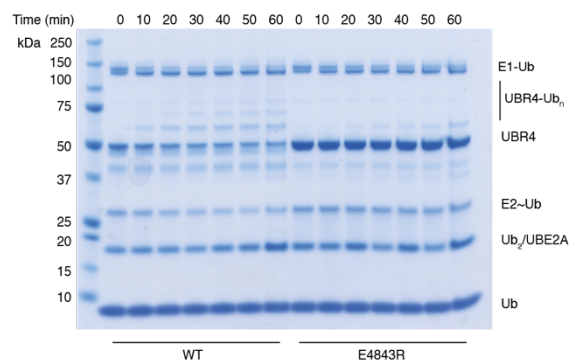

Source data Figure 6e

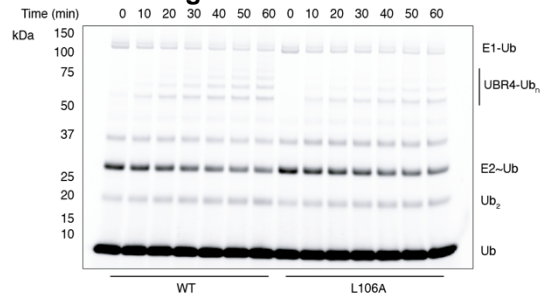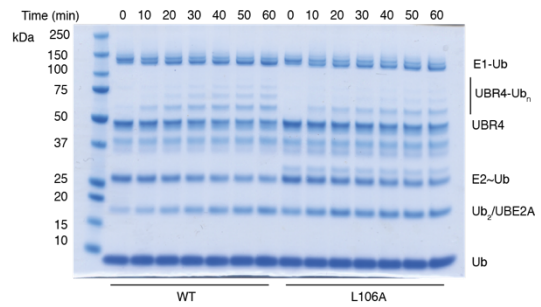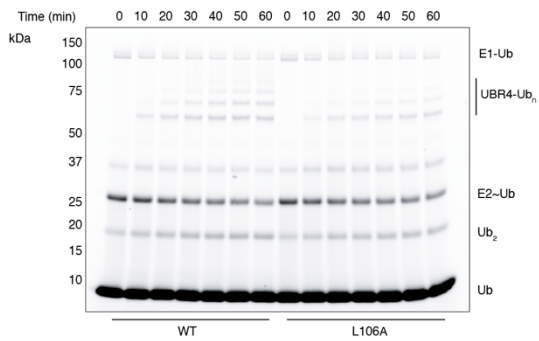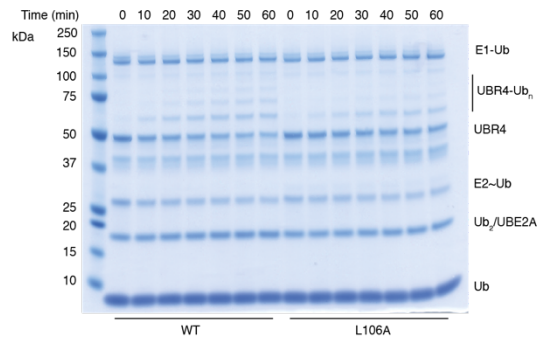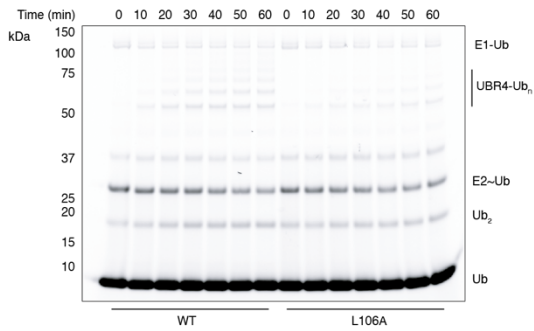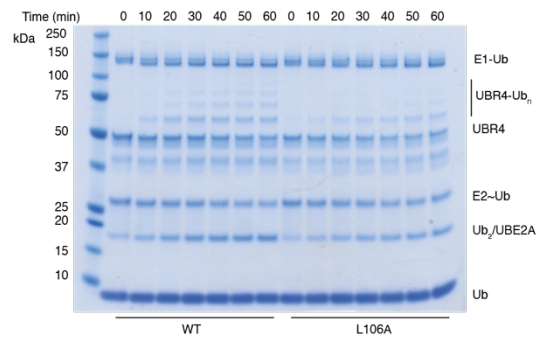

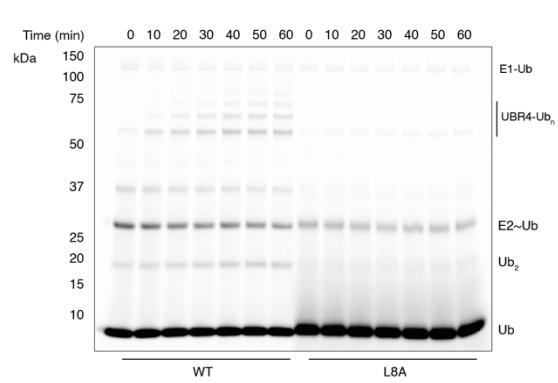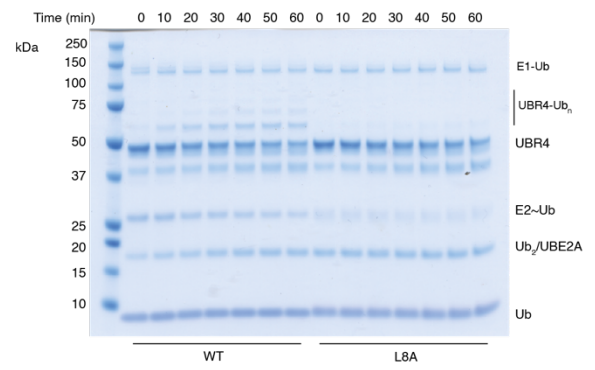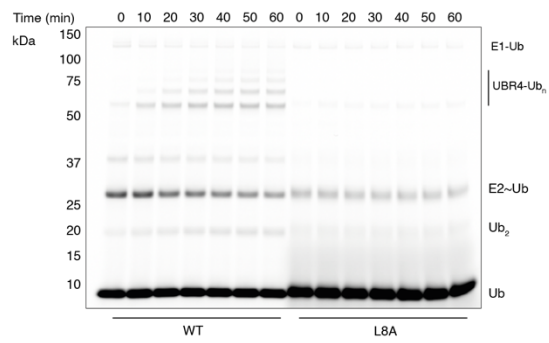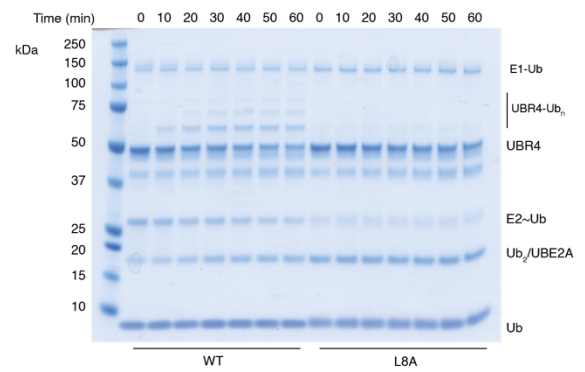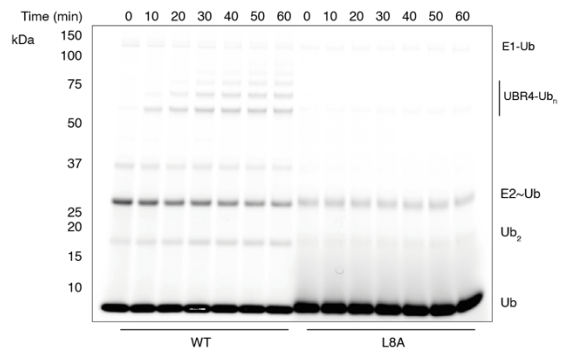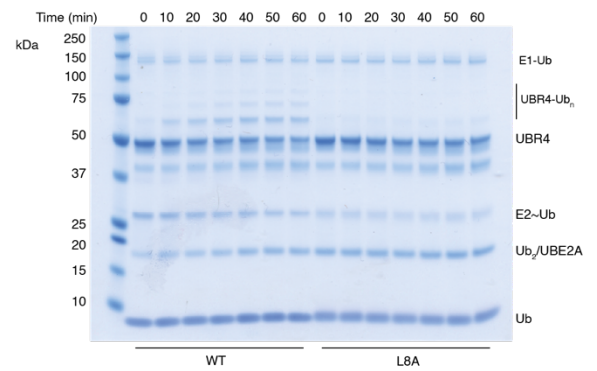

Source data Figure 6f

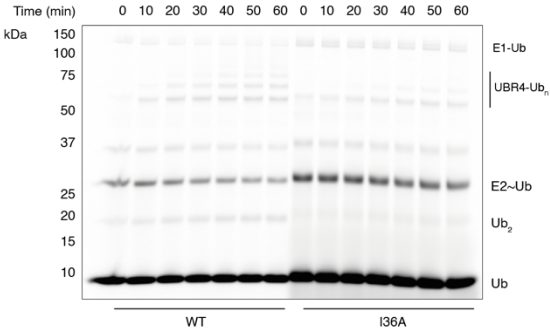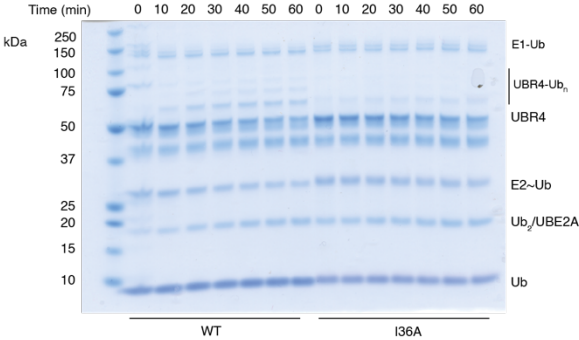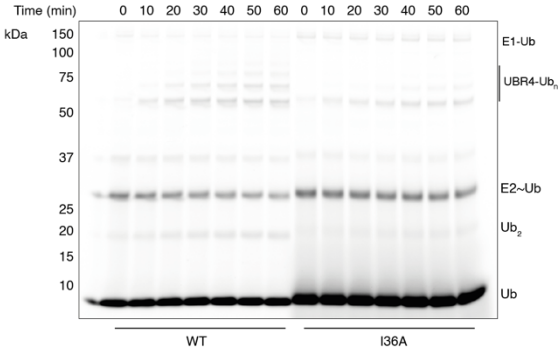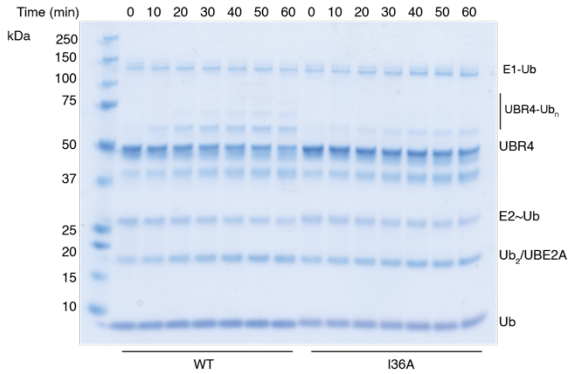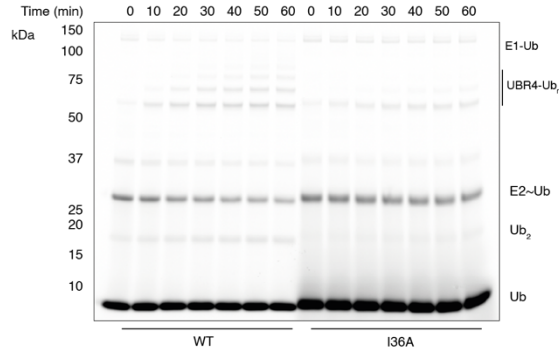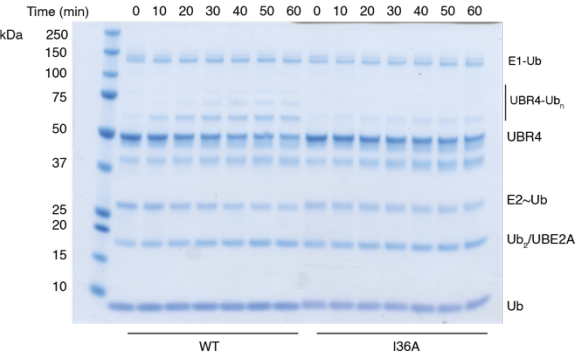

Source data Figure 6g

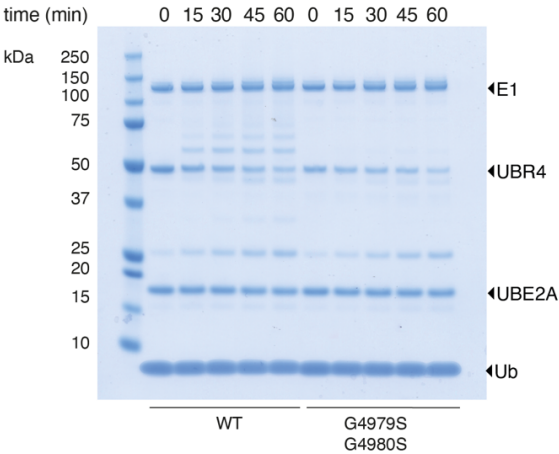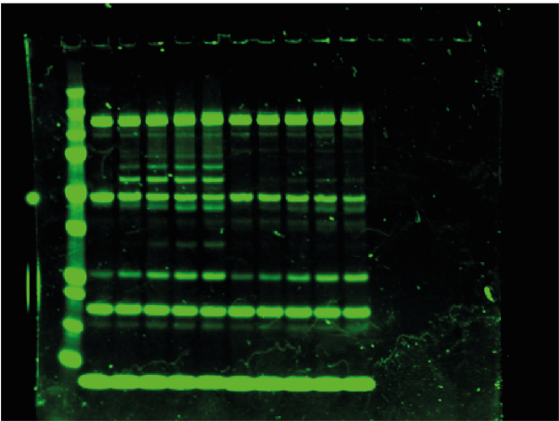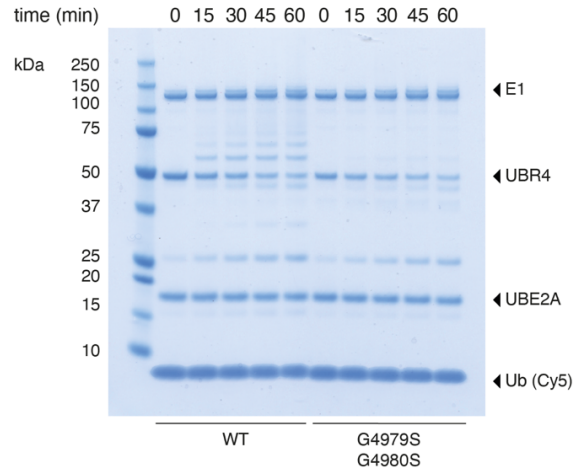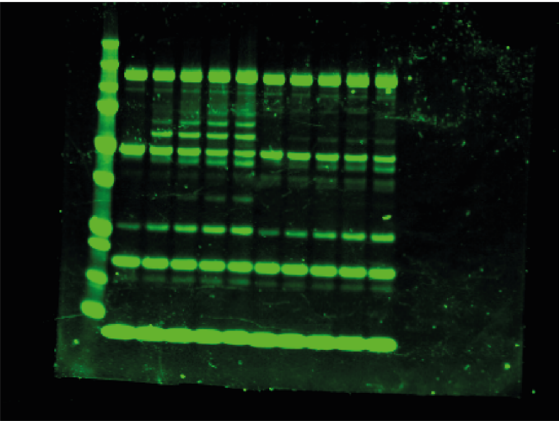

Supplement: Supplementary file 6 — Full gels for Fig. 6b,e–g. [file 41594_2023_1192_MOESM6_ESM.pdf]

Source data Extended Data Figure 2b

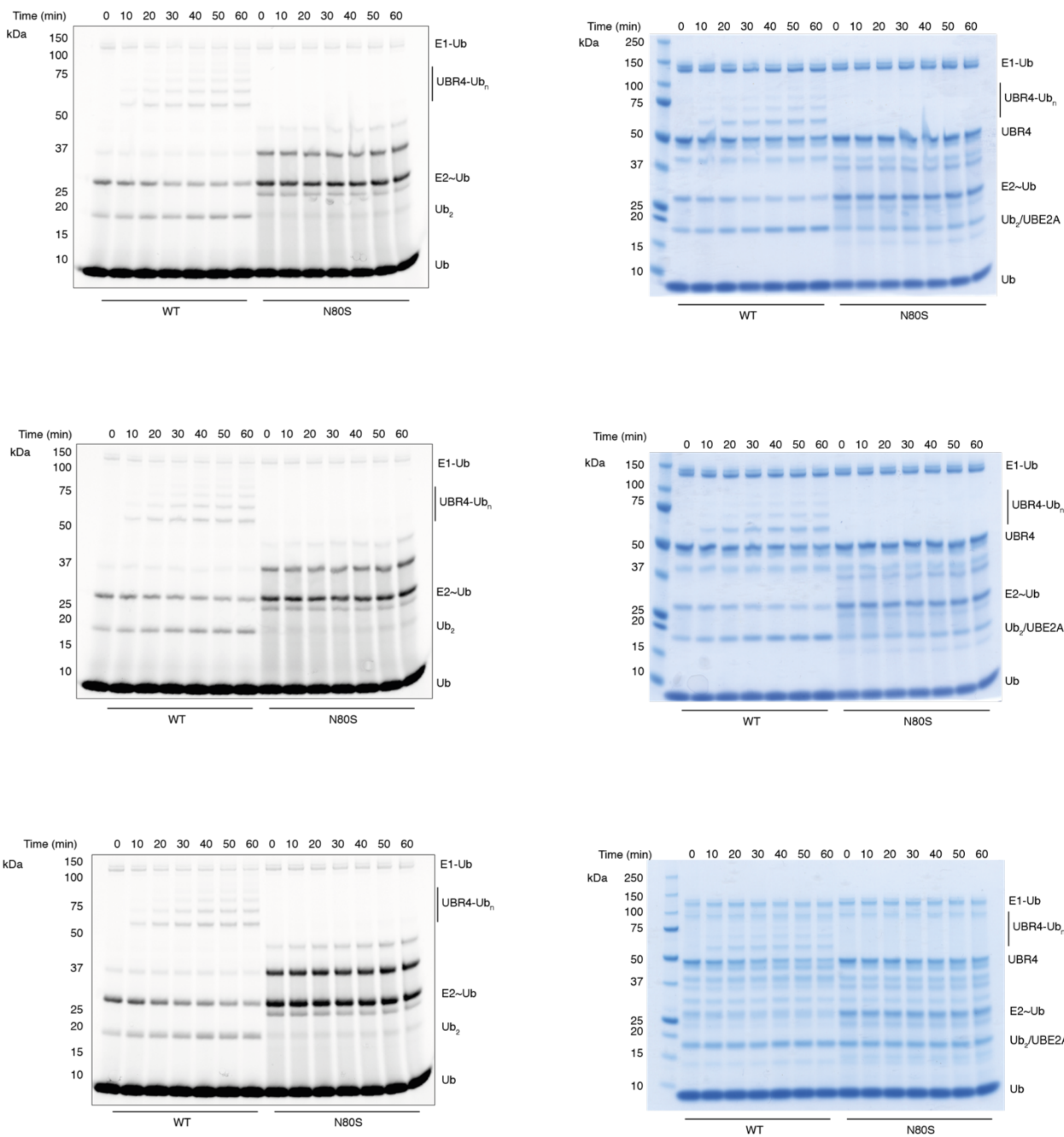

Source data Extended Data Figure 2c

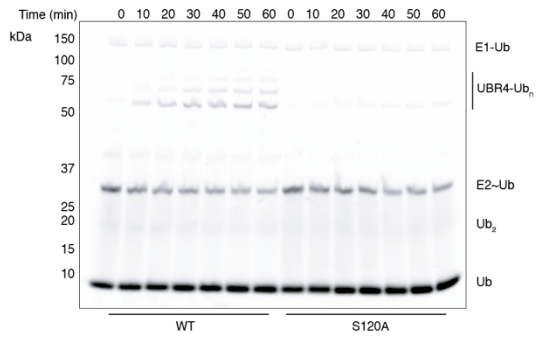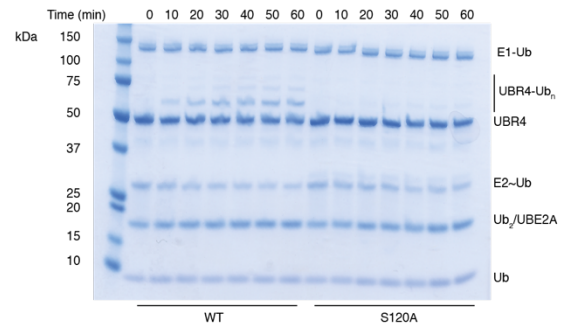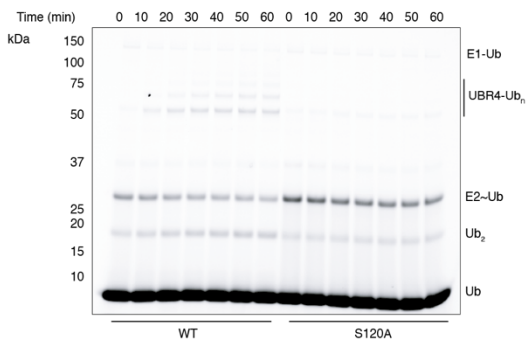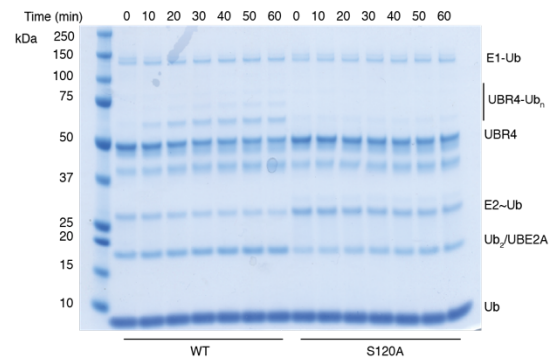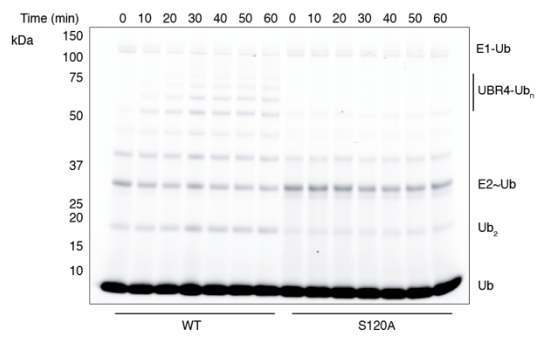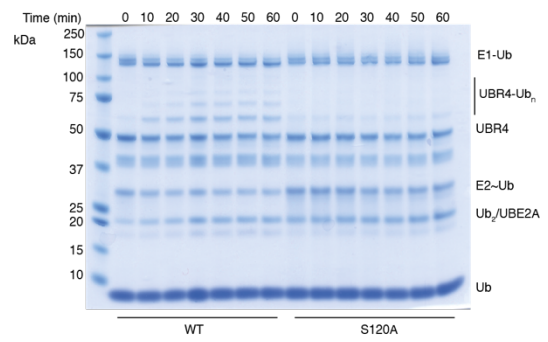

Supplement: Supplementary file 7 — Full gels for Extended Data Fig. 2b,c. [file 41594_2023_1192_MOESM7_ESM.pdf]

**Source data Extended Data Figure 3b**

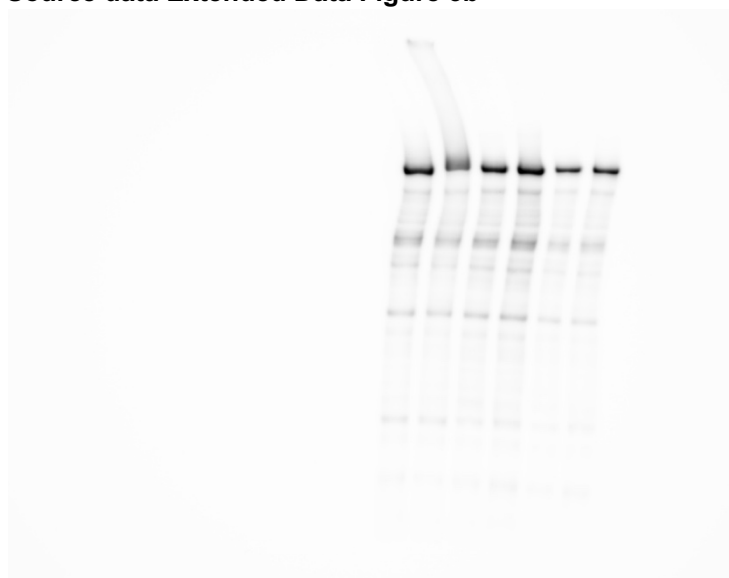

Supplement: Supplementary file 8 — Full blot for Extended Data Fig. 3b. [file 41594_2023_1192_MOESM8_ESM.pdf]

Source data Extended data Figure 7d

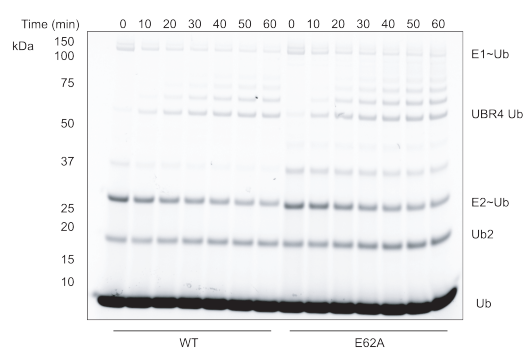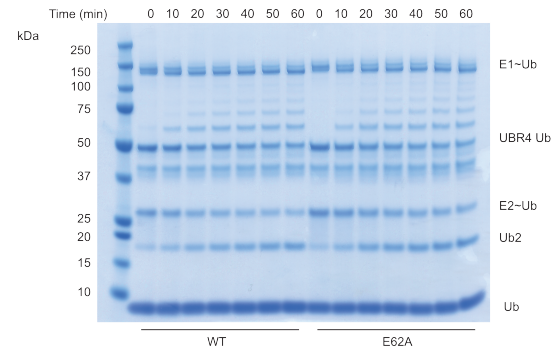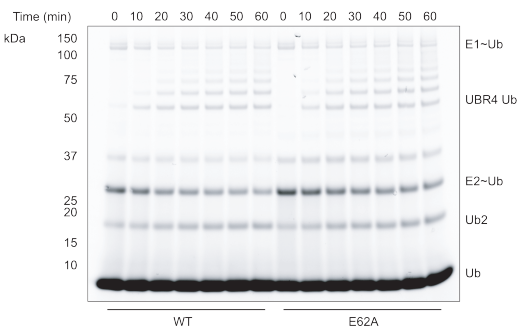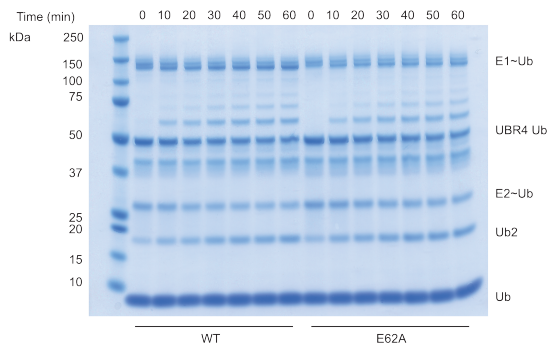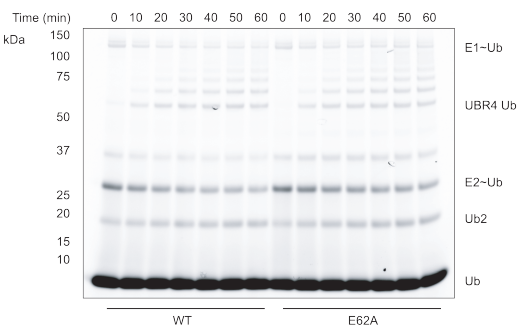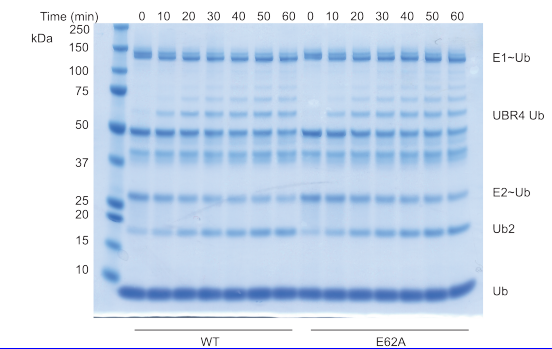

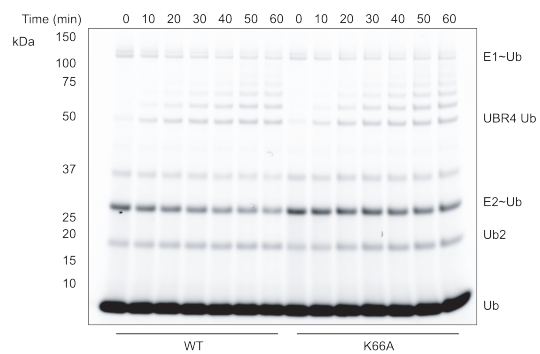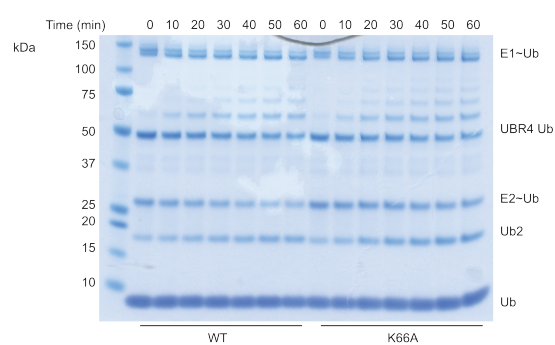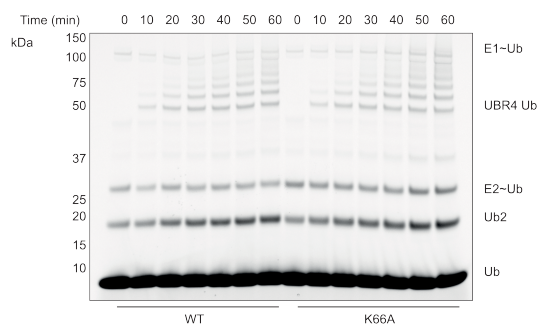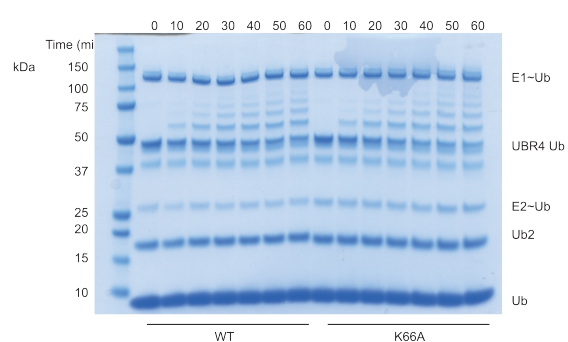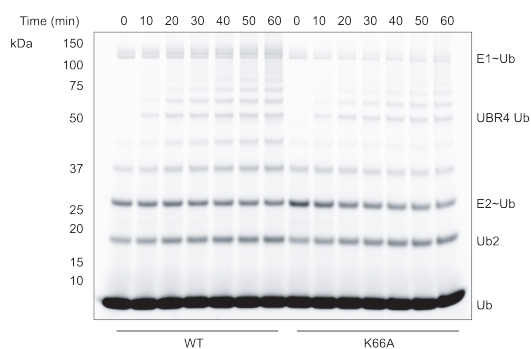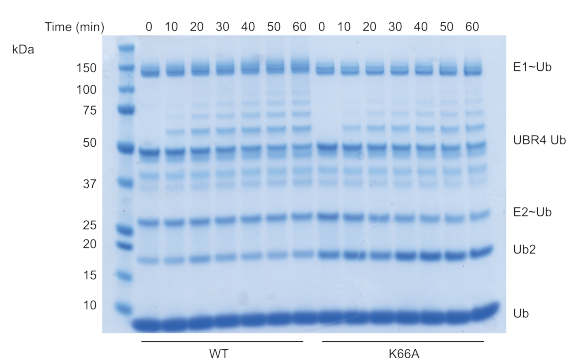

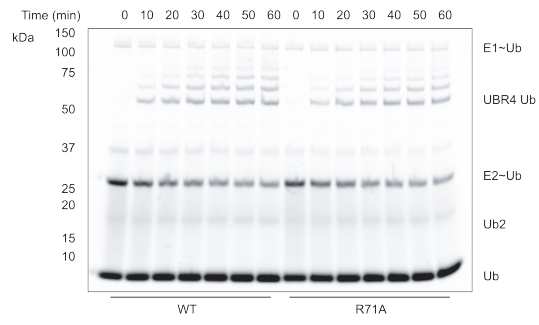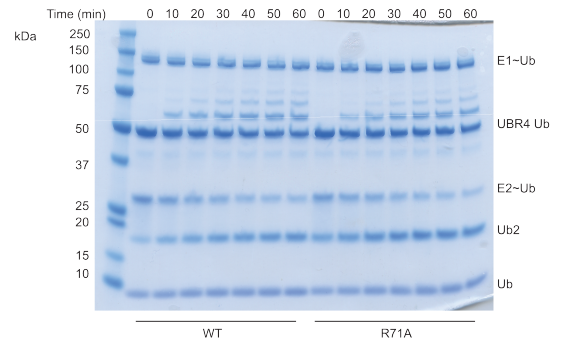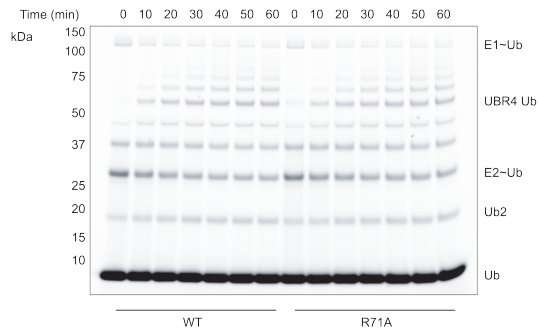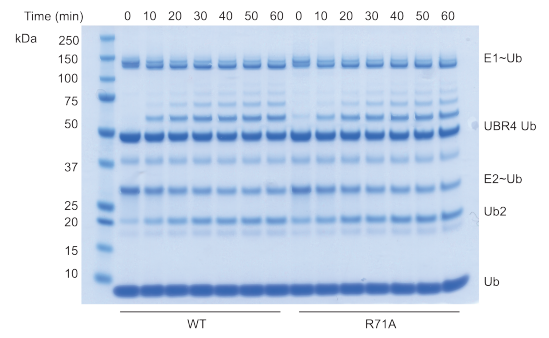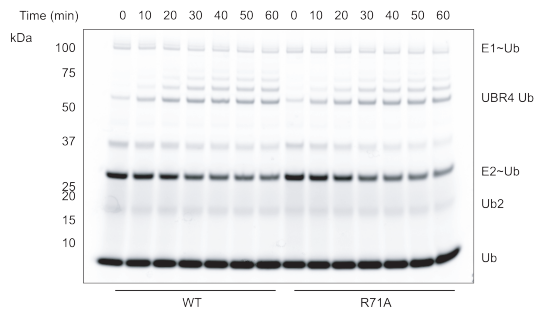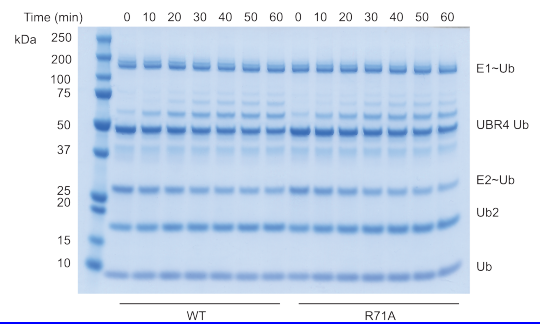

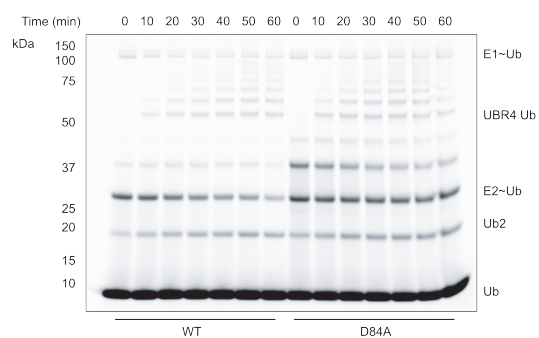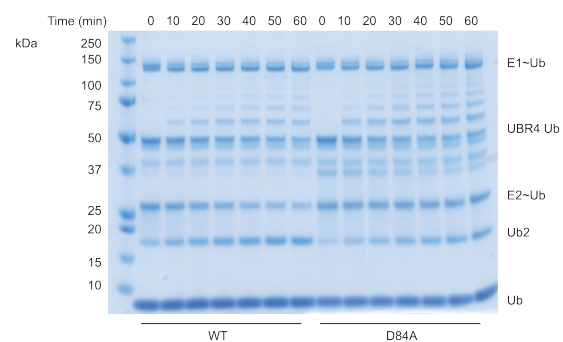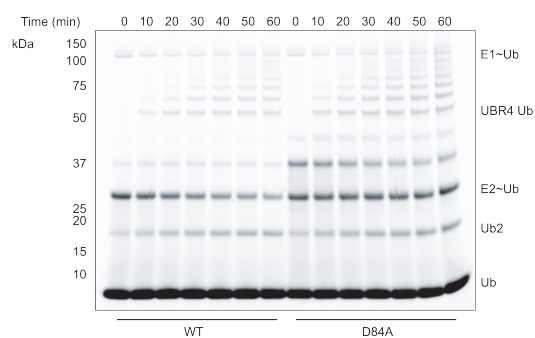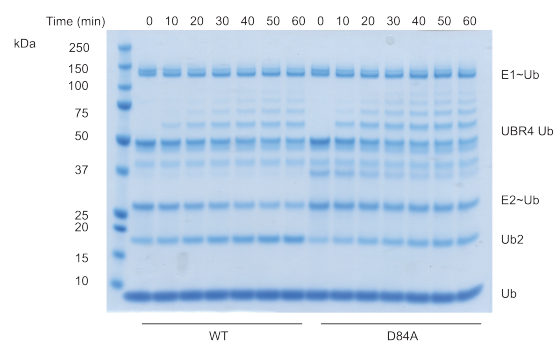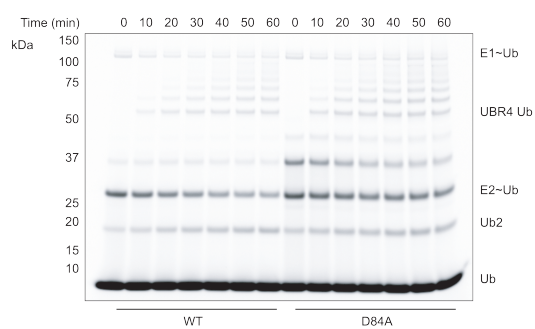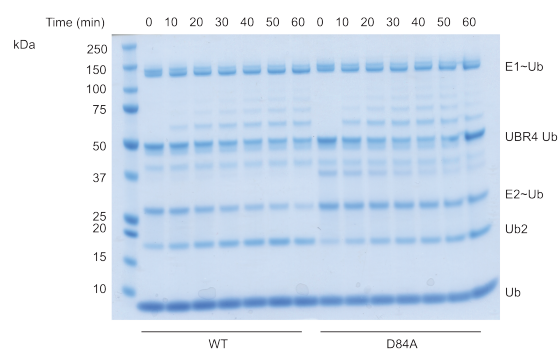

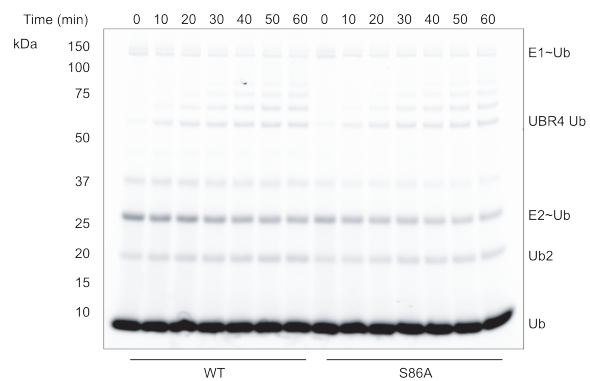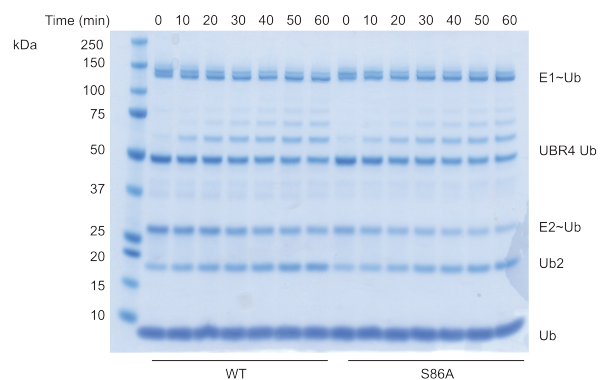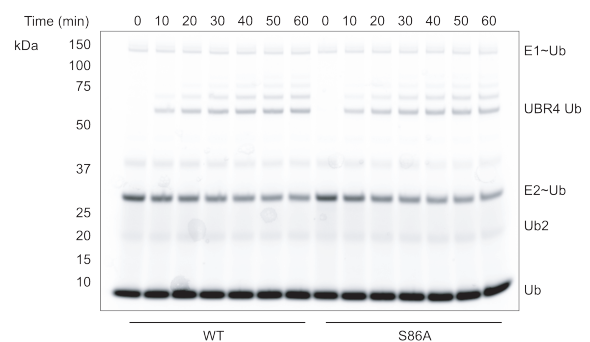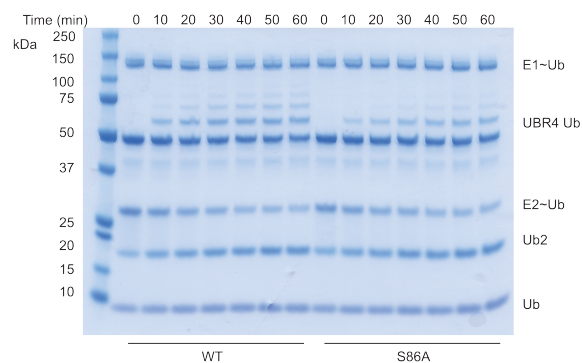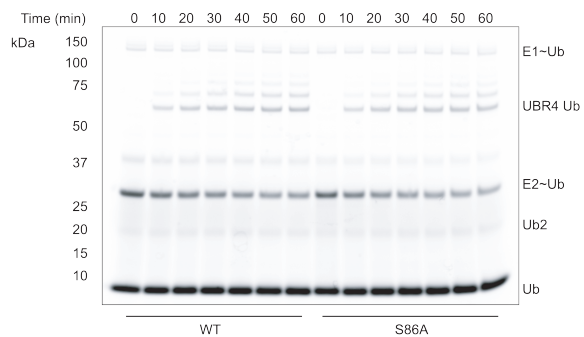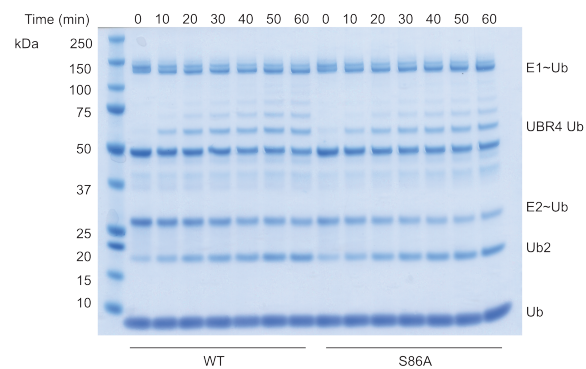

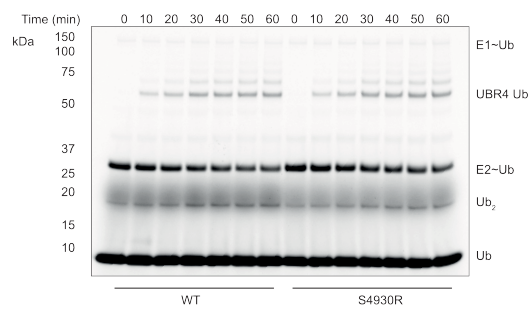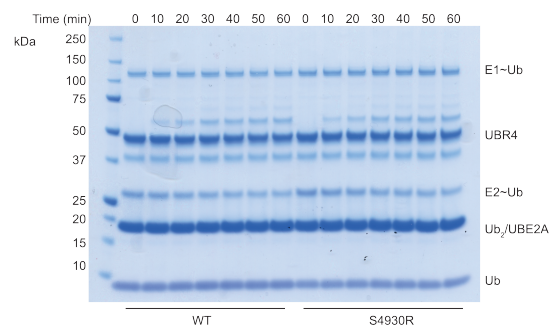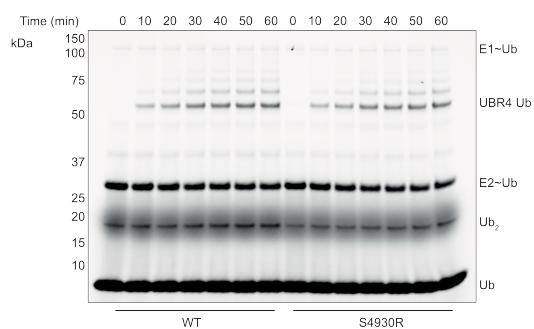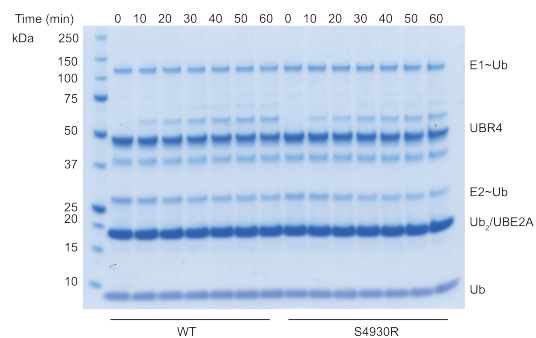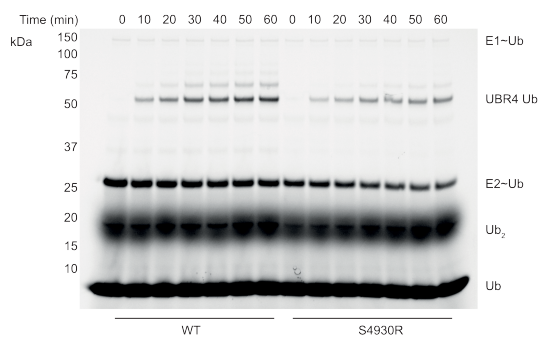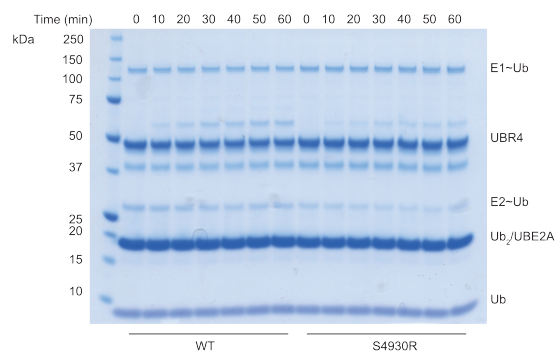

Supplement: Supplementary file 10 — Source data for Extended Data Fig. 7d. [file 41594_2023_1192_MOESM10_ESM.pdf]
