# Supplementary material for: UBE2A and UBE2B are recruited by an atypical E3 ligase module in UBR4
Source: Nat Struct Mol Biol. 2024 Jan 5;31(2):351–63. doi: 10.1038/s41594-023-01192-4 (PMC10873205; doi:10.1038/s41594-023-01192-4)

Source data Extended Data Figure 5b

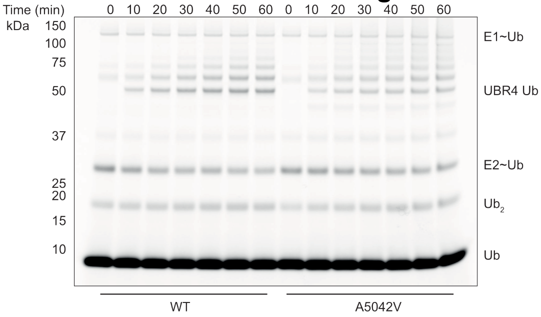

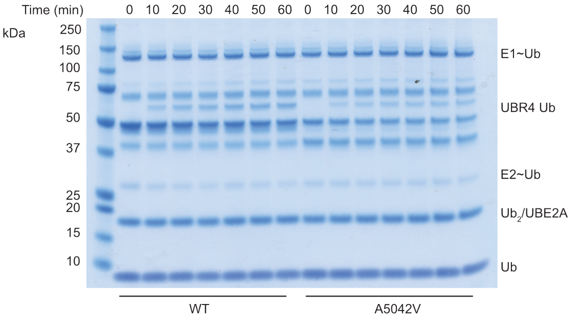

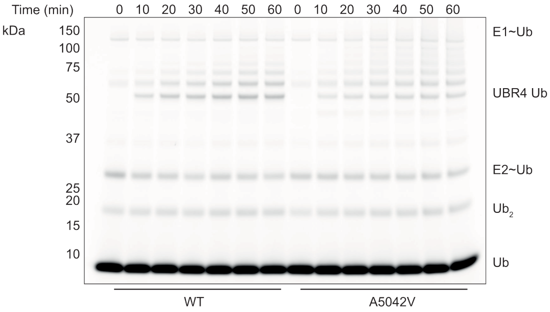

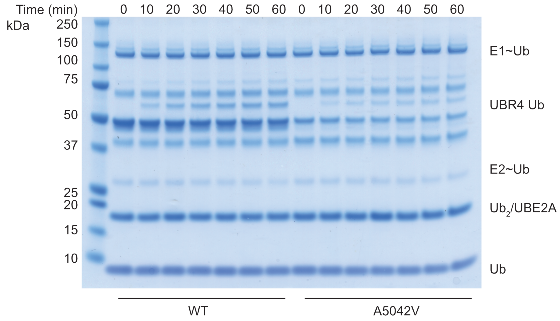

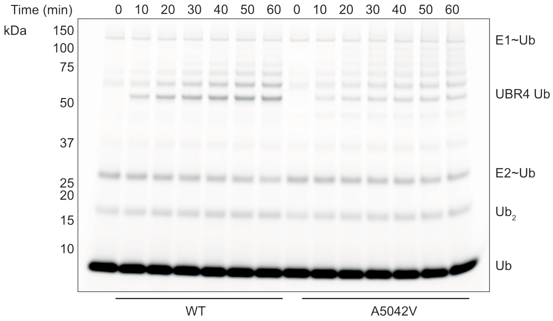

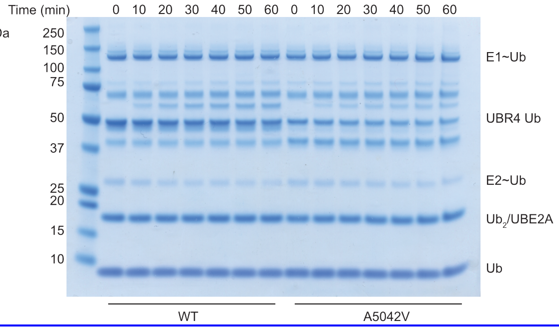

Source data Extended data Figure 5d

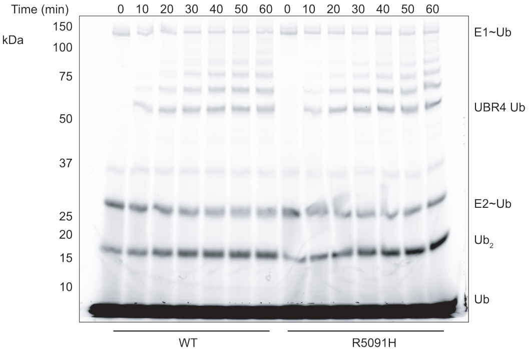

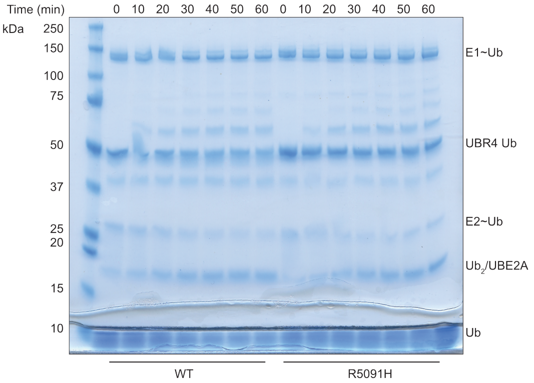

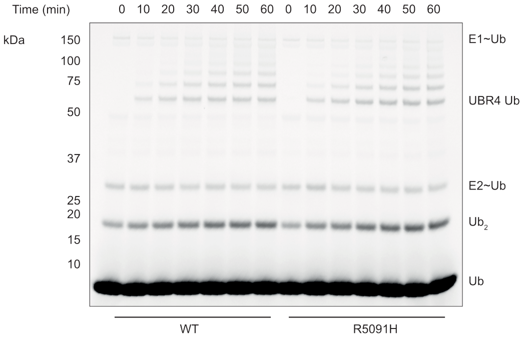

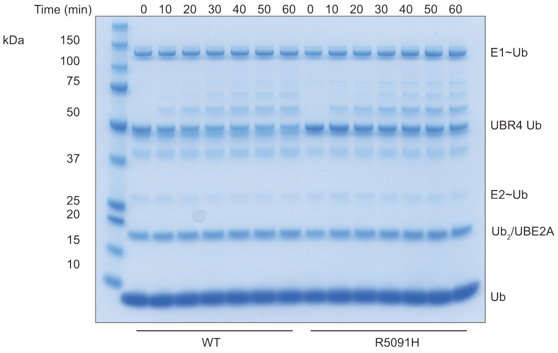

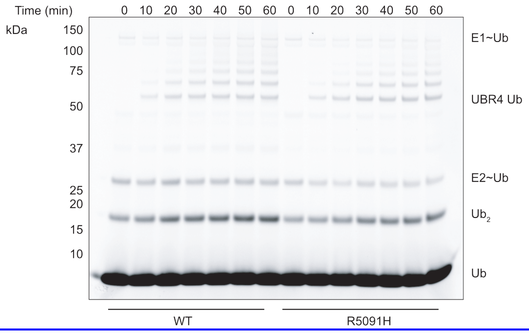

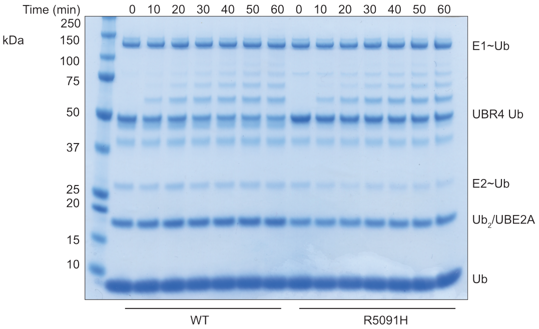

Source data Extended data Figure 5f

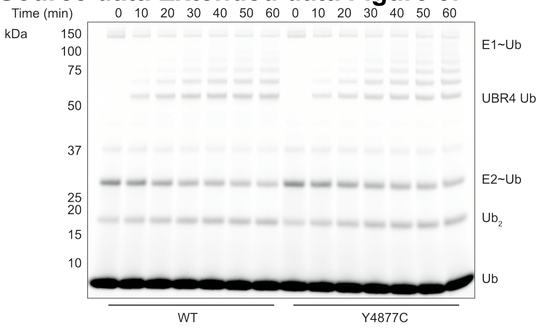

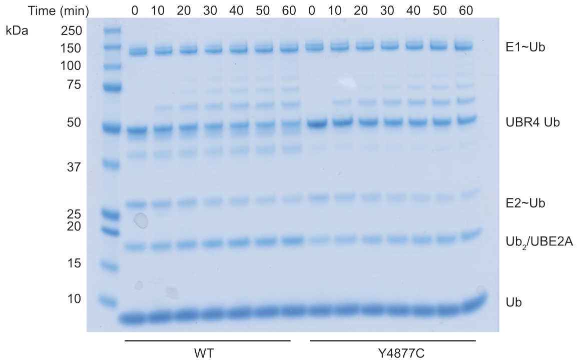

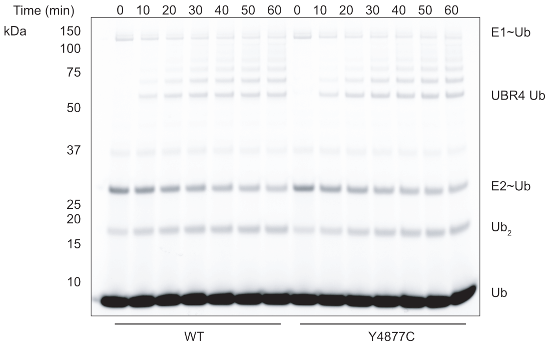

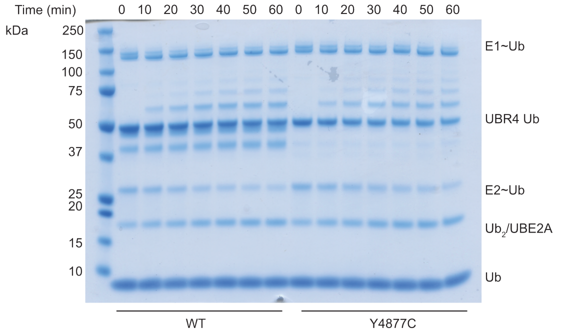

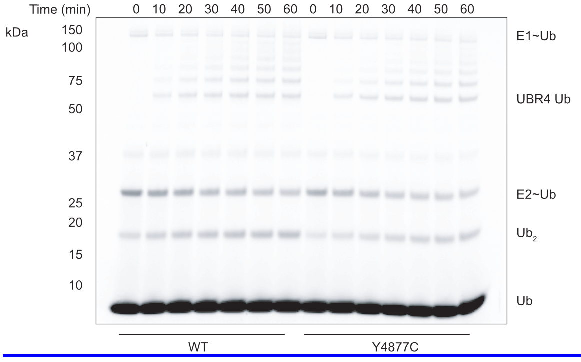

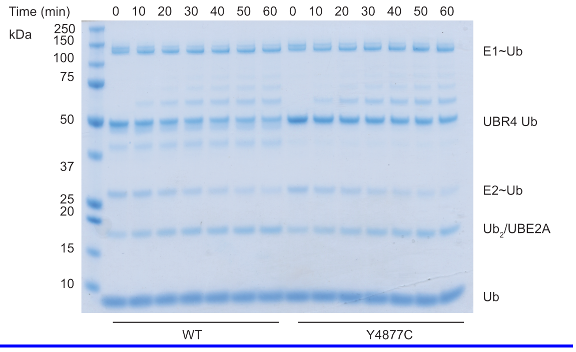

Supplement: Supplementary file 9 — Source data for Extended Data Fig. 5b,d,f. [file 41594_2023_1192_MOESM9_ESM.pdf]
