# Supplementary material for: UBE2A and UBE2B are recruited by an atypical E3 ligase module in UBR4
Source: Nat Struct Mol Biol. 2024 Jan 5;31(2):351–63. doi: 10.1038/s41594-023-01192-4 (PMC10873205; doi:10.1038/s41594-023-01192-4)

Source data Extended data Figure 8b

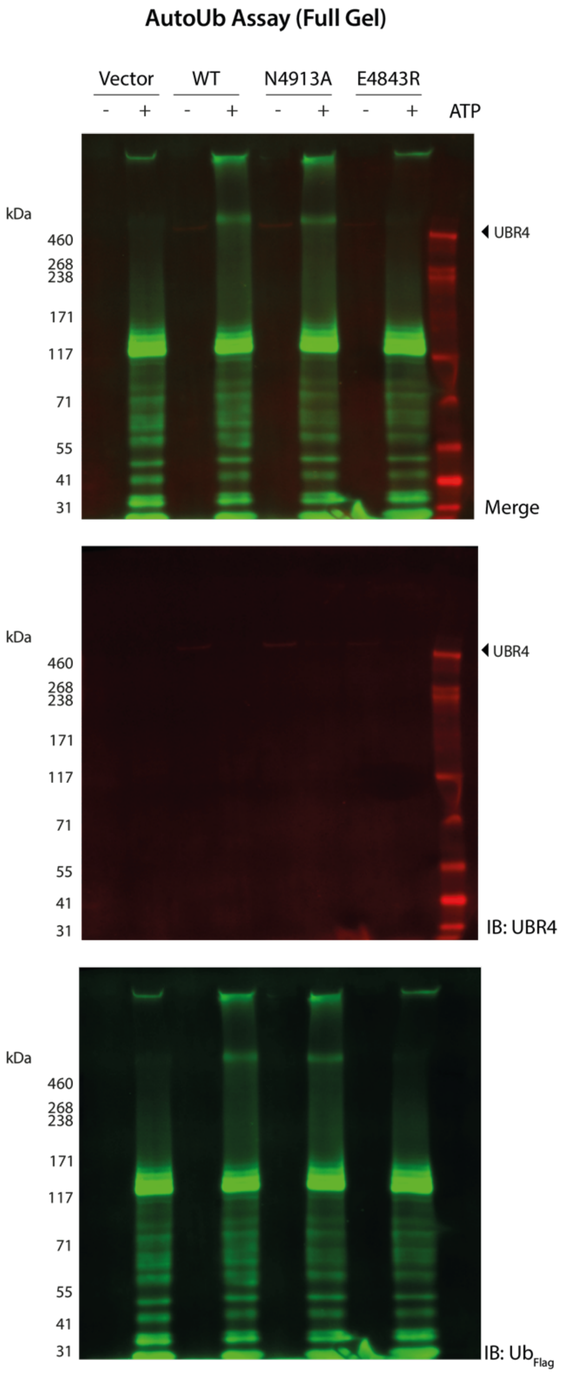

Source data Extended data Figure 8c

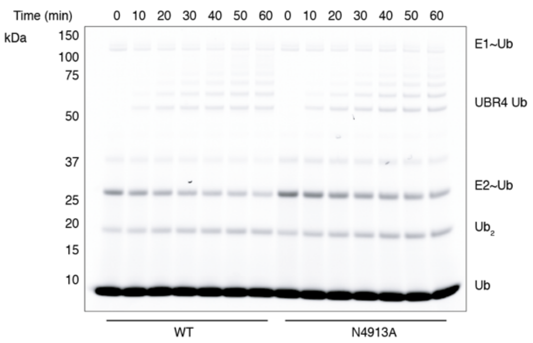
Time (min)
0 10 20 30 40 50 60 0 10 20 30 40 50 60
kDa
150
100
75
50
37
25
20
15
10
E1~Ub
UBR4 Ub
E2~Ub
Ub2
Ub
WT
N4913A

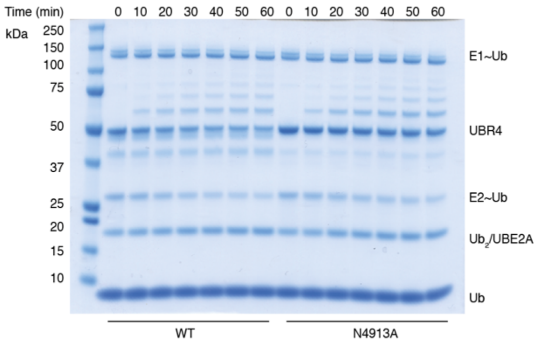
Time (min)
0 10 20 30 40 50 60 0 10 20 30 40 50 60
kDa
250
150
100
75
50
37
25
20
15
10
E1~Ub
UBR4
E2~Ub
Ub2/UBE2A
Ub
WT
N4913A

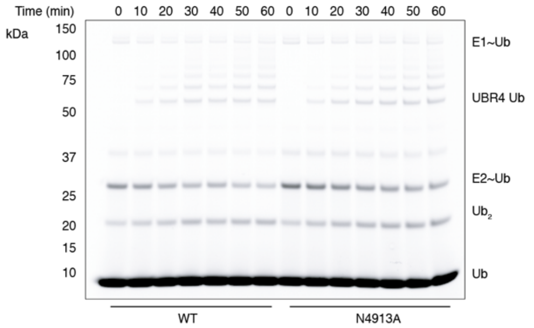
Time (min)
0 10 20 30 40 50 60 0 10 20 30 40 50 60
kDa
150
100
75
50
37
25
20
15
10
E1~Ub
UBR4 Ub
E2~Ub
Ub2
Ub
WT
N4913A

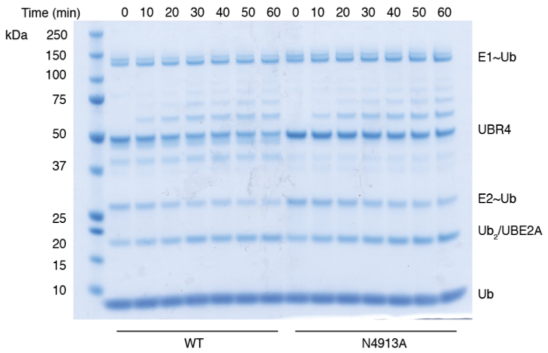
Time (min)
0 10 20 30 40 50 60 0 10 20 30 40 50 60
kDa
250
150
100
75
50
37
25
20
15
10
E1~Ub
UBR4
E2~Ub
Ub2/UBE2A
Ub
WT
N4913A

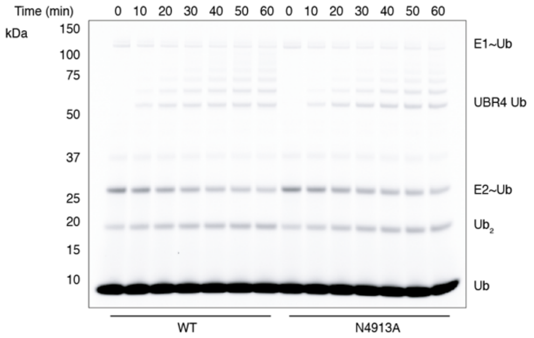
Time (min)
0 10 20 30 40 50 60 0 10 20 30 40 50 60
kDa
150
100
75
50
37
25
20
15
10
E1~Ub
UBR4 Ub
E2~Ub
Ub2
Ub
WT
N4913A

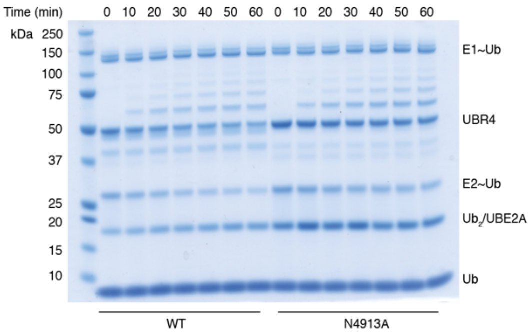
Time (min)
0 10 20 30 40 50 60 0 10 20 30 40 50 60
kDa
250
150
100
75
50
37
25
20
15
10
E1~Ub
UBR4
E2~Ub
Ub2/UBE2A
Ub
WT
N4913A

Source data Extended data Figure 8d

Repeat 1

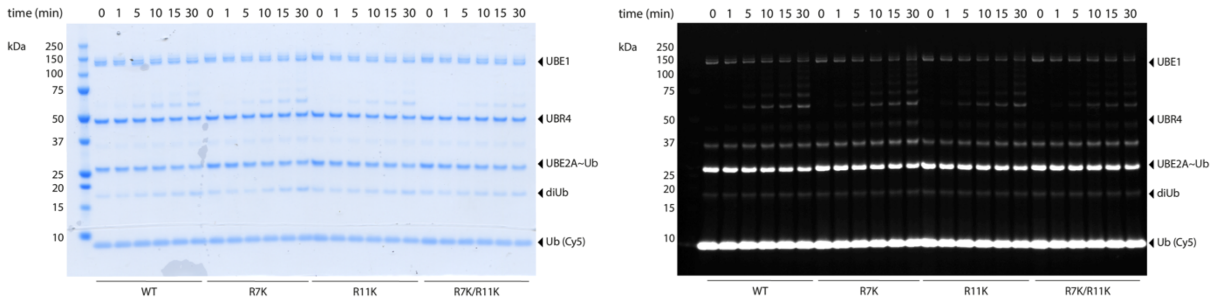

Repeat 2

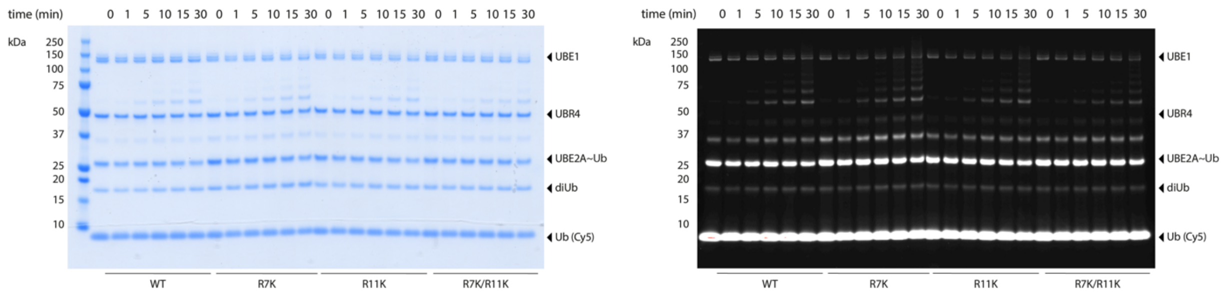

Supplement: Supplementary file 11 — Source data for Extended Data Fig. 8b–d. [file 41594_2023_1192_MOESM11_ESM.pdf]
