# Supplementary material for: UBE2A and UBE2B are recruited by an atypical E3 ligase module in UBR4
Source: Nat Struct Mol Biol. 2024 Jan 5;31(2):351–63. doi: 10.1038/s41594-023-01192-4 (PMC10873205; doi:10.1038/s41594-023-01192-4)

Source data Extended data Figure 9a

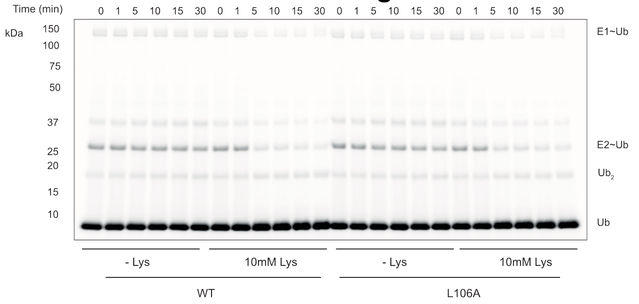

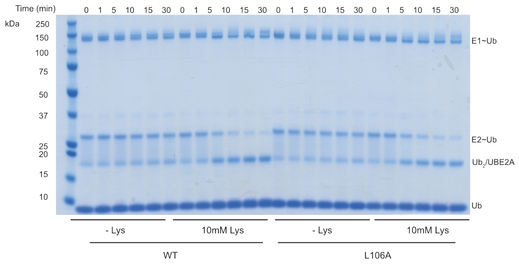

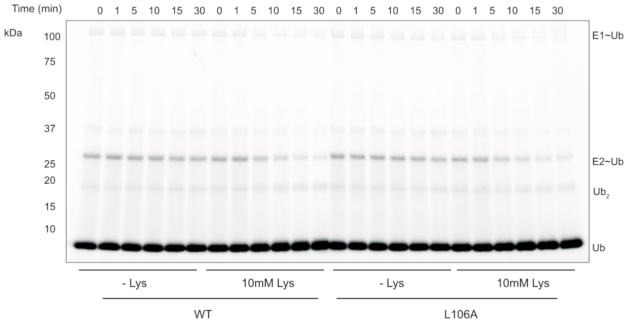

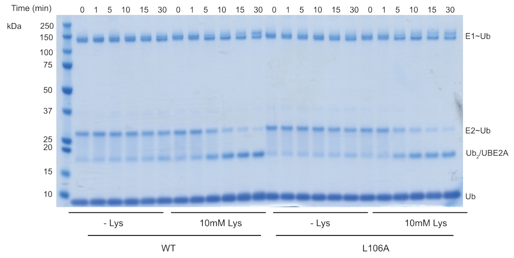

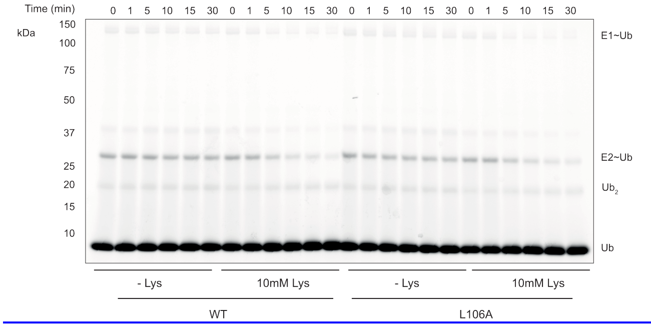

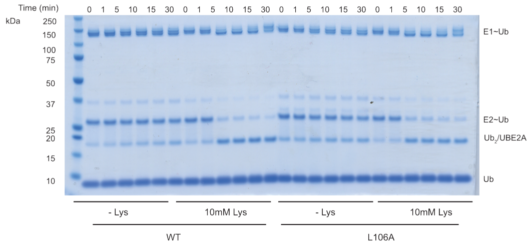

Source data Extended data Figure 9b

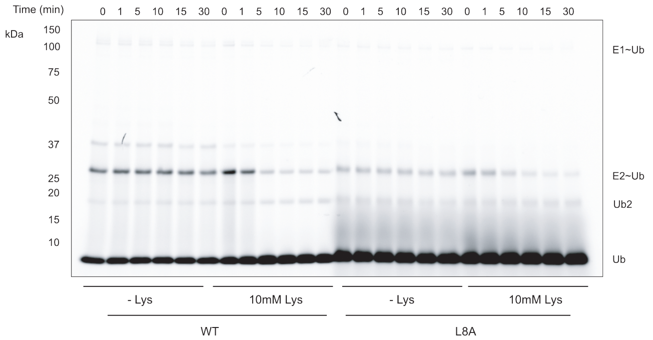

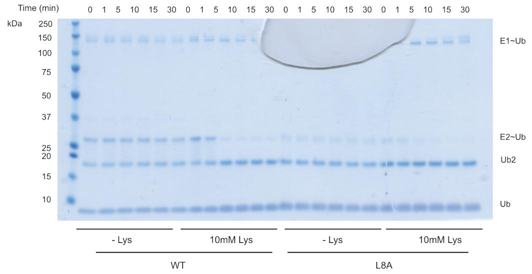

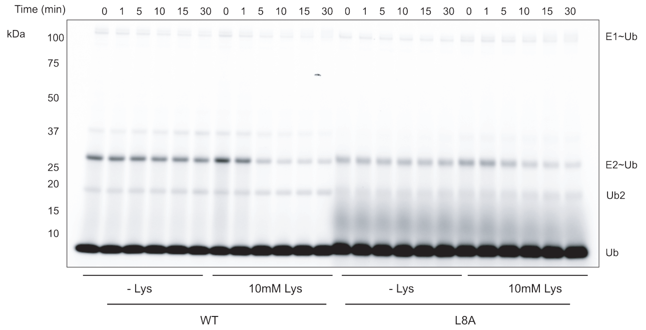

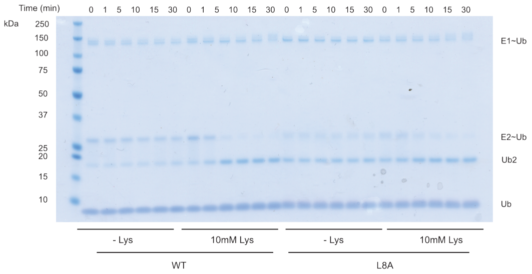

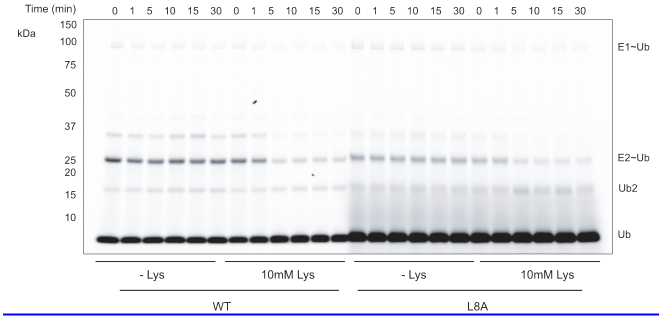

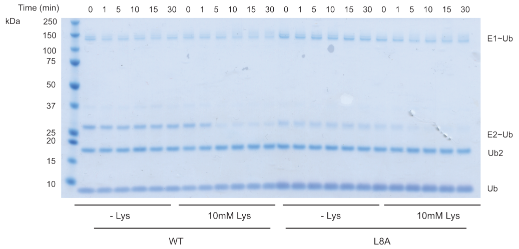

Source data Extended data Figure 9c

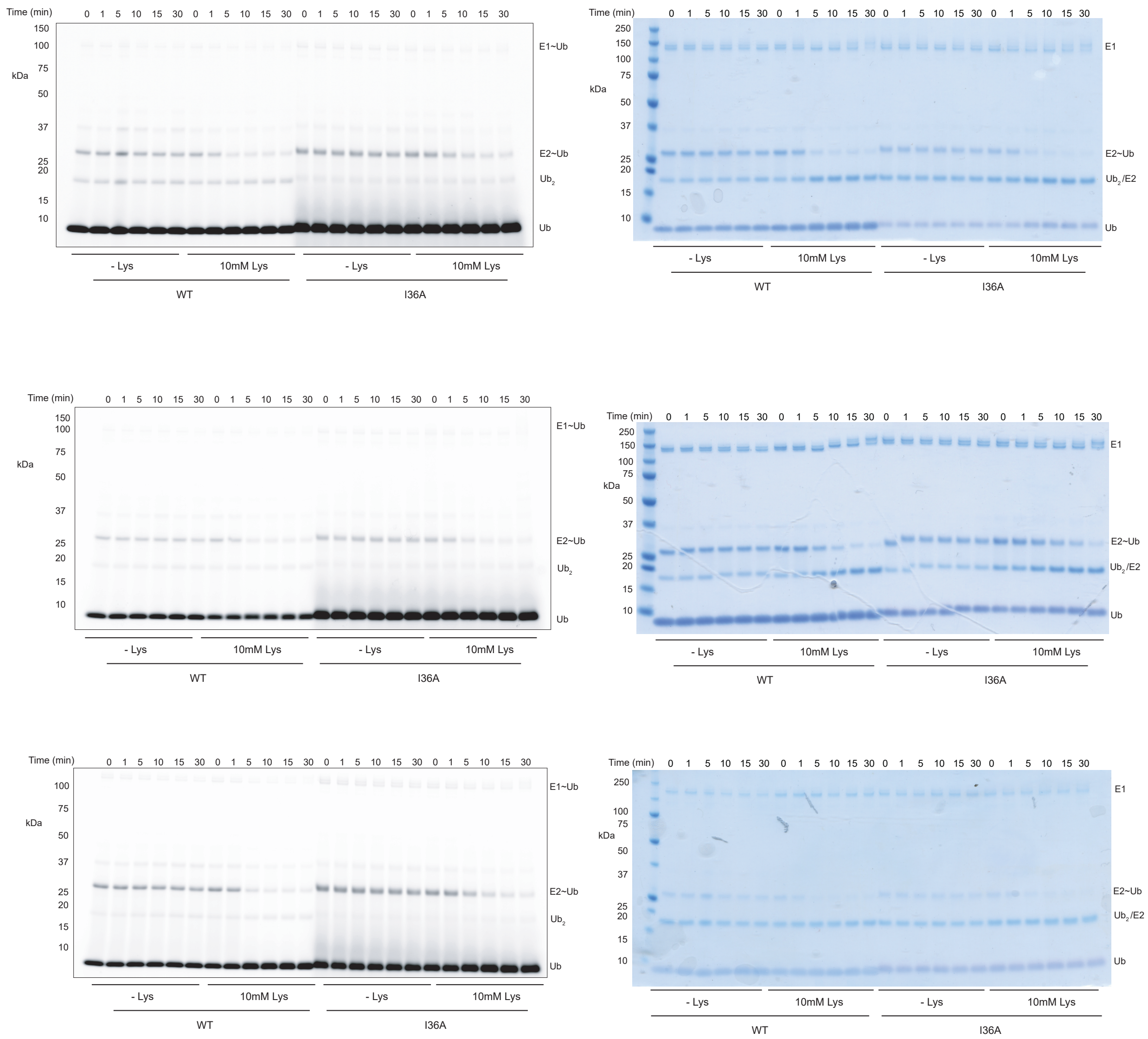

Supplement: Supplementary file 12 — Source data for Extended Data Fig. 9a–c. [file 41594_2023_1192_MOESM12_ESM.pdf]
